# ProFormer: generalizable classification of single-cell and plasma proteomes using deep learning

**DOI:** 10.1038/s41467-026-78143-2

**Published:** 2026-10-05

**Authors:** Karl K. Krull, Arlene Kühn, Julia Höhn, Syed Azmal Ali, Martin H. Otterbein, Titus J. Brinker, Jeroen Krijgsveld

**Affiliations:** 1 Proteomics of Stem Cells and Cancer, German Cancer Research Center (DKFZ), Heidelberg, Germany; 2 Heidelberg University, Medical Faculty, Heidelberg, Germany; 3 Heidelberg University, Faculty of Biosciences, Heidelberg, Germany; 4 Digitial Prevention, Diagnostics and Therapy Guidance, German Cancer Research Center (DKFZ), Heidelberg, Germany

**Keywords:** Machine learning, Proteome informatics, Diagnostic markers

## Abstract

Proteins are the main drivers of cell function and disease, making proteomics a powerful technique for biomarker discovery and defining cell identity. While current technologies can profile thousands of proteins and reach single-cell sensitivity, limited throughput restricts applications such as robust classification of large biological or clinical cohorts. To close this gap, we present a deep-learning (DL) approach for the direct analysis of mass spectrometric (MS) data, assigning proteomic profiles to sample identity. Specifically, we introduce the ProFormer, a transformer pipeline that classifies samples by tabular MS1-level features derived from peptide ions, eliminating the need for time-consuming data interpretation. The ProFormer outperforms traditional machine-learning and image-based convolutional neural networks (CNNs), demonstrating enhanced classification and generalization over 14 evaluated architectures. We further provide detailed insights into how the ProFormer dynamically aggregates MS1 data, while preserving signal contribution and enabling explainable AI at single-peptide resolution. Applied to diverse LC-MS data sets, the ProFormer accurately classified single-cell proteomes by cell type, cycle stage or differentiation trajectory, as well as patient disease status from plasma proteomes. Thereby, the ProFormer provides a versatile and rapid framework for the classification of proteomic data, with important implications for patient stratification, early detection and single-cell analysis.

## Abstract (web summary)

Low throughput and extensive data processing in current proteomic workflows hinder rapid sample classification. Here, the authors introduce a deep-learning approach that directly accepts mass spectrometry-derived peptide ion profiles to rapidly classify cell states or patient disease status.

## Introduction

Proteins control nearly all biochemical processes in cells, and their expression is dynamically regulated to meet cellular demands during development or in response to external cues. The collective set of proteins that make up the proteome can therefore be regarded as a close representation of the cell’s functional phenotype, and its investigation gives detailed insight into the role of proteins in basic cellular processes and in disease^1^. To determine proteome composition, most studies adopt a bottom-up approach, analyzing peptides obtained by proteolytic digestion, as these are amenable to detailed characterization using proteomic techniques^2,3^. This typically entails peptide separation based on retention time in liquid chromatography (LC) systems and in mass spectrometers (MS) that determine their mass-to-charge (m/z) ratio, while integration of m/z intensities over time quantifies the peptide ion signal (MS1 level). In a second step, fragmentation of peptide ions generates sequence information that is used to infer protein identity (MS2 level). This strategy has been used in a vast number of studies to understand proteome adaptation in multiple biological and clinical settings. Nonetheless, we recognize two major limitations associated with this process: (I) Iterative sequencing of peptide ions in MS2 is time-consuming and thus limits the speed of analysis methods. (II) Peptide identification is subject to a number of presumptions, which potentially restricts our view on the signal map produced by proteome analysis. Notably, protein identification is essential to many studies to unravel the underlying biological changes, but specific problems may already be addressed by simpler discrimination tasks. In these cases, retention time, m/z, and intensity features obtained from MS analysis at the intact peptide level (i.e., MS1) may serve to define a sample’s peptide ion profile that is characteristic of its proteomic make-up, and there are increasing efforts to utilize this information in deep-learning (DL) methods that classify samples based on their LC-MS fingerprint^4^. Focusing on MS1 information for sample classification has the potential to significantly increase the analysis speed of the proteomic pipeline as it shortcuts data processing and interpretation, which in turn will leverage sample throughput. This distinguishes it from existing classifiers that utilize processed data requiring MS2-based identification of peptides or proteins, and which exhibit poor generalizability due to experiment- and sample-dependent processing biases. In contrast, peptide-agnostic classification of MS1 data may provide greater robustness, creating opportunities for the development of large, generalizing AI models with utility beyond individual laboratories. Such an approach would remain complementary to conventional proteomic analysis, with particular relevance for single-cell annotation and in clinical contexts, where rapid detection of disease states and patient stratification prevail over in-depth proteome characterization. As the most prominent example, convolutional neural networks (CNNs) have been applied to classify proteomic raw data by utilizing the MS1 peptide ion profile as a converted image-like heatmap, discriminating conditions based on spatial peak patterns^4–8^. In this process, data of proximate signals are binned (i.e. that have similar mass or retention time), achieving a uniform grid input that allows employing pre-trained networks^4^, while this procedure aligns individual samples, covering measurement variabilities^6^. However, unlike pixels in real images, peptide ion signals in proteomic data are not related to their surroundings, limiting interpretation based on their localization in mass—retention time—intensity space. Therefore, it is unclear to what extent binning affects data consistency in image pipelines, particularly regarding potential information loss. Moreover, image conversion restricts the underlying DL architecture to a fixed number of features, making them inaccessible to recent advances in MS instrumentation that implement ion mobility as a fourth separation layer^9^. Although previous studies have shown that high accuracies can be achieved using CNNs with a great number of internal layers^4–7^, they lack evaluation by an independent test set. Hence, it remains unclear if compressed proteome images adequately represent data to enable problem generalization and if their use in computationally intensive architectures increases the susceptibility to overfitting. Consequently, it needs to be investigated whether image-based classification of proteomic samples is resistant to domain shifts, such as batch effects, differences in instrument components, and experimental variability. In contrast, and as an innovative alternative, transformer architectures may learn complex patterns without spatial relationships using a self-attention mechanism^10^, and they have achieved remarkable success in various other fields, e.g., for natural language processing^11^, image tasks^12^, and tabular data^13^.

Here, we introduce the ProFormer (Proteome transFormer), a transformer-based DL pipeline, built to accurately classify proteomic samples with high robustness using their tabular peptide ion profile. The flexible input of the ProFormer allows the implementation of any desired number of peptide ion features derived from MS measurements, including ion mobility, and preserves data integrity without prior binning. Using this approach, we demonstrate the strong resilience of the ProFormer to technical batch effects and its ability to resolve the effect size of dose-dependent proteome perturbations in the challenging recognition of a mild IFN-γ treatment. Comparing the ProFormer to a wide landscape of 14 other architectures, we show its superior predictive performance relative to traditional machine-learning (ML), CNNs, and a Vision-Transformer for the analysis of proteomic data. Of practical significance, we explore data attributes determining the ProFormer’s performance and highlight its versatility by accurately classifying single-cell proteomes from several recently published data sets. Finally, in an applied setting, we accomplished particularly high recall in the recognition of patients with severe Covid-19 among other infectious diseases from their blood plasma, and we show that the resulting model is explainable at a single-peptide resolution, indicating the ProFormer’s potential for broader clinical utility in the future.

## Results

### Creation of a transformer-based model for peptide-level classification of proteome samples

**Fig. 1:**
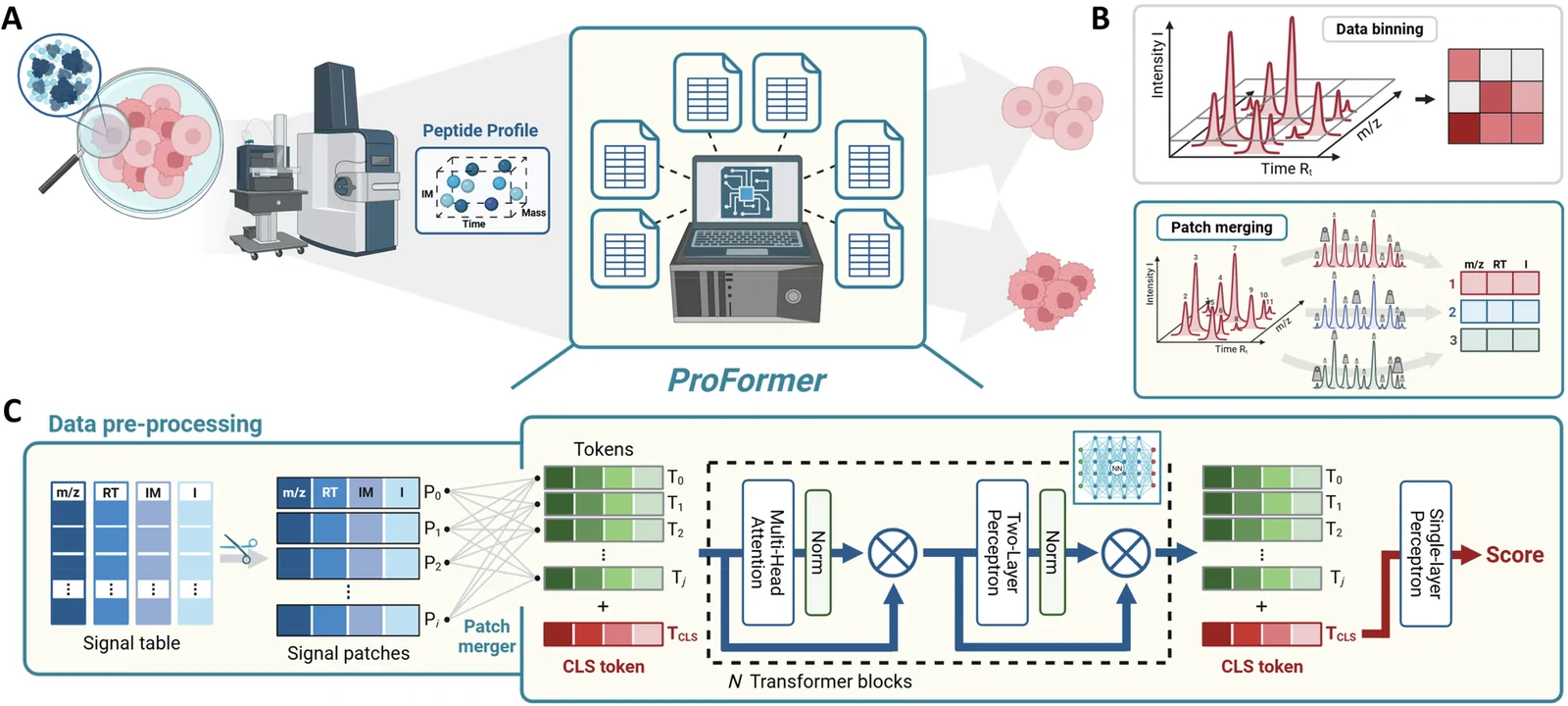
DL-based classification of proteomic samples using the ProFormer. **A** Conceptual design of DL-assisted classification: proteomic samples are characterized by peptide ions that are defined in four features. These data tables can be used to discriminate the profiles of two sample classes by the ProFormer. **B** Comparison of two approaches to reduce the high dimensionality of proteomic data. Top: data binning according to a grid structure of defined dimensions, e.g., to create an image-like heatmap. Bottom: peptide signals are weighted and accumulated to patches using a learnable patch merger that can handle any number of features (IM feature not shown) and that was used in this work to generate tokens as input for a transformer architecture. **C** ProFormer pipeline: Tensors/patches of high-dimensional peptide ion data are extracted and reduced to *j* tokens using a patch merger. The input is complemented by a CLS token and is passed to a number of *N* Encoder blocks (dashed lines). Each Encoder block contains a Multi-head Attention module, followed by a Two-layer Perceptron and interspersed normalization layers. Afterwards, the CLS token is used as a readout in a classifier that consists of a Single-layer Perceptron, which determines the predicted score from the interval [0, 1]. Numbers *j* and *N* are learnable hyperparameters. Figure 1A–C [https://BioRender.com/6qfzpmp] created in BioRender by Krull, KK (2026).

Current proteomic strategies generate high-dimensional data with thousands of identified peptides typically across a limited number of samples (<100 s), rendering this type of data susceptible to overfitting when used as an input for deep neural networks. To challenge this limitation, we developed the ProFormer pipeline, a DL-based proteome classifier that allows discrimination of samples according to their tabular peptide ion profile, defined by any number of MS1-derived features, such as m/z, retention time (RT), ion mobility (IM), and intensity (Fig. 1A). Given the recent success of transformer models across various fields of application, we adopted this architecture, hypothesizing that its ability to globally compare information dependencies would address the challenging adaptation to proteomic data. As a central aspect of our pipeline, we reconsidered the pre-processing step that is required to reduce data dimensionality and generated a tabular input format using a learnable patch merger^14^. In contrast to the application of a fixed grid input, in which proximate peptide ion signals are binned to an image-like heatmap (Fig.1B, top panel), the patch merger divides data into a number of peptide ion subsets (patches) that summarize their information content (Fig. 1B, bottom panel). Since the weighting of patches is learnable, this approach has the potential to represent the data in a more dynamic and information-preserving fashion, and it might thus increase its robustness to sources of data volatility. In our pipeline, the patch merger aggregates peptide ion information in data tokens, used as input for the transformer, while its tabular approach permits the integration of all four MS feature dimensions, benefiting from its versatility in accepting various data characteristics (Fig. 1C). Importantly, this step does not involve the positional encoding of tokens, allowing the pipeline to identify class-specific relations independent of their input sequence. Conceptually, the subsequent deep-learning (DL) architecture utilizes a self-attention mechanism, proceeding with multiple transformer layers and a binary classifier, where each transformer block consists of a multi-head attention followed by a multi-layer perceptron module, including an adjacent drop-out and normalization layer, respectively (Fig. 1C). These blocks iteratively modulate the peptide ion-derived tokens together with a concatenated classification (CLS) token, generating a set of values that is read out by a terminal single-layer perceptron module to give rise to a binary label prediction ranging from 0 to 1, indicating sample identity (Fig. 1C).

### Batch-robust classification and dose-dependent generalization by the ProFormer

**Fig. 2:**
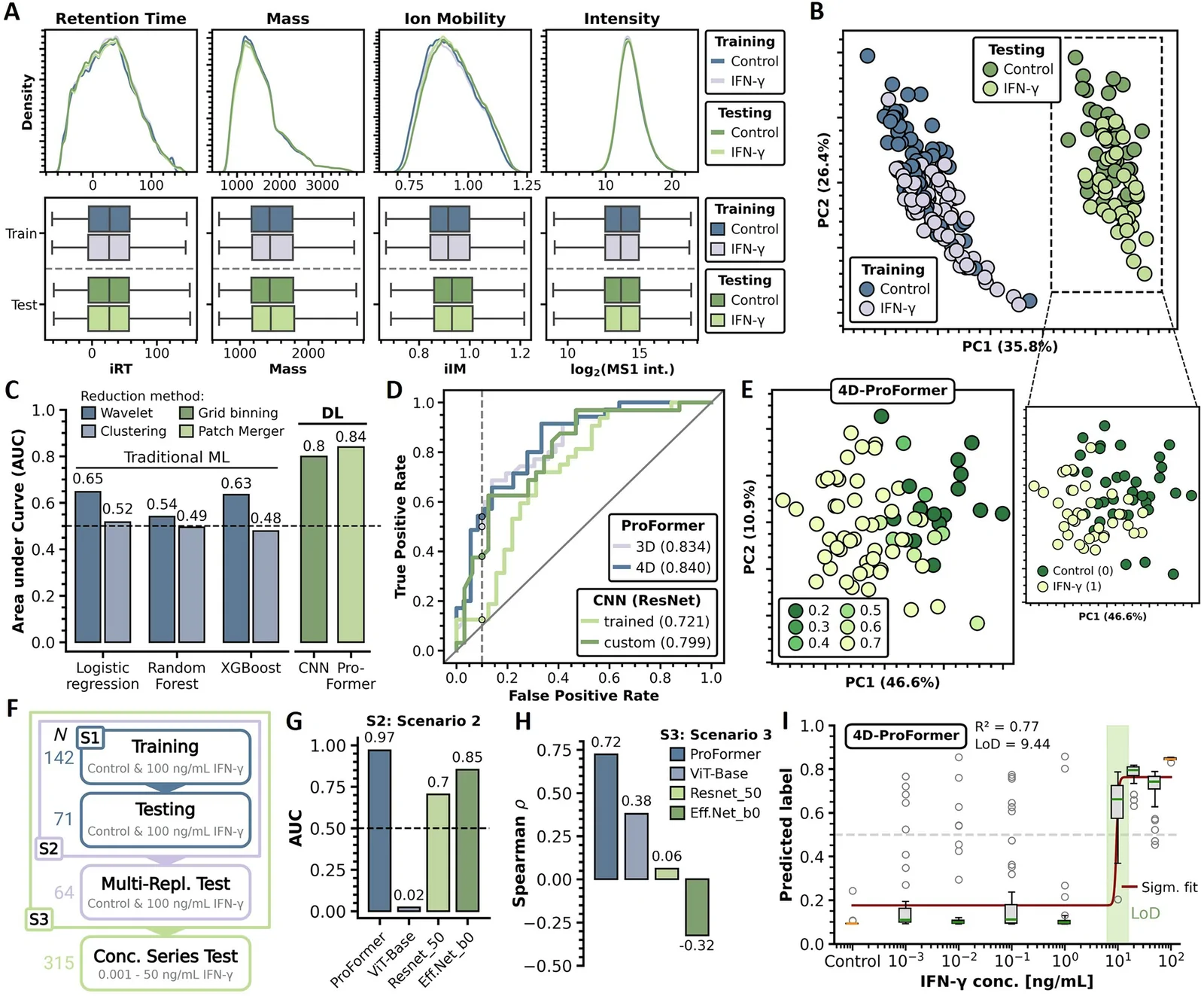
Benchmarking the ProFormer against alternative ML and DL pipelines. **A** Top: Kernel distribution of peptide ion features in training (blue: control, violet: IFN-γ-treated; *N* = 142) and test data (dark and light green; *N* = 71). Bottom: Box plots indicate the first and third quartiles, with whiskers showing 1.5-times the interquartile range and the median as the inner line. **B** Peptide ion PCA of training (*N* = 142) and test data (*N* = 71) color-coded as before. Explained variance ratios are indicated on both axes. **C** AUCs from traditional ML and DL algorithms on the test data. ML received features after Wavelet transform (dark-) or K-means clustering (light blue), image-based CNN (custom ResNet_v2_50) received data from grid binning (dark-), the ProFormer received output tokens of a patch merger trained in parallel (light green). The dashed line represents a random classification. **D** ROC over ProFormer models using four (blue) and three features (violet), and over pre- (green) and custom-trained ResNet_v2_50 (light green) models. Specified points show model sensitivity at 10% error. The diagonal represents a random classifier. AUCs are indicated in parentheses. **E** Left: Isolated peptide ion PCA of test data (control 0: dark green, IFN-γ 1: yellow). Right: 4D-ProFormer predictions. Explained variance ratios are indicated at both axes. **F** Summary of assessed test data scenarios. S1 (blue): evaluating measurement batch domains (A–E; *N* = 6), S2 (violet): evaluating biological domains (*N* = 64), S3 (green): evaluating IFN-γ effect sizes (*N* = 315). **G** AUCs of DL pipelines tested in S2. **H** S3: Spearman ρ correlation of predictions across IFN-γ concentrations. **I** S3: 4D-ProFormer predictions as boxplots. Boxes show the first to third quartile with the median as the colored central line (Orange: control and 100 ng/mL IFN-γ (*N* = 16 each), Green: seven intermediary concentrations not contained in the training set (*N* = 45 each)). Whiskers indicate 1.5-times the interquartile range, outliers are shown as circles. The red line shows a sigmoidal fit for the model predictions, with the inflection point marking the limit of detection (LoD, green). R^2^ is indicated at the top. Source data are provided as a Source Data file. Figure 2F [https://BioRender.com/myaxk3i] created in BioRender by Krull, KK (2026).

To critically assess the performance of the ProFormer, we employed this pipeline to discriminate peptide ion profiles from HeLa cell samples before and after treatment with Interferon gamma (IFN-γ), an extensively described cytokine that induces the expression of immune-modulatory proteins, however with only mild effects in non-immunogenic cells^15,16^. Therefore, we cultivated six HeLa cell cultures and treated three of them with IFN-γ for 24 hours. We then generated a training data set by injecting 100-ng treatment and control peptide samples in technical replicates on a timsTOF Pro mass spectrometer (*N* = 142 total injections), acquiring MS1 and MS2 information using a diaPASEF acquisition mode to minimize missing values between runs (Supplementary Fig. 1A). To evaluate resistance to model overfitting, we deliberately introduced a batch effect in the test set by performing temporally distant acquisitions. Precisely, we re-measured samples from the same cultures (*N* = 71 injections) six months apart, maintaining all other conditions constant, including the instrument and experimental settings. Of note, in its first instance, this setup cannot provide completely independent testing, but serves to demonstrate how hereafter tuned AI models handle technical domain shifts. To demonstrate the ProFormer concept, we used DIA-NN^17^ as an established tool to reliably extract MS1 features from raw LC/MS data, while simultaneously enabling MS2-based peptide identification to validate the ProFormer outputs. In this way, we achieved deep proteome coverage, i.e., high data dimensionality, identifying approximately 145,000 peptide ions per injection in the training set (test data: 135,000 peptide ions per injection) (Supplementary Fig. 1B). Among the identified peptide ions, we observed excellent data completeness, with almost half of them identified in every sample and a median of 5–6% missing values across injections in both sets (Supplementary Fig. 1C), thus ensuring data consistency for model training. Remarkably, fold changes of peptide ions between control and treated samples showed strong agreement across the training and test sets, showing a Pearson correlation of +0.784, while revealing the specific up-regulation of peptide ions associated with IFN-γ-responsive proteins, such as the tryptophanyl-tRNA synthetase WARS1 that exerts signaling functions upon IFN-y-stimulation^18,19^ (Supplementary Fig. 1D, E). Next, we masked the identity of all peptide ions to simulate undirected signal extraction from raw data and used the ion’s indexed retention time (iRT), indexed ion mobility (iIM), mass-over-charge (m/z), and intensity features (peak area) per sample from the software’s report tables, aiming to discriminate treated from control samples solely based on these peptide ion profiles. A global assessment of features between training and test data sets demonstrated their technical equality, showing close to identical distributions, while control and treatment conditions produced minor differences, likely resulting from the effect of the IFN-γ treatment (Fig. 2A). Yet, quantitative differences were indeed evident in the data sets, leading to the formation of two cohesive clusters in peptide ion-level principal component analysis (PCA) (Fig. 2B), which showed the anticipated separation of training and test data according to their time of acquisition. Importantly, division of treated and non-treated samples was preserved in both clusters, although the IFN-γ effect did not lead to a clear separation (Fig. 2B). Within each cluster, distinguishing treatment from control was complicated by our experimental approach using technical replicates to model measurement variability, which interfered with the underlying cellular changes. Hence, we designed a particularly difficult task with a focus on technical uncertainties and the simulation of time-distinct sample measurements, for which a model needs to generalize the classification problem by transferring learned relationships from one batch to another.

In this setting, we used the masked list of discrete peptide signals as input to a series of machine learning (ML) architectures, evaluating their performance in discriminating control from IFN-γ-treated samples. First, we explored the performance of traditional ML and tuned different models using logistic regression, random forest, and XGBoost algorithms on the training set in a five-fold cross-validation approach (hyperparameters in Supplementary Data 1), comparing their results based on the test data (summarized in Table 1). All three classifiers received data as tabular input, and we evaluated their combination with two deterministic methods for reducing the high dimensionality of peptide ion signals. Interestingly, we found that the wavelet transform consistently outperformed K-means clustering, achieving higher performance with all tested ML algorithms (Fig. 2C). Nevertheless, the best-performing logistic-regression model only achieved up to 64.7% area under the curve (AUC) accuracy, showing the limitations of traditional ML in resolving our established classification task. On the contrary, when we challenged a series of DL pipelines with the same objective (Supplementary Data 1), the majority of generated models demonstrated much higher accuracies than those obtained by ML models (Table 1), reaching the highest value of up to 84.0% AUC by the ProFormer (Fig. 2C). This finding not only highlights the capabilities of our pipeline, but it also justifies the use of deep learning for the application of peptide ion data in this work. In this context, classification accuracies illustrated in Fig. 2C derive from the best-performing DL models we obtained among various tested architectures shown in Table 1. In particular, we focused on previously used CNN pipelines that receive data after image conversion (Supplementary Fig. 2) and compared them to different implementations of transformer architectures. Specifically, this included our proposed tabular pipeline, which used three (3D-ProFormer: m/z, iRT, and intensity) or four peptide ion features (4D-ProFormer: additionally including iIM), and a vision transformer that is – like all image approaches – inherently restricted to three features. Among the tested CNN pipelines, the highest accuracy on the test data was achieved for a (pre-weighted and custom-trained) Residual Network (ResNet_v2_50) architecture, whose performance exceeded seven other CNN network types (Table 1), and which also demonstrated promise for proteomic data analysis by others previously^4^. However, although the ResNet_v2_50 achieved up to 79.9% AUC, we found that its performance was strongly dependent on the initial weighting before tuning, resulting in significantly higher accuracies with the custom-trained network than with the pre-trained network (72.1% AUC). As an important note, we also observed a strong discrepancy between results of the validation and test sets for this architecture, showing a drop in accuracy of around 20–28%-points, which was even more pronounced for other CNNs (22–57%-points) (Table 1). This finding calls into question the high accuracies previously reported for CNN architectures on non-independent data sets, suggesting that they may have been the result of overfitting, as these performances could not be reproduced when we evaluated them in this work on a semi-independent test set. Interestingly, we observed smaller differences between accuracies of the validation and test set for the vision transformer (6 – 23%), but found that the ProFormer pipeline was the only one achieving equivalent results on both data sets, while also demonstrating the highest AUCs on the test set for this classification task (3D-: 83.4%, 4D-ProFormer: 84.0%; Table 1). We thus conclude that our tabular approach of combining dimensional reduction by a patch merger together with subsequent transformer blocks substantially enhances model robustness to batch effects in proteomic data.

**Table 1:** Performances of various ML & DL models on an in-house IFN-γ dataset with temporally distant measurements

Input	Reduction method	Type	Algorithm	Network	Val. AUC	Test AUC	Discrepancy
Tabular	Wavelet transform	ML	Logistic regression	–	0.609	0.647	0.038
			Random Forest	–	0.485	0.534	0.049
			XGBoost	–	0.622	0.635	0.013
	K-means clustering	ML	Logistic regression	–	0.464	0.516	0.052
			Random Forest	–	0.535	0.494	−0.041
			XGBoost	–	0.508	0.478	−0.030
	Patch merger	DL	3D-ProFormer	–	0.787	0.834	0.047
			4D-ProFormer	–	0.824	0.840	0.016
Image	Grid binning	DL	Vit_base_patch16_224^12^ (Vision transformer)	Custom	0.993	0.767	−0.226
				Pre-trained	0.841	0.780	−0.061
			Resnet_v2_50^43^ (CNN)	Custom	1.0	0.799	−0.201
				Pre-trained	1.0	0.721	−0.279
			Resnet_v2_18^43^ (CNN)	Custom	1.0	0.750	−0.250
				Pre-trained	0.993	0.774	−0.219
			Resnet_v2_101^43^ (CNN)	Custom	0.994	0.562	−0.432
				Pre-trained	0.990	0.421	−0.569
			Tf_efficientnet_b0_ns^45^ (CNN)	Custom	1.0	0.710	−0.290
				Pre-trained	1.0	0.757	−0.243
			Tf_efficientnet_b1_ns^45^ (CNN)	Custom	0.964	0.527	−0.437
				Pre-trained	1.0	0.691	−0.309
			Densenet121 (CNN)^46^	Custom	1.0	0.503	−0.497
				Pre-trained	1.0	0.742	−0.258
			Inception_resnet_v2^47^ (CNN)	Custom	0.986	0.704	−0.282
				Pre-trained	1.0	0.614	−0.386

Next, we inspected label predictions of the ProFormer and the best-performing CNN architecture ResNet_v2_50 for samples of the test set and analyzed their respective receiver operating characteristics (ROC) (Fig. 2D). In particular, we found that the models had very similar ROC curve progressions, showing high sensitivity for IFN-γ-treated samples at moderate specificity. Still, models derived from the ProFormer pipeline revealed a tendency towards better sensitivities when aiming for high specificity, exemplified by an arbitrary label cutoff at 10% false positive rate (Fig. 2D). Hence, the model might be useful in scenarios where high confidence is more important than missing out on positive results. Focusing on the 4D-ProFormer model, we assigned predicted labels to the PCA analysis of the test dataset (true labels: 0 – control, 1 – IFN-γ-treated) and observed that predictions of distant samples were closer to their true label than for samples in the overlapping region (Fig. 2E). While this observation shows that high quantitative similarity complicates the discrimination of proteomic samples, it suggests that the model’s prediction is indeed determined by the sample’s intensity profile in control and IFN-γ-treatment rather than by technical covariates.

We established that previously used DL architectures struggle to discriminate highly similar proteomic profiles when confronted with domain shifts of batched sample measurements. In contrast, the ProFormer mitigated this problem and maintained high classification accuracies between the validation and test sets. To evaluate the ProFormer’s performance on other shift dimensions, we generated a second test set, this time derived from HeLa cell samples that were individually cultured in instances of 30,000 cells (*N* = 64) in a 96-well format, half of which were treated with 100 ng/mL IFN-γ (multi-replicate test). Compared to the previous experiment, the resulting data were thus shifted on unseen dimensions of varying biological passage, cultivation conditions, technical preparation and measurement, and were generated by a different operator (concept matrix in Supplementary Data 2). Each of these dimensions poses a highly challenging single-source domain generalization task. However, in this scenario, we were only interested in assessing the models’ ability to generalize across preparative and biological differences, and we therefore included the previous test set in the training material, reducing the effect of the time dimension by adding a second measurement batch domain (Fig. 2F, Scenario 2). Using this enriched training data set, we tuned the four previously best-performing architectures (Table 1: 4D-ProFormer, ViT-Base_Patch16_224, ResNet_v2_50 & EfficientNet_b0) and assessed their performance on the prepared multi-replicate test set. Remarkably, three out of four architectures maintained their discriminative power despite the application of distinct biological domains, achieving an accuracy > 50% (random choice), with the 4D-ProFormer reaching the highest AUC of around 97% (Fig. 2G). Only the Vision-Transformer (ViT-Base) performed markedly worse, while it was still able to resolve sample classes, as the near-zero AUC resulted from a systematic label reversal that detected an IFN-γ treatment as control and vice versa. The other three models preserved label order, but their prediction scores differed only marginally between sample classes (Source data), pointing to uncertainty in their decisions. Although these results suggest that shifting data along unseen dimensions jeopardizes model robustness, the superior predictive performance of the ProFormer indicates its high potential for domain generalization. To verify this, we extended our assessment of DL architectures to a multi-source domain generalization task. In this third scenario, we confronted models with dynamic effect sizes of the IFN-γ treatment absent from the training material. We implemented this by a concentration series experiment, again culturing HeLa cells in 30,000-cell instances (*N* = 315) and treating them with seven different IFN-γ concentrations ranging from 0.001 to 50 ng/mL (*N* = 45 biological replicates per dose), reflecting largely different cellular responses to the same treatment. To provide additional examples of preparative and biological domains, we supplemented our training set with half of the multi-replicate test set (*N* = 32) (Fig. 2F, Scenario 3). The remaining, biologically distinct samples served as test data together with samples from different IFN-γ concentrations, thus presenting unseen domains along known dimensions to models from the same four architectures (Supplementary Data 2). For model evaluation, we anticipated that at lower IFN-γ concentrations, its effect on the cells would gradually diminish, ultimately resulting in a proteome profile comparable to that of untreated control cells. Hence, a generalized model would have a (non-linear) positive correlation between treatment concentration and its predictive output, leading to a score closer to 0 (control) for lower doses and a score approaching 1 (IFN-γ) for doses closer to 100 ng/mL. Notably, only the ProFormer satisfied the criterion of positive monotonicity, demonstrating a Spearman correlation of +0.72, whereas other models showed little to no correlation (ViT-Base and ResNet_v2_50) or even a negative trend (EfficientNet_b0) (Fig. 2H), inconsistent with the biological rationale of this experiment. Next, we tested if models could recognize the effect of the cytokine at a much lower concentration than in the training set (sensitivity), or predict a negative result when the concentration falls under a specific threshold, preventing false positives (specificity). To verify this, we used a sigmoidal fit to the predictions of each model as a function of IFN-γ concentration. We showed that the ProFormer indeed followed the desired dose-response relationship, with a coefficient of determination of R^2^ = 0.77 (Fig. 2I). In contrast, predictions of other models showed a much higher deviation from this fit as a result of low predictive precision (EfficientNet_b0), low sensitivity (ViT-Base) or low specificity (ResNet_v2_50) (Supplementary Fig. 2). Consequently, the ProFormer model not only offered highly accurate classification of biologically independent control and IFN-γ-treated samples, but also recognized treatment effects down to a concentration of 9.44 ng/mL, a dose about one order of magnitude below those used during training (Fig. 2I, limit of detection (LoD)). Therefore, our data demonstrate that the ProFormer generalizes beyond its binary training objective, accurately determining an IFN-γ response, despite varying effect sizes. We thus conclude that the ProFormer combines superior performance with robust generalization in proteomic data classification, outperforming previously used image-based data representations in single- and multi-source domain generalization problems.

### A learnable patch merger preserves information content while reducing peptide dimensionality

**Fig. 3:**
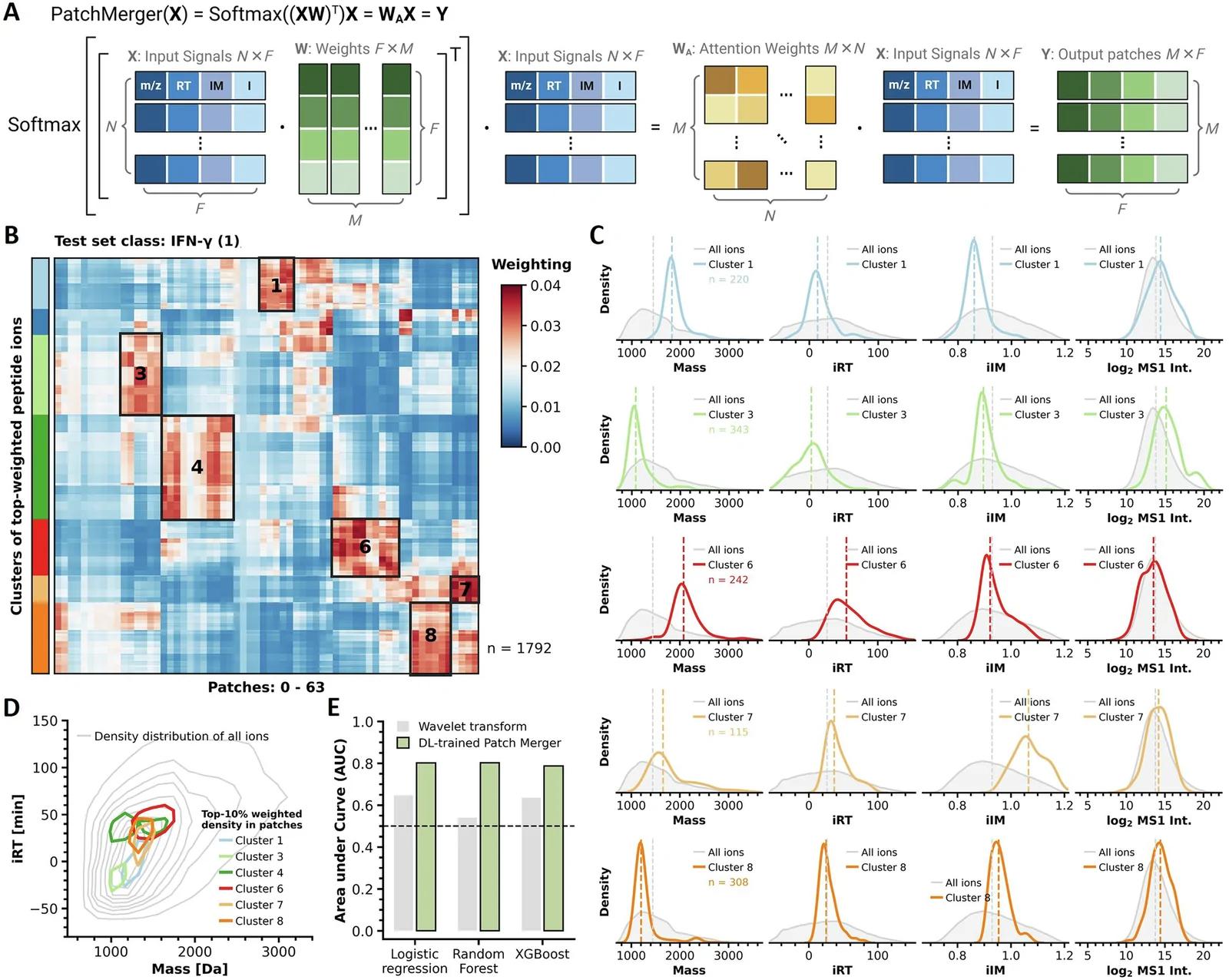
Explaining data aggregation by the patch merger in the context of peptide ions. **A** Illustration of matrix calculations inside the patch merger. An attention weight matrix **W**_**A**_ (yellow) is generated from the dot product of the input signal matrix **X** (blue) and internal weights **W** (green) after modulation by a Softmax function. Dot product of **W**_**A**_ with **X** reduces the initial dimensionality *N* to a number of output patches *M* while persevering features *F*. **B** Heatmap representation of attention weight matrix **W**_**A**_ in the patch merger clustered by Euclidean distance for an arbitrary test set sample in the data set from Fig. 2. Only top-weighted peptide ions per output patch across a set of eight test samples are shown (resulting in *n* = 1792). Color bar on the left indicates eight signal clusters with high attention weights. **C** Kernel density distribution of peptide ion features from a selection of clusters in B (same color-coding) compared to the respective feature distribution of all peptide ions (gray). Dashed lines highlight the distributions’ median. **D** Mass-to-iRT regions with top-10% highest weighted density of patch clusters in B (same color-coding) in front of a density distribution of all peptide ions (gray). **E** AUCs achieved by three traditional ML algorithms on test set data from Fig. 2 when trained on output patches of the Patch merger tuned in the ProFormer pipeline (green) compared to the output of a Wavelet transform function (gray; taken from 2 C). Dashed line represents a random classification. Source data are provided as a Source Data file. Figure 3A [https://BioRender.com/tsej7o1] created in BioRender by Krull, KK (2026).

An important aspect of our ProFormer pipeline is its compatibility with tables as input for its transformer architecture. During our analysis of various DL pipelines, we noticed that the transformer already performed particularly well on proteomic data using images as input, approaching the accuracies observed for CNN architectures (vision transformer: 78.0%, ResNet_v2_50: 79.9%; Table 1). Yet, only in combination with tabular data do the resulting models stabilize their performance to overcome the different measurement batch domains between the training and test sets, while this was achieved using the output of a patch merger. This module uses a learnable weight matrix to process individual peptide ion signals, comparing them to each of its weights by calculating their dot product and aggregating signals based on their similarity (Fig. 3A). As the model is trained, these weights adjust, allowing the input signals to be reflected in the most relevant way to the classification task, optimized against the model’s loss function. Since the patch merger operates within its given feature space, weights act as a learnable reference point to the peptides’ physical m/z-ratio, retention time, ion mobility, and intensity. Thus, each output patch constitutes a weighted aggregation of the input features localized in spatial relationship to a dynamically determined reference point. To demonstrate this in the context of our IFN-γ experiment (Scenario 1), we analyzed the generated attention weights, i.e., patch compositions (Supplementary Data 3), for a number of randomly selected test set samples (*N* = 8), investigating top-weighted peptide ion signals per patch. Clustering these attention weights across signals and patches revealed the formation of equivalent clusters in all samples irrespective of their sample class (Supplementary Fig. 4A), suggesting that a similar selection of peptide ions is highly relevant for the generation of the respective output tokens. For one representative test sample of class 1 (IFN-γ-treated), this led to a number of eight mayor clusters (Fig. 3B), whose underlying peptide ions showed an apparent focus on distinct regions in the feature space, rather than representing a random selection of signals from all peptide ions (Fig. 3C). This finding highlights that patches, although composed of all peptide ions, weigh distinct collections of signals differently, which are grouped based on regional similarities. This is further underpinned by the observation that weights of entire patches from different clusters aggregate in distinct regions of the m/z-to-retention time plane (Fig. 3D and Supplementary Fig. 4B), suggesting a division of the available feature space among them. Notably, in contrast to the binning approach used for the generation of data images, the presented patch merger divides the feature space in a dynamic fashion, not limited to a rigid grid pattern, with variable borders and numbers. Moreover, the presence of all peptide ions in each patch allows focusing on multiple regions at the same time, as exemplified by Cluster 4 in Fig. 3D, while the presence of smaller attention areas is a likely consideration. Remarkably, tuning the patch merger in the ProFormer pipeline (Scenario 1) and using its produced dimensions as input to train classical ML substantially improved the obtained classification accuracy for all tested algorithms, achieving up to 80.3% AUC by a random forest (Fig. 3E). While this result did still not reach up the level of the ProFormer (84.0% AUC, Table 1), it yet demonstrates that the patch merger produces highly discriminative dimensions, retaining a high information content, which can also be effectively used by other ML algorithms, prevailing over statistical approaches tested in the context of high-dimensional peptide data (cf. Fig.2C).

Collectively, these data demonstrate that the ProFormer is a valuable alternative to ML algorithms and previously applied DL image pipelines, enabling accurate classification of proteomic samples based on their MS1 peptide ion profile. This has been achieved by the combination of a transformer architecture with a patch merger, which provides an advanced framework for reducing dimensionality in tabular peptide ion data and allows the implementation of additional peptide ion features during data acquisition, including ion mobility separation.

### Enabling peptide-level classification of single cells

**Fig. 4:**
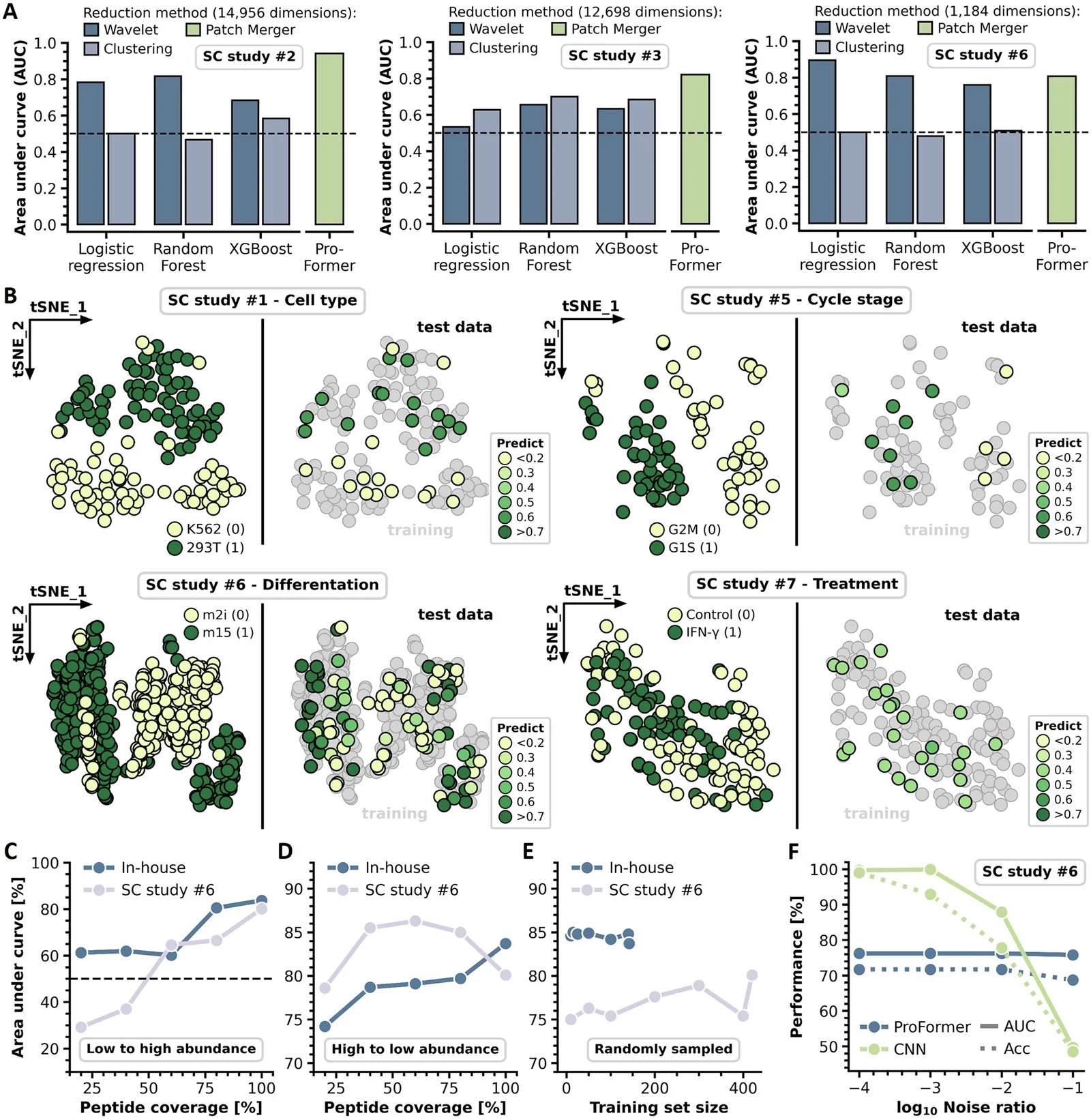
Exploration of data attributes determining the performance of ProFormer models. **A** AUC accuracies achieved by three traditional ML algorithms (blues) compared to the ProFormer on three selected, recently published single-cell (SC) data sets taken from Table 2 (left: SC study #2; middle: SC study #3; right: SC study #6). Color shades indicate the approach to reduce data dimensionality: ML methods received peptide ion features after Wavelet transform (dark-) or K-means clustering (light blue), the ProFormer received output tokens of a patch merger trained in parallel. Dimensionality per data set indicated on the top. Dashed line represent a random classification. **B** Peptide ion-level tSNE analysis of four SC studies taken from Table 2 (top left: SC study #1; top right: SC study #5; bottom left: SC study #6; bottom right: SC study #7) with true labels on the left and test set predictions by the ProFormer on the right (labels: 0.2–0.7). Subject of classification indicated on the top. Samples used for model training shown in light gray. **C** AUC accuracies of the ProFormer trained on different peptide ion subsets that included the lowest abundant 20, 40, 60, 80% and all detected peptide ions in the bulk IFN-γ experiment (blue) and in SC study #6 (violet, from Table 2), respectively. **D** Same as (**C**) but for the highest abundant 20%, 40%, 60%, 80% and all peptide ions. **E** Same as (**C**) and (**D**) but for varying training set sizes (randomly sampled). **F** Classification performance (AUC: solid; Accuracy: dotted line) of the ProFormer (blue) and a CNN image-pipeline (ResNet_v2_50, green) on SC study #6 from Table 2 with varying ratios of Gaussian noise applied to each of the test set features. Source data are provided as a Source Data file.

Having constructed and successfully tested the ProFormer pipeline for LC-MS data of conventional bulk proteome samples, we aimed to extend its applicability to the classification of more diverse sample types and testing conditions. In contrast to traditional proteome analysis, in which experiments usually entail only a few replicates, the emerging field of single-cell proteomics (SCP) and the clinical analysis of blood plasma or serum samples offer great potential for our approach. In particular, they both require large numbers of samples to achieve robust statistics, making them ideal for providing DL pipelines with sufficient training material. In a first step, we focused on the implementation of single-cell data. Therefore, we collected raw files of several recently published studies that have investigated proteome differences between cell types or states (Table 2), and we divided each collection into a training and test set. We re-analyzed both sets individually using DIA-NN^17^ and MaxQuant^20^ for DIA and DDA data, respectively, and we extracted the resulting MS2-informed peptide ion features per cell from the report tables for model training (retention time, m/z, intensity, and, if measured, ion mobility). Since intensity normalization is essential for such large data sets, we tested different strategies as input to ProFormer, including a median-normalization based on peptide ions without missing values in the training set, followed by data standardization (Z-score or robust Z-score). Consequently, we selected the method with the highest accuracy during model tuning, i.e., on the validation set (Supplementary Data 4), and evaluated the performance of ProFormer models on the respective test set for each single-cell classification task (shown in Table 2). For a selection of these studies, we showed that the obtained accuracies greatly exceeded the results achieved by traditional ML algorithms when data dimensionality was high (SC study #2 and #3, Fig. 4A), recapitulating the benefit of the ProFormer. However, in cases where dimensionality was lower (SC study #6), a combination of Wavelet transform and subsequent logistic regression model outperformed the ProFormer (Fig. 4A). This finding is likely to be explained by the limited ability of classical approaches to effectively reduce dimensionality with a growing number of dimensions. In turn, when dimensionality is moderate, traditional ML might still constitute a powerful alternative. Yet, this remains an exception for contemporary proteomic data, which usually entails 10 – 100 thousands of peptide ion signals, even in SC proteomics. In contrast, the ProFormer showed broad applicability to diverse classification tasks of single cells, achieving particularly high accuracies for different cell types (study #1^21^ and #2^22^, Table 2) and cell cycle stages (SC study #4 and #5^23^), reaching AUCs from 94.2 – 100%, indicating its ability to discriminate highly divergent samples. Moreover, the ProFormer accomplished 82.1% for the classification of migrating among stationary cells (SC study #3^22^) and 80.8% for pluripotent states of mouse embryonic stem cells (mESC) (SC study #6^24^). This is still a highly favorable outcome, considering that it is more challenging to distinguish altered states of the same cell type, instead of contrasting different cell types.

**Table 2:** Performances of the ProFormer for classifications in label-free single-cell proteome studies

No.	Ref.	Resource	Features	Samples: train / test	Peptide ions per sample	Experiment	AUC test set	Sensitivity at 5% error
#1	^21^	Hu M et al., 2023	3D	135 (105 / 30)	763	HEK-293T vs. K562 cells	1.00	1.00
#2	^22^	Wang Y et al., 2024	4D	81 (65 / 16)	14,956	A549 vs. HeLa cells	0.942	0.714
#3			4D	89 (70 / 19)	12,698	Migrating vs. stationary cells	0.821	0.571
#4	^23^	Brunner A et al., 2022	4D	127 (100 / 27)	3,485	HeLa cell cycle G1 vs. G2	0.943	0.455
#5			4D	86 (70 / 16)	6,517	G1S vs. G2M	1.00	1.00
#6	^24^	Petrosius V et al., 2023	3D	519 (420 / 99)	1,184	mESC populations	0.808	0.469
#7	^16^	Krull KK et al., 2024	4D	143 (115 / 28)	1,477	U-2 OS cells +/- IFN-γ	0.736	0.0

In an even harder case to discriminate untreated osteosarcoma cells (U-2 OS) from those exposed to IFN-γ (SC study #7^16^), the accuracy still reached 73.6%, despite the subtle effect of this treatment. Notably, the number of detected peptide ions differed considerably between data sets (763–14,956, Table 2), however the low number of peptide ions in SC study #1 still sufficed to completely discriminate HEK-293T from K562 cells (Table 2). The number of peptide ions in the single-cell IFN-γ treatment was slightly higher (1,477, SC study #7), but this was approximately 100x lower than in our bulk experiment (around 140,000) (Supplementary Fig. 1B), providing a potential explanation for the almost 10%-lower AUC value of 0.736 (Table 2) compared to 0.840 (Fig. 2C). In this context, we anticipate that the difficulty of the classification task, i.e. its achievable accuracy, is largely determined by the quantitative similarity of labels that are to be distinguished. To illustrate this point, we clustered peptide ion data by t-distributed stochastic neighbor embedding (tSNE) in a selection of single-cell studies involving different cell types, cell cycle stages, the mESC differentiation states and the IFN-γ treatment, and we annotated the test set labels predicted by the ProFormer (Fig. 4B). This analysis revealed that predictions aligned with their true labels in scenarios where differences in cell populations were most pronounced (SC study #1 – cell types, and SC study #5 – cycle stages), but displayed values closer to 0.5 in the overlapping regions that are characterized by more subtle biological differences (SC study #6 – mESC differentiation, and SC study #7 – IFN-γ treatment). Consequently, these latter conditions are harder to disentangle, and they are likely to occur when such a model is developed for realistic settings, in which complex biological signatures need to be resolved against a background of substantial biological heterogeneity and technical variation. This serves as an explanation for why ML classifiers are required to classify proteomic data and cannot be performed by reduction algorithms like t-SNE or UMAP alone. Therefore, we next exemplified ways to improve the ProFormer’s classification accuracy in cases of small quantitative differences between the experimental conditions and highlighted the effect of training set size and data dimensionality, i.e. number of detected peptide ions. Specifically, we focused on the analysis of our in-house generated IFN-γ data set (Fig. 2, Scenario 1) and the study on single mESCs (SC study #6, Table 2), since both achieved high AUC values > 80% despite dealing with particularly difficult tasks. Using these data, we trained the ProFormer with a subset of the available peptide ions, gradually increasing the considered intensity range from low to high abundance and vice versa (Fig. 4C, D). Models only trained on low-abundance peptide ions performed worse than those also including higher-abundance peptide ions (Fig. 4C). Only considering the 20%-highest abundance peptide ions already reached high accuracies in both data sets, but including lower-abundance peptide ions improved the performance gradually for the in-house bulk study (Fig. 4D). Interestingly, we observed a similar trend in case of single-cell data, but the performance reached a maximum with the 60%-highest abundance peptide ions, improving the classification accuracy to around 86% (+5% points) compared to using the entire data set (Fig. 4D). Since low-abundance peptide ions usually suffer from higher missing values and quantitative inconsistencies in proteomic data (especially in the context of single cells), maintaining data quality is crucial, where removing these signals can help to increase model performance. Next, we demonstrated the effect of the training set size on the model’s performance, showing highly consistent AUC values, even with only a fraction of samples (Fig. 4E). Remarkably, training the model only on ten samples, the ProFormer already achieved accuracies of around 85% for the in-house data set and 75% in case of the single-cell data, while larger training set sizes in the latter gradually improved the performance to the reported 80.8% AUC using all available samples (*N* = 420), as expected (Fig. 4E). We did not observe a similar trend for the in-house data, yet it consists of considerably fewer samples, and we therefore anticipate to have seen the same effect with a larger training set size. At the same time, including more samples during model tuning also resulted in a clearer separation of predicted labels for the test set (Supplementary Fig. 5A), suggesting more definite decisions, thus higher robustness to small differences. Notably, single-cell studies from Table 2 were not specifically designed by the authors to contain independent data sets and thus only reflect one technical domain, while each of the cells can be considered biologically distinct. Hence, we demonstrated the high generalization capability of the ProFormer by artificially introducing Gaussian noise to the features of the test data of SC study #6 before re-evaluating the model’s performance, where the ProFormer maintained its classification accuracies, proving resilience to up to 10% noise (Fig. 4F). This observation strongly contrasted to the results of ResNet_v2_50 CNN, which revealed a gradual loss of its ability to discriminate the sample classes with higher noise ratio, demonstrating a random classification at 10% noise, despite having achieved 100% AUC without noise. This aligns with our findings from the previous analyses, where this network could not maintain its high classification accuracy from the validation data for an inherently different test set (cf. Table 1). For completeness, we also introduced Gaussian noise to the test set of the in-house IFN-γ experiment (Scenario 1) and showed that, already with a small noise ratio of only 0.01%, the CNN’s performance dropped from 79.9% without noise to under 60% AUC, whereas the ProFormer’s results remained unaffected (Supplementary Fig. 5B).

We conclude that the ProFormer is a versatile tool that can be used for the classification of single cells, achieving up to 100% accuracy when contrasting cells with divergent proteome composition (different cell types or cell cycle stages), and reaching accuracies of up to 85% for closely related cells. The difficulty in discriminating cells is primarily influenced by their biological similarity, while depth and quality of single-cell proteomic data have not reached the same standard known from bulk analysis yet. Hence, we emphasize that ensuring data quality by excluding low-intensity peptide ions and covering the diversity of these biologically heterogeneous populations by increasing dataset sizes are both valid strategies to enhance model performance. Still, the observation that accurate predictions can be achieved with a relatively small number of training samples renders the ProFormer applicable even for studies with limited sample availability. In this context, the ProFormer’s ability to withstand high ratios of data noise verifies our early findings that this pipeline offers high potential for model transferability, and this might prove valuable, for instance, when classifying biological outliers not captured by the initial training material.

### DL-driven plasma proteome diagnostics for effective determination of patient disease states

**Fig. 5:**
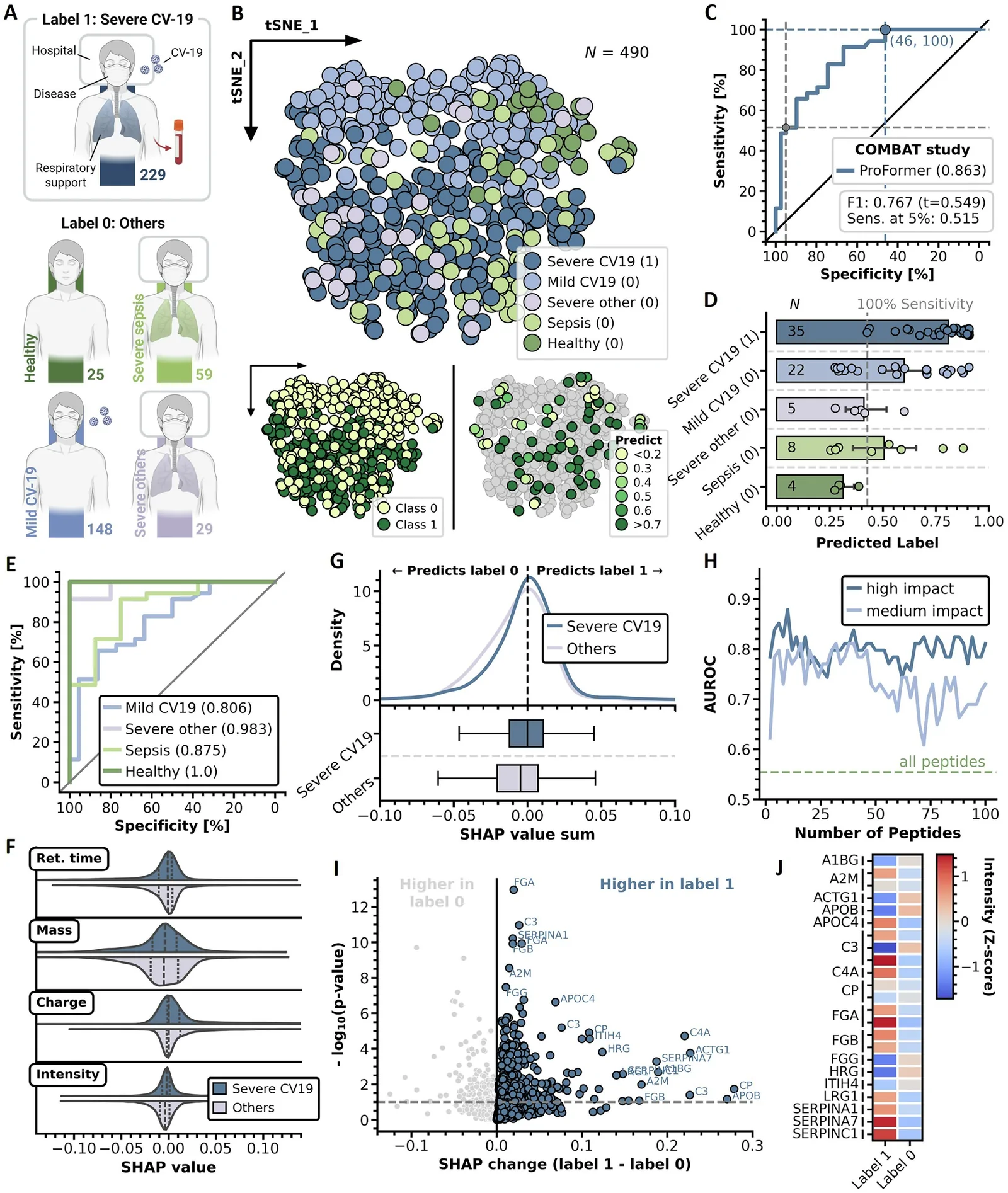
Plasma proteome classification of COVID-19 patients enrolled in the COMBAT study. **A** Disease conditions among COMBAT patients^25^ to binary classify 1: severe COVID-19 against 0: other conditions. Label 0 includes healthy volunteers (dark), severe sepsis (light green), other severe respiratory infections (violet), and mild COVID-19 (blue). Patient numbers per condition are indicated. **B** Peptide ion tSNE analysis of plasma proteome profiles (*N* = 490) color-coded as before (top). True labels on bottom left, predictions on bottom right. **C** ROC analysis of the 4D-ProFormer recognizing severe COVID-19. Dashed lines indicate model specificity at 100% recall (blue) and sensitivity at 5% error (gray). Solid line represents a random classification. AUC is shown in parentheses. F1 score at the tuned label threshold t is indicated. **D** Average predicted label per disease (color-coding as before; test samples N indicated). Error bars shown as mean ± standard deviation (SD) with individual predictions indicated. Dashed line shows label cutoff corresponding to 100% recall. **E** ROC analysis in the isolated discrimination of control conditions to severe Covid-19. AUCs are shown in parentheses. **F** SHAP values across peptide ion features in class 0: Others (violet) and 1: Severe COVID-19 (blue). Dashed lines indicate the median, first and third quartiles. **G** Average SHAP sum per peptide ion in the respective class (color as before; *n* = 4787). Positive values refer to label 1 (severe Covid-19), negative values refer to label 0 (Others). The dashed line shows a SHAP sum of 0. Top: kernel density distribution. Bottom: boxplots from the first to third quartile with the median as the central line. Whiskers specify 1.5-times the interquartile range. **H** Test AUCs achieved by a random forest classifier trained on a number of peptide pairs selected by their SHAP sum (high impact (dark): highest positive/negative values in G; medium impact (light blue): values around 0). Green line shows the AUC of a model trained on all peptide ions. **I** Scatter plot of average SHAP sum differences between test samples tested by two-sided Student’s t-statistics. **J** Z-scored intensity for highly significant peptide ions from H in classes 0 and 1. Source data are provided as a Source Data file. Figure 5A [https://BioRender.com/esrl7c1] created in BioRender by Krull, KK (2026).

One of the greatest perspectives for DL-assisted proteome classification is its application as a diagnostic tool to indicate clinical conditions of patient-derived samples. Since this is preferably done on blood plasma as an accessible and abundantly used clinical entity, we evaluated the potential of our ProFormer pipeline for plasma proteome data. The analysis of such data is notoriously difficult, as (subtle) disease-driven proteome differences need to be distinguished from (potentially large) inter-individual variations. In addition, the plasma proteome is dominated by a small number of high-abundance proteins that hamper the identification of disease-associated peptides. Hence, the classification of these samples is, among the tested scenarios in this work, likely to be most challenging to achieve, yet has the greatest potential for real-world implementation. We therefore sought to create a classifier based on the recently published COMBAT (COvid-19 Multi-omics Blood ATlas) consortium study^25^, which comprised multiple omics layers, including the plasma proteome analysis of 490 participants, exploring differences between patients who suffered from severe Covid-19 after SARS-CoV-2 (severe acute respiratory syndrome coronavirus 2) infection and healthy volunteers. Importantly, this study also involved other disease conditions, such as mild Covid-19, severe influenza with and without secondary pulmonary Aspergillus infection, and severe sepsis caused by infection other than SARS-CoV-2. Here we focused on the evaluation of the ProFormer to establish a binary classification that distinguishes the severe COVID-19 condition from other disease conditions and from healthy individuals (Fig. 5A). Consequently, we re-analyzed the data in MaxQuant, identifying on average 1,336 peptide ions per sample, and used their detected MS1 features per sample as input for the ProFormer. Analyzing the intensity feature, we observed two cohesive clusters in a tSNE analysis, reflecting the degree of COVID-19 disease (mild and severe), but this was interspersed with samples from sepsis and other severe infections, which could not be distinguished as separate entities (Fig. 5B). Since we aimed to achieve high recall (sensitivity) for severe COVID-19 while minimizing false-negative outcomes, we modified the loss function of the ProFormer, assigning a stronger penalty to the misclassification of all non-severe COVID-19 samples during model tuning. Training the ProFormer with three peptide ion features (m/z, RT and intensity) resulted in an AUC accuracy of 72.7% on the validation set (test: 70.7%, Supplementary Fig. 6A). However, dividing the m/z-feature into its mass and charge components dramatically improved the performance to 81.3%, which reached a remarkable AUC of 86.3% on the test set (F1 score: 76.7%) (Fig. 5C). Setting the prediction cutoff slightly below 0.5, the resulting model achieved 100% sensitivity, while ruling out the disease in 46% of cases (true-negative rate, i.e. specificity) (Fig. 5C, D), demonstrating its potential when used a diagnostic tool. In this way, the model correctly recognized samples of healthy patients and of those with other severe infections as negative, but indicated some patients as false-positive that suffered from sepsis or mild COVID-19 (Fig. 5D). Nevertheless, in the isolated discrimination of serve COVID-19 to other disease conditions, this still achieved 80.6, 87.5, and 98.3% AUC accuracy for mild progression, sepsis, and other severe infections, respectively (Fig. 5E).

### Decisions of ProFormer-derived models are explainable at single-peptide resolution

In our effort to develop a classifier that has the ability to utilize a wide range of proteome sample types, including those collected at clinical facilities, it is crucial to improve our understanding of the model, explain its decisions, and increase confidence in its predictions. Therefore, we investigated the plasma test dataset using the SHAP (SHapley Additive exPlanation) value system^26^, which assigns an importance metric to each feature of a signal. This showed that all implemented features (RT, mass, charge, and intensity) significantly contributed to the output of the ProFormer model (Fig. 5F). The “mass” feature appeared to have the highest impact, which may be explained by the great precision with which this attribute is measured in the MS, potentially leading to high reliability for the model. To evaluate the contribution of individual peptide ions and to find denominators that majorly determine the prediction process, we summed feature SHAP values, resulting in a cumulative score per peptide ion signal. From this, we calculated the average SHAP sums across the respective sample class, where a positive value predicts the label 1 (severe Covid-19) and a negative value predicts the label 0 (all other disease conditions) (Fig. 5G). As expected, peptide ions identified in samples from the severe COVID-19 condition had a tendency towards higher SHAP sums, whereas peptide ions from other conditions showed overall lower values (Fig. 5G). However, both classes comprised negative and positive contributions, reflecting the complexity of the prediction process, in which the aggregated impact of all peptide ions results in the predicted label of a test sample. To evaluate the predictive power contained within these data, we selected the top-contributing peptide ions in each class and used them to train a random forest, a simpler ML classifier. This resulted in a prediction with equivalent accuracy to the DL model (approx. 80 – 85%), while selection of medium-impact peptide ions, i.e., SHAP sum around 0, resulted in overall lower accuracies, and using all peptide ions only achieved around 55% AUC (Fig. 5H). This constitutes an important finding, as it shows not only that the underlying classification task can be solved by the targeted analysis of a diligent selection of peptide ions, but also that the ProFormer is able to identify them among a large number of signals. Thereby, we demonstrated that SHAP values can serve to explain the foundation of the ProFormer’s predictions. Subsequently, we sought to link these results to inherent biological alterations between the label classes. Specifically, we explored peptide ions that showed high SHAP values in label 1, but low SHAP values in samples of label 0, and we performed a two-sided t-test on these signals to determine the significance of this difference. At this point, we unmasked the peptide identity as determined from MS2 data (Fig. 5I). Among peptide ions with the highest significant positive differences (p-value > 0.1), we found several that originate from proteins involved in shared biological processes, such as the complement system proteins C3 and C4A, and proteins acting in coagulation, including SERPINC1 (Antithrombin III) and the three fibrinogen proteins FGA, FGB and FGG (Fig. 5I). Importantly, these peptide ions showed considerable dissimilarities between patients of severe Covid-19 (label 1) and other disease conditions (label 0) in the underlying proteomic data (Fig. 5J). Notably, this closely reflects the observation reported by the authors of the COMBAT study from which these data were derived: they performed a conventional differential expression analysis at the protein level and observed an activated complement system and markers of tissue injury associated with severe COVID-19^25^. As an interesting addition, SHAP values revealed three peptides of the complement protein C3 as particularly important for the predictions of the ProFormer (Fig. 5I), among which one peptide was inversely regulated in the proteomic data (Fig. 5J). This peptide (567-592) marks the beginning of the C3-beta-c fragment of the C3 protein (Supplementary Fig. 6B), suggesting its proteolytic cleavage through complement activation, which would have gone unnoticed at the protein level. Hence, SHAP values have provided insights into the inner workings of the ProFormer model, and identified peptide ion signals could be linked to the underlying biology at stake, serving as a cornerstone towards making DL models on proteomic data explainable.

In conclusion, we have successfully implemented the ProFormer pipeline for tuning a classification model on plasma proteome samples, distinguishing a severe COVID-19 infection from several other disease conditions with high accuracy based on MS1 data. In this context, we have emphasized the importance of methods that facilitate the interpretability of DL approaches and presented SHAP values as a valuable resource to elaborate the rationale of ProFormer-derived models. Using this metric, we have shown that peptide ion signals deemed important for the ProFormer can achieve similar performance when applied to simpler ML models, validating the utility of these signals to discriminate the defined sample classes. Finally, our observation that peptide ions with close attention are indeed related to biological differences and not technical bias creates confidence in the decision-making of the ProFormer and encourages the use of explainable AI for the classification of proteome samples.

## Discussion

We here introduced the ProFormer, a transformer-based DL pipeline that classifies samples according to their multi-dimensional peptide ion profiles obtained from proteomic data. Conventional proteome analysis relies on the statistical comparison of protein abundances inferred from peptide identifications obtained through MS2 spectra. In contrast, the ProFormer utilizes peptide ion information obtained at the MS1 level without requiring peptide or protein identity. This information, including retention time, mass-to-charge, and intensity, is provided in a tabular input format to the ProFormer and can be extended to any additional features, such as ion mobility. Classifying samples using MS1 information unlocks significant advantages, as it enables higher processing speed and interpretability for proteomic data, increasing the translational prospect of pipelines for plasma and single-cell proteome analysis. There are already considerable advances in the high-throughput measurement of proteomic samples^27^, but subsequent interpretation often depends on lengthy processing and statistical operations, limiting application in areas requiring rapid decision-making, such as in clinical settings. Herein, the ProFormer might prove particularly valuable, providing decision-makers with accurate and rapid data interpretation. This could serve as a cornerstone for building future pipelines, projected to broaden the proteomic field beyond approaches focused on condition-specific protein expression. As a valuable addition, the ProFormer enables the annotation of cellular characteristics in single-cell proteomic data, leveraging opportunities for data analysis in this emerging field.

In contrast to the ProFormer, previously developed classifiers discriminate samples based on peptide or protein abundances obtained through LC-MS/MS data processing using established proteomic software. This has been used e.g., to prepare curated peptide sets for targeted validation^28–31^, or to complement conventional statistics with AI-derived importance metrics^32^. Still, abundance classifiers are typically limited to isolated experiments, as their generalizability is impaired by profound sample- and experiment-dependent processing biases. Among others, this is because technical variation can result in different sets of identified fragment ions across samples, causing differences in (MS2-based) peptide quantification and missing values^33^. In turn, sample-specific detection of peptides affects the identification and quantification of protein groups. To enable accurate quantitative comparison within one experiment, proteomic software thus harmonizes the set of quantified fragments or peptides across samples^34,35^. However, this complicates cross-experiment comparisons, as the same peptide or protein may be quantified based on different fragment or peptide sets, respectively. Pipelines using abundance classifiers would therefore need to follow a computationally heavy transductive approach, where new samples or experiments are analyzed along with the initial training material, or perform post-processing batch correction, in both cases changing the underlying data every time new data is included. Alternatively, we reason that direct use of MS1 data provides greater comparability across experiments and laboratories, facilitating the development of AI classification models with broad generalizability.

In earlier efforts, this has been approached by converting the MS1 features of proteomic samples into an image-like heatmap followed by classification through deep CNNs^4–8^, allowing search-free raw data analysis. However, as J. Cadow et al. already stated^4^, this can lead to a loss of information when aggregating biologically unrelated signals within spatial proximity (i.e., representing peptides that originate from different proteins). Rigid bin borders can further split signals into neighboring bins, confounding the resulting image and making it prone to data fluctuation. It thus remains unclear whether a downstream classifier would learn biologically relevant information or artefacts of the aforementioned issues. In this context, CNNs have proven to learn features of natural images, such as edges and corners^36^, but they are not present in MS data-derived synthetic images. It is therefore questionable whether the inductive bias of CNNs is at all suitable for MS data. Under these circumstances, we suspect that deep CNN architectures bear a high risk of overfitting, i.e., failing to generalize on the classification task. In contrast to CNNs, the ProFormer approach seeks to preserve the information content of individual signals, leveraging MS1 peaks as input for the classification, instead of training on synthetic images. Specifically, we adopted a patch merger that dynamically weighs and aggregates discrete MS1 peaks in a learnable fashion, which we anticipate will more faithfully represent high-dimensional proteomic data. In fact, we demonstrated that the patch merger produces dimensions that focus on self-determined regions in multi-dimensional feature space that are not limited to shapes or sizes, while allowing combination of more than one region per output dimension (Fig. 3). We interpret this as evidence that the patch merger processes MS data while avoiding confounding signal inputs. As a subsequent DL classifier, we implemented a light transformer network that is based on a self-attention mechanism (Fig. 1). This architecture is suitable for learning data relationships beyond regional peak patterns, allowing to capture global dependencies without an extensive number of inner network layers^10^. Given the high dimensionality of proteomic data, this might be particularly helpful, as it saves computational costs and makes the model less prone to overfitting.

To evaluate the improvement of the ProFormer pipeline over existing algorithms, we generated a proteomic data set of 213 in-depth analyzed HeLa cell samples that were treated with IFN-γ, a condition that only leads to a mild effect in the proteome^15,16^ and therefore making it difficult to identify (Fig. 2). Since we expected previously reported models to struggle with technical domain shifts, e.g. when samples were not acquired in the same batch, we evaluated predictions on a test set that was collected months after the training set, simulating a time-distinct sample drawing. In this first scenario, we found that the ProFormer outperformed traditional ML algorithms (Logistic Regression, Random Forest, and XGBoost) and was able to generalize on the classification problem, while all 16 included image-based CNN approaches showed a substantial drop in their performance in comparison to the validation set (Table 1). In an external study that was coherently acquired, we emphasized this result and introduced noise to the features of the test samples to make them appear different to the initial training set. Similar to our previous observation, the classification accuracy of CNNs decreased with increasing noise ratio, while the ProFormer’s performance remained highly robust (Fig. 4F). This constitutes an essential finding as it suggests that post hoc measurement of test samples can be classified by the ProFormer irrespective of the time and instrument calibration, and might therefore even be robust to other limiting factors, i.e., unseen dimensions. Indeed, building upon an extensive experimental setting that involved culturing and injection of almost 400 individual cell cultures, we demonstrated the ProFormer’s capability to generalize across biological domains (Scenario 2) and effect sizes (Scenario 3), providing accurate predictions for IFN-γ treatment down to 10-times lower concentration than in the training material (Fig. 2I). In comparison, image-based DL classifier did not produce robust models when confronted with this multi-source domain generalization task, suggesting that their high binary classification accuracy results from discriminating two fixed states rather than learning a contextualized biological representation. Conversely, the ProFormer’s remarkable performance positions it above other DL architectures when developing generalized models from raw proteomic data in future applications.

As a meaningful addition, the ProFormer’s tabular input offers significant structural benefits, allowing the inclusion of additional signal features. For instance, we proposed the use of ion mobility, which separates isobaric peptides and conveniently leads to higher signal-to-noise during data acquisition^9^. In this work, using the ion-mobility feature in the ProFormer resulted in around 3.7 percentage points higher AUC in the validation set for our in-house IFN-γ experiment (Fig. 2C, Scenario 1). This is despite the fact that ion mobility has the lowest resolution (R ~ 50^37^) among the used peptide ion features, while also offering further potential for optimization. In this regard, dividing the mass and charge features has significantly improved the performance of our plasma proteome classifier (Fig. 5), which increased the validation set AUC from 72.7% (with m/z feature) to 86.3% (Supplementary Fig. 6A), demonstrating that the implementation of multiple features can have a substantial impact on the tuning process.

Furthermore, we have shown that sample classification based on peptide ion profiles is not restricted to a high proteome coverage, as we applied the ProFormer to a number of recently published single-cell data sets, for which we achieved particularly high accuracies (Table 2). In this emerging field, the annotation of cells prior to downstream analysis is urgently needed, and our architecture might thus serve as a template to develop such tools, as it showed the ability to identify cells by their cell type or cycle stage with accuracies close to 100%. Since single-cell proteomic data typically does not reach the same quality as for bulk samples, we emphasized the importance of ensuring high-quality inputs for AI models by filtering low-abundance signals (Fig. 4C, D). In addition, we anticipate that heterogeneity among single cells requires special focus on creating large data sets for model training (Fig. 4E).

Beyond the analysis of single cells, we demonstrated the implementation of plasma proteome samples, and specifically applied the ProFormer to the challenging classification of patients with severe COVID-19 among several other (infectious) disease conditions in the COMBAT consortium study^25^ (Fig. 5). Here, we achieved accuracies of more than 86%, and the model could be tuned to identify the vacant disease with 100% sensitivity, while still resulting in almost 50% specificity (Fig. 5C, D). This encourages the use of the ProFormer for these biologically diverse samples. Importantly, to facilitate confidence in the predictions of the ProFormer, we made use of the SHAP value system, identifying peptide ions with high importance for the model, making its decisions explainable. In this respect, the ProFormer has an essential advantage over previous image pipelines, as the latter’s binning approach only allows the analysis of regional attentions, which are based on the aggregation of peptide ions with similar mass or retention time features that are in fact fundamentally unrelated. In contrast, predictions of the ProFormer can be interpreted at a single-peptide ion resolution, benefiting from the preservation of signal information when processing tabular data, which constitutes a strong advantage for its explainability. Consequently, using a SHAP Gradient Explainer, we found several peptide ions with close attention, whose strong discriminatory power could be verified using them as input for a simpler ML classifier (Fig. 5H). Moreover, these peptide ions showed inherently different intensities between the patients’ plasma proteomes (Fig. 5J), reflecting similar processes identified by the original COMBAT study via a conventional proteomic data analysis strategy^25^. This demonstrates that decisions by the ProFormer are based on actual proteome differences, testifying to its potential in practical settings. It further implies that the ProFormer’s capability pairs sample classification with explainability through the use of SHAP values, permitting the identification of peptide ions to enhance mechanistic understanding of underlying biological processes or to serve as biomarkers.

To evaluate the proof of concept of the ProFormer pipeline, we leveraged established software tools to reliably process and extract MS1 features from LC-MS/MS raw data in this work. Simultaneously, peptide identifications derived from the corresponding MS2 spectra allowed us to assess if the ProFormer output was biologically meaningful. For instance, COVID-19-associated MS1 features recognized by the ProFormer corresponded to peptide ions that were identified as differentially abundant using conventional statistical analysis (Fig. 5I, J), supporting the biological validity of the ProFormer results. Importantly, this was achieved by the ProFormer’s peptide- and protein-agnostic design; however, we note that this work relied on MS1 feature extraction that was informed by MS2-based peptide identification. This strategy was sufficient to establish the ProFormer concept (Figs. 1, 2), gain insights into its learnable approach (Fig. 3), establish explainability at single-peptide resolution (Fig.5I, J), and explore initial use cases in diverse biological scenarios, including cellular perturbation (Fig.2), single cells (Fig. 4), and plasma (Fig. 5). Together, these results provide an encouraging foundation for further methodological advancements. Specifically, we envision the ProFormer to operate in a fully database search-free manner, using MS1 features independent of MS2 information, or even in the absence of MS2 data. This will require substantial algorithm development to process raw MS data, with the potential to provide richer MS1 features as an input for the ProFormer. Additionally, decoupling from MS2 could motivate experimental MS acquisition strategies optimized for MS1 measurements, potentially offering the added advantage of increased data acquisition throughput.

Collectively, the ProFormer provides an innovative approach for the accurate classification of proteomic samples ranging from plasma to bulk proteomes and single cells. We expect this to be a pivotal step in advancing protein analysis toward clinical applications by enabling rapid annotation of large sample cohorts in plasma and in single-cell proteomics.

## Methods

### Cultivation of HeLa cells and Interferon-gamma treatment

Human HeLa S3 cells were cultivated in DMEM medium (Gibco), supplemented with 10% (v/v) fetal bovine serum (FBS, Gibco) and 2 mM GlutaMAX (Gibco). For the initial ML evaluation experiment (Scenario 1), we cultivated six HeLa cell cultures on a large scale using 10-cm dishes. As cells reached 70% confluence, FBS concentration was reduced to 3% (v/v), and cultures were incubated for 1 hour until three dishes received treatment with 100 ng/mL recombinant IFN-γ (Cell Signaling, diluted in phosphate-buffered saline (PBS, Sigma-Aldrich)) for 24 h. To evaluate the ProFormer with biologically distinct samples (Scenario 2), we followed the same procedure but cultivated HeLa cell samples in instances of around 30,000 cells (*N* = 64) in a 96-well format before treating half the cultures with 100 ng/mL IFN-γ. For the concentration series experiment (Scenario 3), we prepared three and a half 96-well plates with instances of 30,000 cells (*N* = 315) and applied seven different IFN-γ concentrations (0.001, 0.01, 0.1, 1.0, 10.0, 20.0, 50.0 ng/mL) to half the plate (*N* = 45), respectively.

After incubation, the culture medium was aspirated, and cells were washed with pre-warmed PBS. Adherent cells were detached by gentle scraping in 10 mL PBS, and afterwards sedimented at 1000 x g for 5 min. The supernatant was discarded and cells were washed once in 5 mL ice-cold PBS followed by centrifugation at 1000 x g for 5 min and removal of the remaining buffer from the pellet. Cells were frozen and stored at -80 °C, preserving their integrity until their following preparation.

### Preparation of proteomic samples

Obtained pellets of treated and control cells were thawed and lysed in 50 mM TEAB, pH 8.5 (Sigma-Aldrich) buffer + 8 M urea (Bio-Rad) and 1x cOmplete™ protease inhibitor (Sigma-Aldrich). The lysate was sonicated for 15 cycles of 30 s ON/OFF at 4 °C using a Bioruptor (Diagenode), frozen at -80 °C, and afterwards separated from cell debris by centrifugation at 18,000 x g. Samples were transferred to new vials before protein reduction by adding 10 mM final concentration of dithiothreitol (DTT, Biomol) and incubation at 37 °C and 600 rpm for 45 min. Cysteine residues were alkylated by 55 mM final chloroacetamide (CAA, Sigma-Aldrich) at RT and 600 rpm in the dark for 30 min. Samples were diluted in a 1:6 ratio using digestion buffer (50 mM TEAB + 2 mM CaCl_2_ (Sigma-Aldrich), pH 8.5) to an urea concentration of <1.6 M and proteins were digested overnight at 37 °C and 800 rpm using sequencing-grade modified trypsin (Promega) at a protein-enzyme ratio of 25:1. After digestion, peptides were cleaned-up on Sep-Pak cartridges (Waters) or self-assembled StageTips containing C_18_ material. The columns were prepared by applying pure ACN (Biosolve Chimie), solvent B (50% (v/v) ACN + 0.1% (v/v) FA (Biosolve Chimie) in ddH_2_O) and solvent A (0.1% (v/v) FA in ddH_2_O), before loading the acidified peptide solution. Samples were washed twice with 1 mL solvent A, and eluted twice in 200 µL solvent B. Purified peptides were frozen at −80 °C, lyophilized in a freeze-dryer and afterwards reconstituted in solvent A. For samples from the six cell culture dishes, peptide concentration was determined by BCA assay (Pierce, Thermo Scientific) before sample injection.

### Data acquisition (LC-MS/MS)

In this work, we used a diaPASEF LC-MS methodology that was established in our lab for the conventional measurement of proteomic samples, including fragmentation (sequencing) of peptide ions to infer their protein identity, while we only used MS1-derived signal features of identified peptides for all subsequent AI classification models.

In our initial IFN-γ experiment for ML evaluation, we injected 100-ng peptide samples of six HeLa-cell cultures (control and treatment condition; *N* = 3 biological replicates each) in technical replicates, acquiring a total 142 data files for model training. Aliquots of the same samples were kept frozen and were re-measured in around six months, creating a second set of 71 files for model testing. Injection was performed in 2-µL volumes using an EASY-nLC 1200 system (Thermo Scientific) that was connected to a timsTOF Pro mass spectrometer (Bruker Daltonics) via a nano-flow electrospray ionization (nano-ESI) source (Captive Spray, Bruker Daltonics). Peptides were separated on an analytical column (IonOpticks Aurora Series Gen3, 25 cm × 75 µm i.d. + CSI, 1.6 µm C18) using an active gradient of 34 min, ranging from 1.6% (v/v) to 28% (v/v) ACN in ddH_2_O (both Biosolve Chimie) at a flow rate of 300 nL/min. During separation, the analytical column was kept at a temperature of 50 °C in a column oven (Sonation). In the biologically distinct IFN-γ experiments of multi-replicate tests and the concentration series, we adjusted sample injections from 30,000 cells to 100-ng peptide amount by inspecting the total ion chromatogram (TIC). Injections were performed in 50-nL volumes using a Vanquish Neo System (Thermo Scientific), and peptides were separated on a newer version of the same analytical column (IonOpticks Aurora Series Gen4) with an adjusted active gradient to resemble peptide separation from the previous generation, ramping from 5.6% (v/v) to 30.4% (v/v) ACN in ddH_2_O in 34 min at a flow rate of 300 nL/min. When peptides arrived at the ion source, they were ionized at a capillary voltage of 1,500 V, a dry gas flow rate of 3.0 L/min and at a temperature of 180 °C. In the MS, ions were accumulated to an IM constant 1/K_0_ of 1.7 V*s/cm^2^ and sequentially ramped from 1.25 to 0.65 V*s/cm^2^ over 100 ms in a locked duty cycle. Subsequent MS1 scans were acquired for peptide signals between 200 and 1700 m/z, while only precursors with a mass ratio of 475 to 925 m/z were considered for DIA window isolation. Eight DIA scans with window widths of 25 m/z and a height of 0.15 V*s/cm^2^ resulted in a cycle time of 0.95 s. Precursor fragmentation was induced by IM-dependent collisional energies from 45 eV at 1/K_0_ of 1.3 V*s/cm^2^ to 27 eV at 0.75 V*s/cm^2^.

### Raw data processing

Following the measurement, diaPASEF raw data were processed in DIA-NN (v1.8.0)^38^ using its library-free analysis involving the in silico digestion of a UniProtKB sequence table (FASTA) from *Homo sapiens* (downloaded in September 2021, containing 20,386 reviewed proteins). We allowed a maximum of two missed cleavage sites per peptide and set the peptide length range to 7–50 and charge states to 2–4. We activated “MBR” to increase data completeness of the respective dataset and let protein groups be formed according to protein names from FASTA. We selected “any LC (high precision)” for precursor quantification, but later relied on unnormalized MS1 signal quantities from the output report table. Notably, we processed raw data of training and test in two independent processes using equivalent settings.

For the analysis of external single-cell data sets acquired in DIA mode, we equivalently re-processed raw data in DIA-NN (v1.8.0) but reduced the number of possible missed cleavage sites to one. In the case of mESC data, we used a FASTA file derived from *Mus musculus* (downloaded in March 2023 with 17,140 review proteins) and further allowed charge states up to five, considering the Orbitrap mass analyzer. Re-analysis of single-cell DDA data (SC study #1, Table 2) was performed in MaxQuant (v2.4.2.0)^39^ maintaining its default configuration and with “MBR” activated. As protein sequence table, we used the identical FASTA file derived from *Homo sapiens*. Resulting peptide tables were filtered at 1% FDR.

### Preparation of datasets for deep learning pipelines

After raw data processing, we grouped data from individual samples in the output report table and extracted the respective peptide ion information on sequence, charge state, indexed retention time, indexed ion mobility and MS1 intensity (Ms1.Translated). Here, the use of indexed dimensions fixed them in a uniform range, allowing similar model training when applying different gradient lengths. To calculate monoisotopic peptide ion masses, we used the “molecular_weight” function in the Bio.SeqUtils.ProtParam (v1.8.1)^40^ Python module and obtained mass-to-charge ratios from the respective charge states. Resulting data tables contained MS1 signal profiles of peptides identified via their MS2 information, which have been used throughout this work to tune and test all AI pipelines.

Due to the high number of missing values in some single-cell studies, we filtered low-identification runs at a threshold of 200 and 800 peptide ions per cell in the works of Hu M et al.^21^ (SC study #1) and Petrosius V et al.^24^ (SC study #6, Table 2), respectively, to increase data consistency.

### ProFormer pipeline for tabular data

Before model training, obtained peptide ion profiles were processed to generate a uniform input: feature dimensions of retention time, mass-to-charge, intensity, and ion mobility were z-score standardized, respectively. These steps ensure that all features have the same scale and increase consistency between samples, allowing efficient and stable training. Notably, we did not include column embedding, thus fixing the order of feature columns for training and testing. Since individual samples might contain more than a hundred thousand peptides but show incomplete overlap, i.e., missing values, we used a patch merger^14^ to reduce the dimensionality of the resulting matrix to smaller, equally sized token sets. In this step, sample matrices are divided into non-overlapping patches, each aggregated into one token based on their global context using a self-learning merge mechanism. An additional CLS token is concatenated to the resulting set of tokens, representing its aggregated information, before passing the matrix to the transformer. The ProFormer architecture consists of *N* transformer layers, based on the architecture proposed by the work of Vaswani et al.^10^, and a classifier head. Each transformer block includes a multi-head attention, followed by a multi-layer perceptron and a normalization layer with residual connections and drop-outs. The self-attention mechanism of a transformer allows identifying complex structures by an input mapping function. In doing so, each attention head performs three linear learning projections to obtain so-called query, key, and value matrices. To determine the output of the self-attention mechanism, a weighted sum of the value matrix is calculated, where the weights are a dot product of the query and key matrices, allowing the model to observe global dependencies. For our desired classification task, we could solely rely on an encoder block as a transformer layer. Passing through the encoder blocks, the CLS token is gradually adjusted and used as input for the subsequent classification head to determine the predicted label of the input matrix. During the training process, we used adamax^41^ optimization, applied through a cosine warm-up scheduler^42^. This pipeline has been implemented in Python code, which can be found in a GitHub repository (see Code Availability section), including all utilized packages. Specifically, the pipeline was implemented using Python (v3.11.5) with CUDA (v11.7), PyTorch (v2.0.1), pytorch-lightning (v2.0.8), torchvision (v0.15.2), and einops (v0.6.1).

Addressing the issue of imbalanced datasets, specifically in the context of the tested plasma proteome samples, we further implemented a weighted random sampler, ensuring that sample classes are considered equally by oversampling the underrepresented one and vice versa, and a weighted loss function to assign greater detriment to a misclassification event of the underrepresented class. During model tuning, detriments were optimized as hyperparameters.

### Image pipeline

For CNN models, identified peptide ion profiles were converted to an image, representing retention time on one axis and mass-to-charge on the other axis. To create a uniform data input, images were divided along a grid structure, and the resulting boxes were binned by their respective summed intensities, followed by normalizing the resulting values per pixel to a range of 0 to 1. Based on previous observations from J. Cadow et al.^4^, we mainly relied on a ResNet_v2_50^43^, a CNN with a depth of 50 hidden layers that includes batch normalization before each weight layer and Rectified Linear Unit (ReLU) activation functions. ResNet makes use of the residual learning concept, which includes skip connections to jumps over certain layers, allowing the training of very deep neural networks and thus the detection of complex structures. In addition, J. Cadow et al. used a pre-trained network based on the LSVRC-2012-CLS dataset^44^ for image classification. In pre-trained image-based models, early layers have already been accustomed to structures, such as edges, textures, and basic shapes. Since generated peptide ion images do not contain such geometric structures, the classification was also performed on a ResNet_v2_50 without pre-trained weights. Other image-based architectures included CNNs, namely ResNet_v2_18^43^, ResNet_v2_101^43^, EfficientNet_b0^45^, EfficientNet_b1^45^, DenseNet121^46^, Inception_ResNet_v2^47^, and the Vision Transformer ViT_Base_Patch16_224^12^. All image pipelines were loaded via timm (v0.9.7).

### Traditional machine-learning pipelines

Peptide ion features were preprocessed equivalent to the description for the ProFormer, and data matrices were condensed using one of three dimensionality reduction approaches: Wavelet transform, K-means clustering or the patch merger trained prior in the ProFormer pipeline. Consequently, we provided subsequent ML algorithms with compact representations of the initial peptide ion profiles with a fixed number of features and dimensionality. Reduced features were flattened into a tabular form and used as input for three models: Random Forest, XGBoost, and Logistic Regression. Hyperparameters were tuned using a Bayesian search. Final models were retrained on the complete training set with optimized hyperparameters and finally evaluated on an unseen test set. All machine-learning models were implemented in Python using Scikit-learn (v1.4.2) and XGBoost (v2.1.4).

### Model tuning

All implemented models were tuned by Optuna (v3.5.0)^48^, based on a tree-structured estimator sampler^49^ that fits a Gaussian mixture model for each hyperparameter setting. While training and test data were recorded semi-independently for our in-house IFN-γ experiment, external studies only consisted of one coherent data set. We therefore randomly sampled data of these studies into two groups, respectively, tuning models on one group while performing model evaluation only on previously unseen data. Using a stratified split, training data was divided into five groups, maintaining equal distribution of both labels (experimental conditions). Since the number of samples in proteomic data sets is usually limited, we tuned models by aiming for the highest average accuracy (AUC) among all five validation sets in a five-fold cross-validation procedure. Subsequently, models were trained with optimal parameter settings using the entire training set, and we assessed their performance on the test data.

### XAI approach by SHAP values

Model decisions were assessed by SHAP (SHapley Additive exPlanations) (v0.45.0)^26^, established on the Aumann-Shapley game theory^50^, which provides a framework for interpreting the output of machine learning models. This method assigns a metric to features of individual data points to estimate their contribution to the model’s prediction. To this end, we used the Gradient Explainer method^51,52^ that approximates SHAP values according to the mathematical gradients of the model output and by considering exemplary input features of reference data points from the training set. As a result, the gradient vector indicates the change rate of the model output for each feature compared to the baseline input. Their sum can be seen as the cumulative impact, while positive values represent a contribution towards label 1 and negative values represent a contribution towards label 0.

### Random forest model for XAI evaluation

In the analysis of the COMBAT study^25^, we validated whether the SHAP value sum per peptide ion can serve as an importance metric for the ProFormer model. We identified peptide ions with the highest positive and negative average SHAP value sums (potential high impact) from test sample classes 1 and 0, and we applied their respective features from the training set to tune a Random Forest classifier using Scikit-learn (v1.4.2). To this end, data was flattened and padded to ensure samples had the same input dimensions, and we evaluated performance using the test dataset, comparing it to the results from peptide ions with average SHAP value sums close to zero (potential low impact).

### Statistical analyses and data illustration

Following raw data analysis of proteomic samples and tuning DL pipelines, model predictions were evaluated using Python code (v3.9.12) in the Jupyter Notebook environment (v6.4.8)^53^. We used Pandas (v1.4.2) and NumPy (v1.21.6) modules for data handling and calculations, including correlation analyses, and Matplotlib (v3.5.1) and Seaborn (v0.13.1) for data plotting. To fit the model predictions in the concentration series experiment, we relied on the “curve_fit” function from SciPy (v1.15.2) using a logistic function in the Hill-type form:

1 $${L}_{\min }+\,\frac{{L}_{\max }-{L}_{\min }}{1+\,{\frac{c}{{c}_{0}}}^{k}}$$

With *L*_min_: the lower limit, *L*_max_: the upper limit of predictions, *c*: the IFN-γ concentration, *c*_*0*_: the infliction point (LoD) and *k*: the curve’s slope. Dimensional reduction by PCA and tSNE^54^ were performed using Scikit-learn (v1.4.2).

### Reporting summary

Further information on research design is available in the Nature Portfolio Reporting Summary linked to this article.

## Supplementary information

Supplementary Information

Supplementary Data 1

Supplementary Data 2

Supplementary Data 3

Supplementary Data 4

Supplementary Data 5

Reporting Summary

Transparent Peer Review file

## Source data

Source data

## Data Availability

The acquired raw LC-MS/MS data in this study, processed report files and extracted peptide ion features have been deposited in the ProteomeXchange Consortium via the PRIDE partner repository under the accession codes PXD059121 (IFN-γ training and test data of initial ML evaluation), PXD077213 (IFN-γ multi-replicate test set), PXD077023 (IFN-γ concentration series test set – 50 ng/mL), PXD077039 (20 ng/mL), PXD077047 (10, 1 & 0.1 ng/mL) and PXD077061 (0.01 & 0.001 ng/mL). The data generated in this study are provided in the Supplementary Information/Source Data file. Source data are provided with this paper.

## Code availability

The code used to develop the model/perform the analyses and generate results in this study is publicly available and has been deposited in GitHub at https://github.com/DBO-DKFZ/ProFormer, under MIT license. The specific version of the code associated with this publication is archived in Zenodo and is accessible via https://doi.org/10.5281/zenodo.21511818^55^.
