# Supplementary material for: ProFormer: generalizable classification of single-cell and plasma proteomes using deep learning
Source: Nat Commun. 2026 Oct 5;17:10493. doi: 10.1038/s41467-026-78143-2 (PMC13639084; doi:10.1038/s41467-026-78143-2)
Supplement: Supplementary file 1 [file 41467_2026_78143_MOESM1_ESM.pdf]

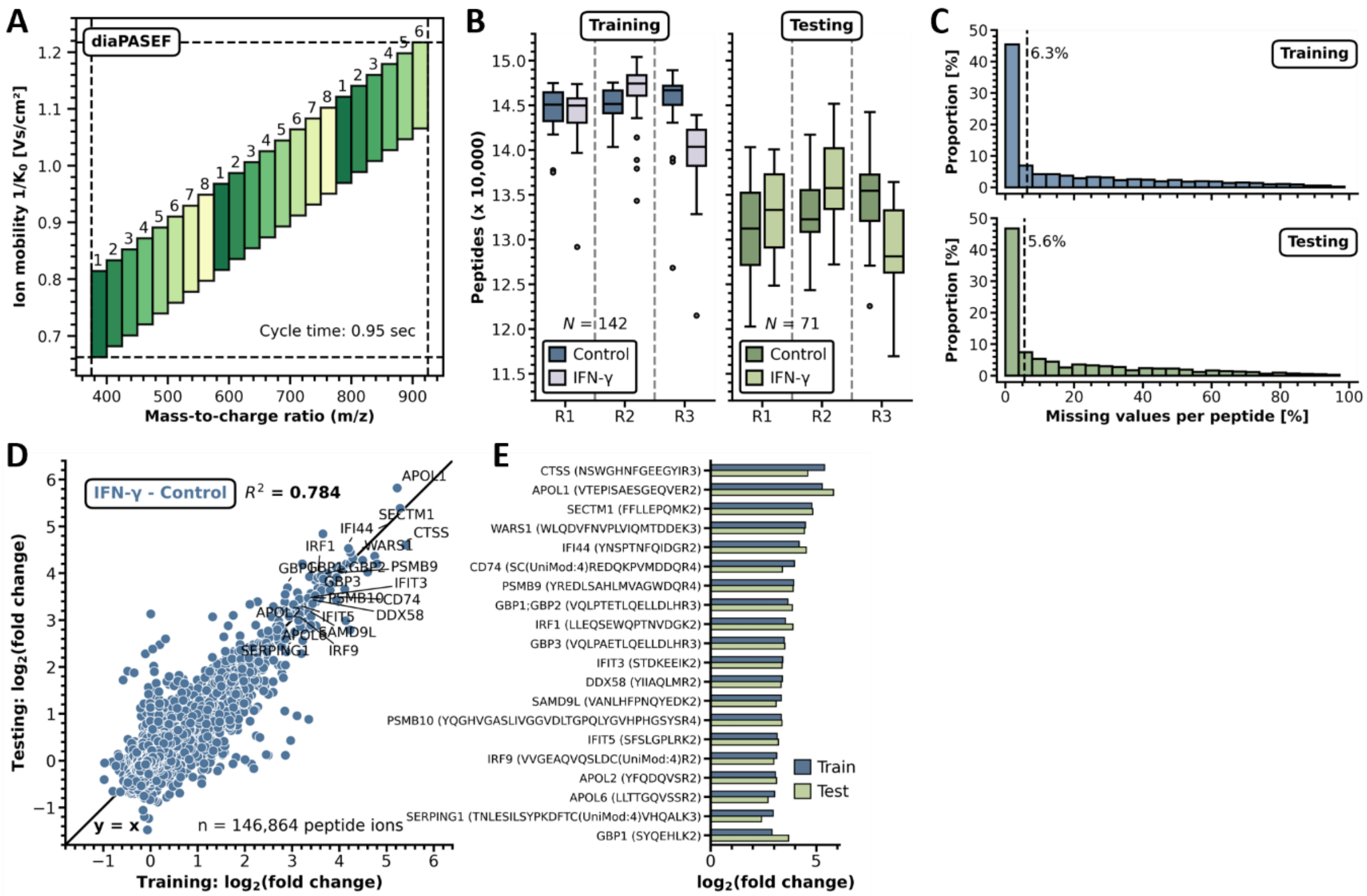

Supplementary Figure 1: (A) diaPASEF method scheme for MS2 data acquisition. Window numbers were consecutively isolated in one ion mobility ramp. (B) Boxplot distribution of identified peptide ions per sample and condition in the training set (left – blue: control, lavender: IFN-γ-treated) (*N* = 142) and test set (right – green: control, light green: IFN-γ-treated) (*N* = 71). Boxes show first and third quartile with the median as central line. Whiskers show 1.5-x the interquartile range, while grey dots indicating outlying samples. (C) Histogram of relative missing values per peptide ion across training (top, blue) (*N* = 142) and test samples (bottom, green) (*N* = 71). Dashed lines indicate median missing values per peptide ion per set. (D) Correlation of IFN-γ effect on peptide ions (n = 146,864) between training (x-axis, *N* = 142) and test set (y-axis, *N* = 71). Within each set, fold changes were calculated by dividing the mean peptide ion abundance in the IFN-γ condition by the control condition. A solid black line shows a perfect correlation with x = y. Coefficient of determination R^2 indicated at the top. Peptide ions with the highest fold changes in the training set were annotated with the name of their parental protein. (E) Highest fold change peptide ion per most regulated proteins (n = 20), compared between training (blue) and test set (green) based on data in D. Source data are provided as a Source Data file.

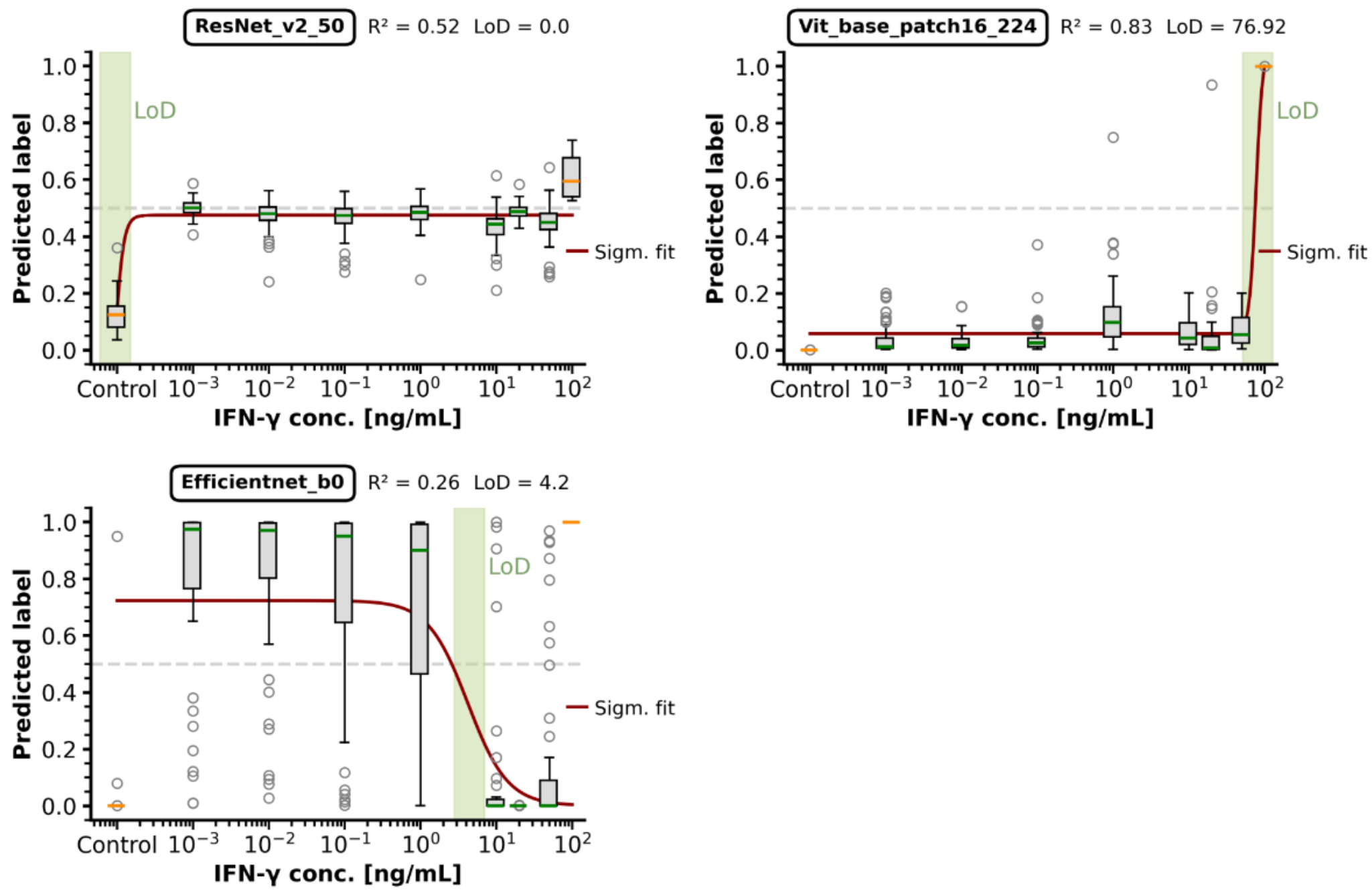

Supplementary Figure 2, complementing Figure 2: Box plot distributions of model predictions in scenario S3 using different DL pipelines (Top left: ResNet_v2_50; Top right: ViT-Base; Bottom left: EfficientNet-B0) across a range of IFN-γ concentrations. Boxes show the first to third quartile with the median as colored central line (Orange: control and 100 ng/mL IFN-γ (*N* = 16 each); Green: seven intermediary concentrations not contained in the training set (*N* = 45 each)). Whiskers indicate 1.5-times the inter-quartile range, with outliers shown as circles. The red line shows a sigmoidal fit for the predictions, with the inflection point marking the model's limit of detection (LoD, green). Coefficient of determination R^2 indicated on the top. Source data are provided as a Source Data file.

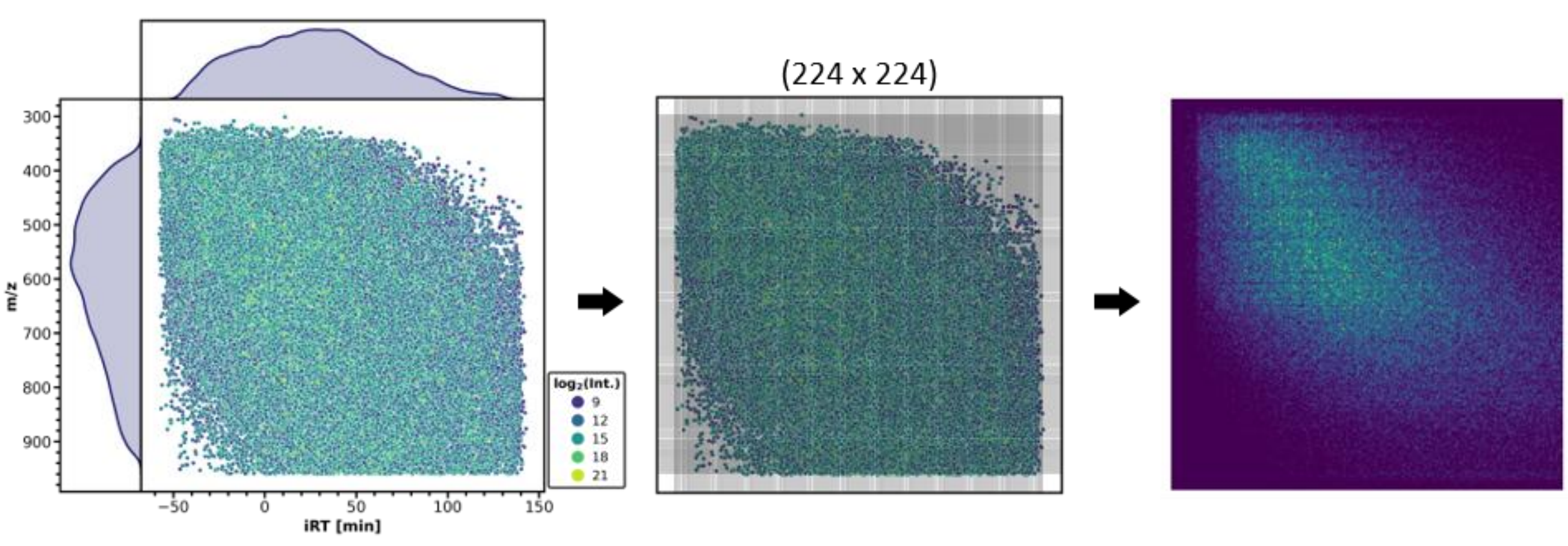

Supplementary Figure 3: Image-conversion of LC-MS data using a 224 x 224 grid. Left: Scattered heatmap of identified peptide ions in the IFN-γ training set (around 150,000) across the iRT-to-m/z plane, with colors indicating the MS1 peak area. Kernel density of peptide ions on the top and at the left side show the distribution across the respective feature. Middle: 224 x 224 grid format applied to the peptide ion map to bin signals with proximate iRT or m/z feature. Right: Image-like heatmap representation generated by transferring the binned MS1 intensities into the rgb space, used as input for image pipelines (vision transformer and CNN architectures).

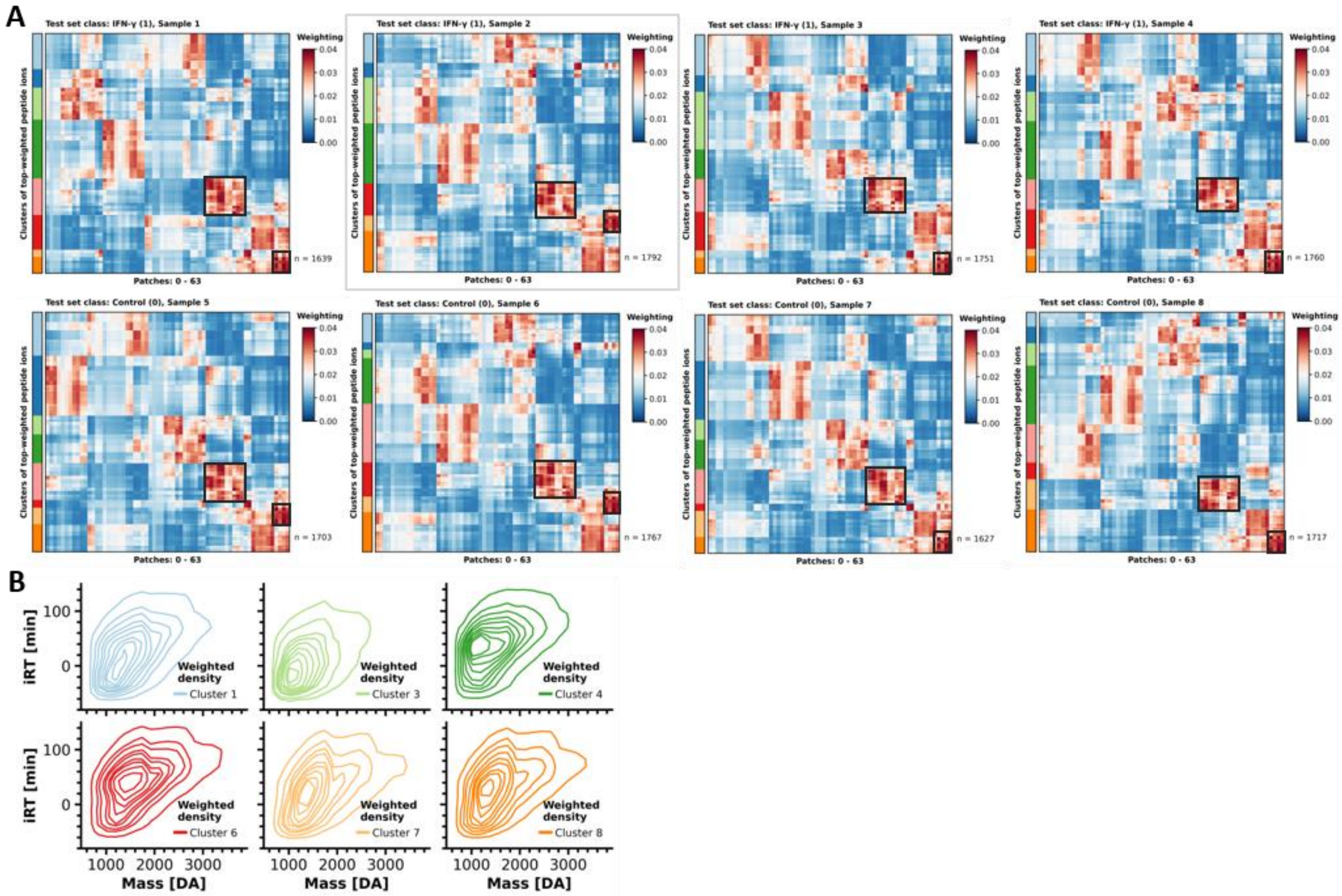

Supplementary Figure 4, complementing Figure 3: (A) Heatmap representation of the attention weight matrix $\mathbf{W_A}$ in the patch merger (see 3A) clustered by Euclidean distance for a random set of eight test set samples of the in-house IFN-γ experiment (4x control & 4x IFN-γ-treated). Only top-weighted peptide ions per output patch across all eight samples were included (differences in heatmap sizes arise from missing values), resulting in different matrix dimensions as indicated by the number n per heatmap. Color bar on the left shows eight peptide ion clusters with high attention weights found across all examined test set samples (two exemplary clusters highlighted by a black frame). Sample 2 (indicated by a grey frame) is highlighted in main Figure 3B. (B) Mass-to-iRT density distribution of patch-merger weights for peptide ions of patch clusters in samples 2 (same color-coding). The Top-10% highest density regions per cluster are highlighted as overlay in main Figure 3D. Source data are provided as a Source Data file.

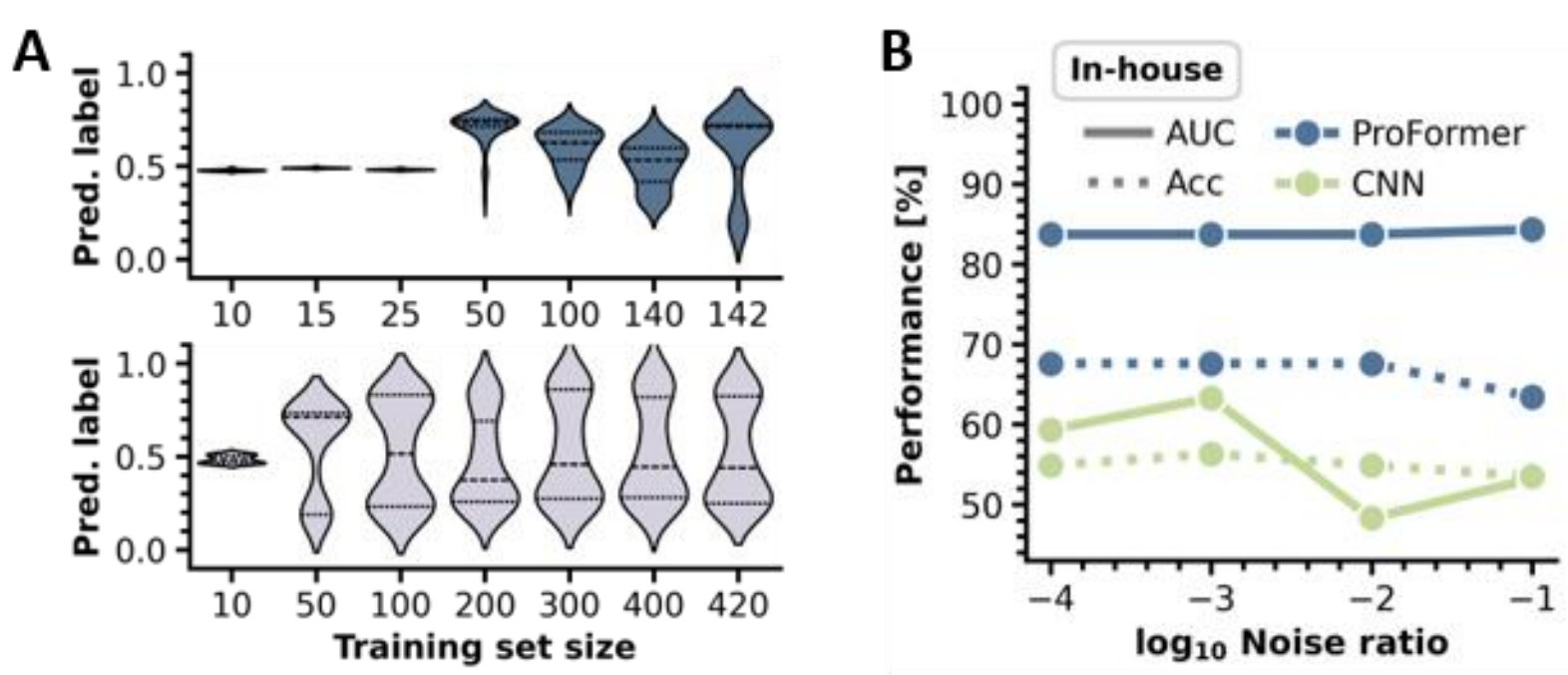

Supplementary Figure 5, complementing Figure 4: (A) Violin plot showing the test set predictions of the ProFormer (top: in-house IFN-γ experiment in S1 (blue), bottom: SC study #6 (see Table 2) (violet)) after the model was trained with different training set sizes. Inner dashed lines indicate median, first and third quartile of the distribution. (B) Classification performance (AUC: solid, Accuracy: dotted line) of the ProFormer (blue) and a CNN image-pipeline (ResNet_v2_50, green) on the in-house IFN-γ data with varying ratios of Gaussian noise applied to each of the test set features. Source data are provided as a Source Data file.

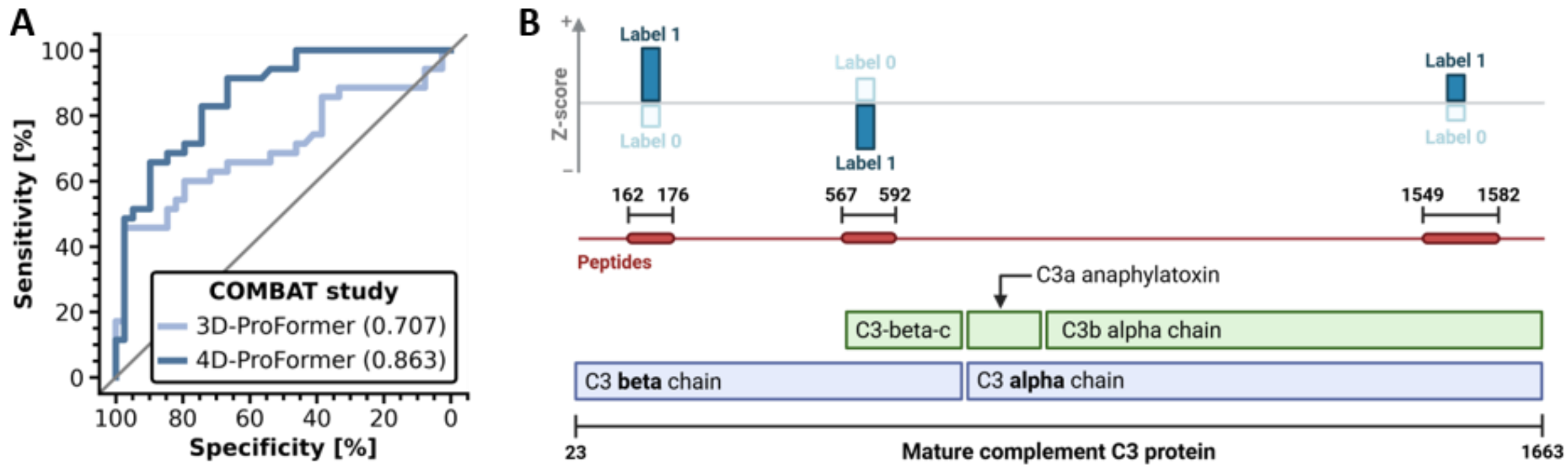

Supplementary Figure 6, complementing Figure 5: (A) ROC analysis of a 3D-Proformer (Light blue: implementing m/z, RT and intensity features) and a 4D-Proformer (Dark blue: implementing mass, charge, RT and intensity features) trained on the COMBAT data set. AUCs indicated in parenthesis. A solid line indicates a random classification. (B) Composition of the complement protein C3 (from bottom to top). Bottom (blue and green): Broad elements in the mature protein exerting individual functions after proteolytic cleavage upon complement system activation. Middle (red): Sequence localization of C3 peptides with high impact to the predications of the 4D-ProFormer model (shown in 5J) according to SHAP values calculations. Top: Z-scored peptide intensities in the underlying proteomic data in samples of class 1 and class 0 (equivalent to 5J), indicating the regulation of respective C3 protein elements in severe Covid-19 (label 1). Numbers show amino acid positions in the protein sequence. Source data are provided as a Source Data file.
