# Supplementary material for: ProFormer: generalizable classification of single-cell and plasma proteomes using deep learning
Source: Nat Commun. 2026 Oct 5;17:10493. doi: 10.1038/s41467-026-78143-2 (PMC13639084; doi:10.1038/s41467-026-78143-2)
Supplement: Supplementary file 2 [file 41467_2026_78143_MOESM7_ESM.pdf]

Corresponding author(s): Titus Brinker
Jeroen Krijgsveld

Last updated by author(s): Sep 3, 2026

Reporting Summary

Nature Portfolio wishes to improve the reproducibility of the work that we publish. This form provides structure for consistency and transparency in reporting. For further information on Nature Portfolio policies, see our Editorial Policies and the Editorial Policy Checklist.

Statistics

For all statistical analyses, confirm that the following items are present in the figure legend, table legend, main text, or Methods section.

n/a	Confirmed	
☐	☒	The exact sample size (*n*) for each experimental group/condition, given as a discrete number and unit of measurement
☐	☒	A statement on whether measurements were taken from distinct samples or whether the same sample was measured repeatedly
☐	☒	The statistical test(s) used AND whether they are one- or two-sided<br>*Only common tests should be described solely by name; describe more complex techniques in the Methods section.*
☒	☐	A description of all covariates tested
☐	☒	A description of any assumptions or corrections, such as tests of normality and adjustment for multiple comparisons
☐	☒	A full description of the statistical parameters including central tendency (e.g. means) or other basic estimates (e.g. regression coefficient) AND variation (e.g. standard deviation) or associated estimates of uncertainty (e.g. confidence intervals)
☐	☒	For null hypothesis testing, the test statistic (e.g. *F*, *t*, *r*) with confidence intervals, effect sizes, degrees of freedom and *P* value noted<br>*Give P values as exact values whenever suitable.*
☒	☐	For Bayesian analysis, information on the choice of priors and Markov chain Monte Carlo settings
☒	☐	For hierarchical and complex designs, identification of the appropriate level for tests and full reporting of outcomes
☐	☒	Estimates of effect sizes (e.g. Cohen's *d*, Pearson's *r*), indicating how they were calculated

Our web collection on statistics for biologists contains articles on many of the points above.

Software and code

Policy information about availability of computer code

Data collection	A timsTOF Pro mass spectrometer (Bruker Daltonics) in combination with an EASY-nLC 1200 or a Vanquish Neo system (both Thermo Scientific) was used for data acquisition. 100-ug peptide samples were analyzed using a 34 min active gradient followed by diaPASEF mode acquisition.
Data analysis	diaPASEF raw data was processed in DIA-NN (v1.8.0) using library-free analysis involving the in-silico digestion of a UniProtKB sequence table (FASTA) from Homo sapiens (downloaded in September 2021, containing 20,386 reviewed proteins). We allowed a maximum of two missed cleavage sites per peptide and set the peptide length range to 7 – 50 and charge states to 2 – 4. We activated “MBR” to increase data completeness of the respective data set and let protein groups be formed according to protein names from FASTA. We selected “any LC (high precision)” for precursor quantification, but later relied on unnormalized MS1 signal quantities from the output report table. For the analysis of external single-cell data sets acquired in DIA mode, we equivalently re-processed raw data in DIA-NN (v1.8.0) but reduced the number of possible missed cleavage sites to one. In case of mESC data, we used a FASTA file derived from Mus musculus (downloaded in March 2023 with 17,140 review proteins) and further allowed charge states up to five, considering the Orbitrap mass analyzer. Re-analysis of single-cell DDA data (SC study #1, Table 2) was performed in MaxQuant (v2.4.2.0) maintaining its default configuration and with “MBR” activated. As protein sequence table, we used the identical FASTA file derived from Homo sapiens. Resulting peptide tables were filtered at 1% FDR.<br><br>After raw data processing, we grouped data from individual samples in the output report table and extracted peptide ion information on sequence, charge state, indexed retention time, indexed ion mobility and MS1 intensity (Ms1.Translated). To calculate monoisotopic peptide ion masses, we used the “molecular_weight” function in the Bio.SeqUtils.ProtParam (v1.8.1) Python module and obtained mass-to-charge ratios from the respective charge states. Resulting data tables contained MS1 signal profiles of peptides identified via their MS2 information, which have been used throughout this work to tune and test all AI pipelines.<br>We filtered low-identification single-cell runs at a threshold of 200 and 800 peptide ions per cell in the works of Hu M et al. (SC study #1) and

Petrosius V et al. (SC study #6, Table 2), respectively, to increase data consistency.

Before model training, obtained peptide ion profiles were processed to generate a uniform input: feature dimensions of retention time, mass-to-charge, intensity and ion mobility were z-score standardized, respectively. Notably, we did not include column embedding, thus fixed the order of feature columns for training and testing. We used a patch merger to reduce this dimensionality of the resulting matrix to smaller, equally sized token sets. In this step, sample matrices are divided into non-overlapping patches, each aggregated into one token based on their global context using a self-learning merge mechanism. An additional CLS token is concatenated to the resulting set of tokens, representing its aggregated information, before passing the matrix to the transformer. The ProFormer architecture consists of N transformer layers, based on the architecture proposed by the work of Vaswani et al., and a classifier head. Each transformer block includes a multi-head attention, followed by a multi-layer perceptron and a normalization layer with residual connections and drop-outs. For our desired classification task, we could solely rely on an encoder block as transformer layer. Passing through the encoder blocks, the CLS token is gradually adjusted and used as input for the subsequent classification head to determine the predicted label of the input matrix. During the training process, we used adamax optimization, applied through a cosine warm-up scheduler. This pipeline has been implemented using Python (v3.11.5) with CUDA (v11.7), PyTorch (v2.0.1), pytorch-lightning (v2.0.8), torchvision (v0.15.2), and einops (v0.6.1). Addressing the issue of imbalanced data sets, specifically in the context of the tested plasma proteome samples, we further implemented a weighted random sampler, ensuring that sample classes are considered equally by oversampling the underrepresented one and vice versa, and a weighted loss-function to assign greater detriment to a misclassification event of the underrepresented class. During model tuning, detriments were optimized as hyperparameters.

For CNN models, identified peptide ion profiles were converted to an image, representing retention time by one axis and mass-to-charge by the other axis. To create a uniform data input, images were divided along a grid structure and the resulting boxes were binned by their respective summed intensities, followed by normalizing the resulting values per pixel to a range of 0 to 1. Based on previous observations from J. Cadow et al., we mainly relied on a ResNet_v2_50, a CNN with a depth of 50 hidden layers that includes batch normalization before each weight layer and Rectified Linear Unit (ReLU) activation functions. In addition, J. Cadow et al. used a pre-trained network based on the LSVRC-2012-CLS data set for image classification. However, since generated peptide ion images do not contain such geometric structures, the classification was also performed on a ResNet_v2_50 without pre-trained weights. Other image-based architectures included CNNs, namely ResNet_v2_18, ResNet_v2_101,, EfficientNet_b0, EfficientNet_b1, DenseNet121, Inception_ResNet_v2, and the Vision Transformer ViT_Base_Patch16_224. All image pipelines were loaded via timm (v0.9.7).

Peptide ion features were preprocessed equivalent to the description for the ProFormer, and data matrices were condensed using one of three dimensionality reduction approaches: Wavelet transform, K-means clustering or the patch merger trained prior in the ProFormer pipeline. Consequently, we provided subsequent ML algorithms with compact representations of the initial peptide ion profiles with a fixed number of features and dimensionality. Reduced features were flattened into a tabular form and used as input for three models: Random forest, XGBoost and Logistic regression. Hyperparameters were tuned using a Bayesian search. Final models were retrained on the complete training set with optimized hyper¬parameters and finally evaluated on an unseen test set. All machine-learning models were implemented in Python using Scikit-learn (v1.4.2) and XGBoost (v2.1.4).

All implemented models were tuned by Optuna (v3.5.0), based on a tree-structured estimator sampler that fits a Gaussian mixture model for each hyperparameter setting. While training and test data were recorded semi-independently for our in-house IFN-γ experiment, external studies only consisted of one coherent data set. We therefore randomly sampled data of these studies into two groups, respectively, tuning models on one group, while performing model evaluation only on previously unseen data. Using a stratified split, training data was divided into five groups, maintaining equal distribution of both labels (experimental conditions). Since proteomic data set size is usually limited, we tuned models aiming for the highest average accuracy (AUC) among all five validation sets in a five-fold cross validation procedure. Subsequently, models were trained with optimal parameter settings using the entire training set, and we assessed their performance by the test data.

Model decisions were assessed by SHAP (SHapley Additive exPlanations) (v0.45.0), established on the Aumann-Shapley game theory, which provides a framework for interpreting the output of machine learning models. In the analysis of the COMBAT study, we used the Gradient Explainer method, and validated whether the SHAP value sum per peptide ion can serve as an importance metric for the ProFormer model. We identified peptide ions with the highest positive and negative average SHAP value sums (potential high impact) from test sample classes 1 and 0, and we applied their respective features from the training set to tune a Random forest classifier using Scikit-learn (v1.4.2). To this end, data was flattened and padded to ensure samples had the same input dimensions, and we evaluated performances using the test data set, comparing it to the results from peptide ions with average SHAP value sums close to zero (potential low impact).

Following raw data analysis of proteomic samples and tuning DL pipelines, model predictions were evaluated using Python code (v3.9.12) in the Jupyter Notebook environment (v6.4.8). We used Pandas (v1.4.2) and NumPy (v1.21.6) modules for data handling and calculations, including correlation analyses, and Matplotlib (v3.5.1) and Seaborn (v0.13.1) for data plotting. To fit the model predictions in the concentration series experiment, we relied on the “curve_fit” function from SciPy (v1.15.2) using a logistic function in the Hill-type form.

The code used to develop the model/perform the analyses and generate results in this study is publicly available and has been deposited in GitHub at https://github.com/DBO-DKFZ/ProFormer, under MIT license. The specific version of the code associated with this publication is archived in Zenodo and is accessible via https://doi.org/10.5281/zenodo.21494273.

For manuscripts utilizing custom algorithms or software that are central to the research but not yet described in published literature, software must be made available to editors and reviewers. We strongly encourage code deposition in a community repository (e.g. GitHub). See the Nature Portfolio guidelines for submitting code & software for further information.

Data

Policy information about availability of data

All manuscripts must include a data availability statement. This statement should provide the following information, where applicable:

- Accession codes, unique identifiers, or web links for publicly available datasets
- A description of any restrictions on data availability
- For clinical datasets or third party data, please ensure that the statement adheres to our policy

The acquired raw LC-MS/MS data in this study, processes report files and extracted peptide features have been deposited in the ProteomeXchange Consortium via the PRIDE partner repository under the accession codes PXD059121 (in-house bulk IFN-y, scenario 1), PXD077213 (scenario 2), PXD077061 (scenario 3 (S3): 0.001 & 0.01 ng/mL IFN-y), PXD077047 (S3: 0.1, 1 & 10 ng/mL IFN-y), PXD077039 (S3: 20 ng/mL IFN-y) and PXD077023 (S3: 50 ng/mL IFN-y).

External raw data was downloaded from the ProteomeXchange Consortium via the MassIVE, iProX or PRIDE partner repository using the following accession codes: MSV000089625 (SC data set #1 - Hu M et al. 2023), PXD041966 (SC data set #2 and #3 - Wang Y et al. 2024), PXD024043 (Brunner A et al. 2022 - SC data set #4 and #5), MSV000092429 (Petrosius V et al. 2023 - SC data set #6), PXD053464 (Krull KK et al. 2024 - SC data set #7) and PXD023175 (COMBAT consortium study - plasma data set).

Research involving human participants, their data, or biological material

Policy information about studies with human participants or human data. See also policy information about sex, gender (identity/presentation), and sexual orientation and race, ethnicity and racism.

Reporting on sex and gender	n.a.
Reporting on race, ethnicity, or other socially relevant groupings	n.a.
Population characteristics	n.a.
Recruitment	n.a.
Ethics oversight	n.a.

Note that full information on the approval of the study protocol must also be provided in the manuscript.

Field-specific reporting

Please select the one below that is the best fit for your research. If you are not sure, read the appropriate sections before making your selection.

[x] Life sciences [ ] Behavioural & social sciences [ ] Ecological, evolutionary & environmental sciences

For a reference copy of the document with all sections, see nature.com/documents/nr-reporting-summary-flat.pdf

Life sciences study design

All studies must disclose on these points even when the disclosure is negative.

Sample size	This study comprised a total of 595 injections. Our in-house bulk IFN-y experiment involved three scenarios: in the 1st scenario, we have used 144 samples for model training and 72 samples for model testing. These samples derived from 6 biological replicates (three per condition) and were injected in 24 and 12 technical replicates for the training and testing, respectively. In the 2nd scenario, 64 HeLa cell cultures (biol. replicates) were cultured individually in a 96-well format, where half of the cultures were treated with IFN-y. Each culture was measured in a single-shot injection. In the 3rd scenario, 315 HeLa cell cultures (biol. replicates) were cultured individually in a 96-format, while we administered a treatment with seven different IFN-y concentrations. Each culture was measured in a single-shot injection.
Data exclusions	Due to problems in data acquisition, two replicates of the in-house training data set and one replicate of the test data set were removed in scenario 1. Two external single-cell data sets had to be filtered on the number of identified peptides per cell to ensure data consistency and reduce missing values.
Replication	All data replications were successful except for replicates mentioned under "data exclusions"
Randomization	In scenario 1, samples from biological replicates and conditions were measured in a randomized order. In scenario 2 and 3, biologically distinct samples of different IFN-y concentrations were measured condition-wise, due to limitations in LC deck configuration. However, since these samples only served to evaluate ML models, potential batch effects had no influence on how data relationships were learned between the experimental conditions. Measurement order of these samples was therefore not relevant to this study.<br>Throughout this work, data sets were randomized during data splitting while keeping label proportions (stratified split) into training, evaluation and test set.
Blinding	Not applicable given the in-vitro and technical focus of the study.

Reporting for specific materials, systems and methods

We require information from authors about some types of materials, experimental systems and methods used in many studies. Here, indicate whether each material, system or method listed is relevant to your study. If you are not sure if a list item applies to your research, read the appropriate section before selecting a response.

Materials & experimental systems

n/a	Involved in the study
☒	☐ Antibodies
☐	☒ Eukaryotic cell lines
☒	☐ Palaeontology and archaeology
☒	☐ Animals and other organisms
☒	☐ Clinical data
☒	☐ Dual use research of concern
☒	☐ Plants

Methods

n/a	Involved in the study
☒	☐ ChIP-seq
☒	☐ Flow cytometry
☒	☐ MRI-based neuroimaging

Eukaryotic cell lines

Policy information about cell lines and Sex and Gender in Research

Cell line source(s)	HeLa cells were obtained from ATCC (CCL-2)
Authentication	Cell line was not authenticated
Mycoplasma contamination	Cell line was tested for Mycoplasma contamination.
Commonly misidentified lines (See ICLAC register)	To the best of our knowledge no misidentified cell lines have been used in this study.

Plants

Seed stocks	n.a.
Novel plant genotypes	n.a.
Authentication	n.a.
