# Supplementary material for: ProFormer: generalizable classification of single-cell and plasma proteomes using deep learning
Source: Nat Commun. 2026 Oct 5;17:10493. doi: 10.1038/s41467-026-78143-2 (PMC13639084; doi:10.1038/s41467-026-78143-2)
Supplement: Supplementary file 3 [file 41467_2026_78143_MOESM8_ESM.pdf]

nature portfolio

Peer Review File

ProFormer: Generalizable classification of single-cell and plasma proteomes using deep learning

Corresponding Author: Professor Jeroen Krijgsveld

This file contains all reviewer reports in order by version, followed by all author rebuttals in order by version.

Version 0:

Reviewer comments:

Reviewer #1

(Remarks to the Author)
Title: Deep learning-based proteomics enables accurate classification of plasma and single-cell samples
This study proposes, ProFormer, a transformer-based deep learning model for the classification of proteomic samples using their tabular peptide profiles, including retention time, mass-to-charge, intensity and ion mobility.
I have the following major concerns:
1. Methods
1.1. If the goal is to classify proteomic samples according to their cell type or disease status, and proteins are a good representation of the cell's functions and phenotype, why don't we just use protein profiles instead of peptide profiles? Proteins function as a whole sequence and peptides generally do not have any function. The reason that we have peptides is just because we want to identify and quantify a protein, but it is too big to sequence, so we have to digest it into smaller peptides (e.g. using trypsin) for identification using LC-MS and then reconstruct the original protein. This whole process happens outside of the cell (except for HLA peptides which are endogenous) and hence the peptides do not provide any extra information regarding the cell identity beyond what provided by the proteins. I think we are making the problem unnecessarily more complicated by using the profiles of dozens of peptides instead of one single profile of their parent protein, and that may add a lot of noise rather than real signals.
1.2. The input is tabular, high-dimensional data with <100 samples VS (thousands of peptides x 4 features). The peptides are grouped to patches using a learnable patch merger that integrate 4 features, and the patches are extracted and reduced to tokens as input to Transformer. Could the authors perform some analysis on the correlation between the peptides and how to reduce the data dimensionality?
1.3. The data is structured tabular data with well-defined features. Thus, we could expect some analysis using simple ML models like logistic regression, or XGBoost, which is well-known to handle structured data. These results could serve a base line before jumping to more sophisticated models like CNNs or Transformer. For instance, the authors showed that ProFormer 3D (0.834) was 3.4% higher than custom CNN (0.799) (Figure 2C). So what is the improvement over simpler models and whether it is worth the efforts?
1.4. For image-based CNNs, the authors have stated that peptide signals in proteomic data are not related to their surroundings, unlike pixels in real images. For Transformer, we can also see that peptides or their patches do not have any meaningful order to treat them as time-series tokens like in languages. In fact, if we perform any permutations along the peptide dimension, the sample identity still remains the same, but the assumptions of CNNs or Transformer models no longer hold. So whether these models are suitable for this type of data?
2. Applications of the classification of proteomic samples
2.1. Single-cell proteomes: All analyses in Table 1 are binary classification tasks, and for each task, the training and testing sets came from the same study. This raises questions about potential generalizations to more complicated classification scenarios and datasets from different studies
2.2. Plasma proteomes: Could the authors explain the potential applications of the classification of severe Covid-19 versus others? From my perspective, it is pretty simple to do a PCR test to confirm Covid-19 and prescribe further treatments based on the patient symptoms and conditions. I would expect some more interesting applications, e.g. cancer blood tests, where it is challenging to determine the disease status of the samples.

(Remarks on code availability)

Reviewer #2

(Remarks to the Author)
In this manuscript, the authors present an interesting approach to classifying proteomic samples using the four feature dimensions available in MS1 spectra. They compare this to existing CNN-based image approaches and report improved performance using their ProFormer transformer-based pipeline. While I see the relevance of this approach and specifically appreciate the effort in benchmarking the existing CNN pipelines, I have several concerns, particularly regarding its clinical relevance, novelty, and methodological choices.
One of my main concerns is the way the authors refer to the MS1 feature dimension as "peptide." More correctly, it should be peptide ion(s) since true peptide identification happens at the MS2 level. The way it's currently written is confusing to readers. This directly connects to a bigger issue: the masking of peptide identities at the MS2 level. Does this mean that only MS1 data from MS2-identified peptides is used? If so, this defeats the entire purpose of ProFormer. If not, it would be valuable to examine whether MS1-level features that were not identified at MS2 still hold discriminatory power. This could substantially improve the manuscript's currently weak novelty.
Another issue is that the authors present basic machine learning concepts as groundbreaking insights, which I find frustrating. For example, between lines 262–267, they highlight that reducing noise and increasing dataset size improves model performance, something straightforward and well understood by anyone with basic ML knowledge.
I also question the classification choices. The authors classify severe COVID-19 vs. all other conditions, but why stop there? A one-vs-rest classification for mild vs. all, influenza vs. all, or sepsis vs. all would have been a straightforward extension and greatly improved the model's relevance. I believe this should be added, and it would be necessary for any follow-up review.
Lastly, I feel this paper suffers from deep-learning hype. Although I work in bioinformatics, I believe the simplest solution is often the best. The authors compare their method to a Random Forest (RF) model, but the comparison is flawed. First, feature selection was done using ProFormer outputs, preventing the RF from independently selecting its optimal features. RF models can handle all available information, but this capability was artificially restricted here. Second, the RF model was not properly hyperparameter-tuned or cross-validated, limiting its performance and making the comparison unfair. Given these issues, I am not convinced that deep learning is necessary for this classification task, and the manuscript does not make a strong case for its advantages.
Additional Issues:
• Conceptual Contradictions and vagueness:
Line 55 claims peptide signals in proteomic data are not related to their surroundings, which is not true and directly contradicts line 90, where the advantage of ProFormer is said to be its ability to capture contextual information.
The patch merger approach (line 93) is unclear. How does this differ from CNN-based binning? The figure does not clarify this.
Lines 130–131 mention "verifying the technical equality of features between datasets" without explaining what was actually verified.
• Methodological Concerns:
Is it possible that the high discriminatory power between studies (line 204) might simply result from differences in cell size and overall protein content rather than true proteomic differences at the MS1 level?
The random seed for the t-SNE visualization is missing, making the image non-reproducible.
Good ML/DL practice would be to perform hyperparameter tuning in triplicate to account for random initialization effects.
The manuscript does not discuss whether the authors handled class imbalances, which is highly relevant in classification tasks. Reporting additional metrics like the F1-score alongside AUROC would clarify model performance.
• Potential Issues in the Code:
No early stopping, increasing the risk of overfitting.
The num_heads parameter requires further validation—how is this evenly divided across embedding dimensions?
It is unclear how testing is done post-hyperparameter tuning. The tune.py file does not perform this step, and I see no reference to it in main.py.
Overall, this paper lacks strong bioinformatics grounding, needs substantial clarification on its core methods, and does not clearly justify its relevance or novelty. A rigorous comparison with classical ML approaches, better discussion of ProFormer's actual advantages, and a clearer justification for using deep learning are needed.

(Remarks on code availability)
The code was well structured and clearly written and documented, any remarks I have that are unclear are mentioned in my general comments.

Reviewer #3

(Remarks to the Author)
In this manuscript, Krull, Kühn et al. present a transformer-based classifier for the classification of MS-based proteomics data. They demonstrate the performance of the classifier on a wide range of data: (1) bulk measurement of HeLa cells in two different conditions, (2) single cell experiments, and (3) plasma samples from patients aiming to identify severe cases of COVID19. Overall, the approach is interesting and could provide new avenues in the field of sample clustering, but I have concerns on the manuscript that need to be addressed before publication.

Main comments
1- In the Bulk experiment, it is not clear how 'train set' and 'test sets' are designed. The text is inconsistent on this, and sometimes states '273 samples' where it seems that it was 273 acquisitions of six samples. The figures are also deceiving in

the sense that they present multiple acquisitions of the same samples as replicates. The authors need to rule out that redundancy in the measurement is biasing the results or their perception by the reader. It is also not clear whether the same cell lines were used for training and testing. If the same cell lines are used, I fear that the model recognizes cells from its training data rather than the condition of treatment of the cells as the authors claim. It is further not clear what happened to the cells during six months (cultivation or frozen?). While the authors have a strong point that transferability of published models is a concern, using the same cells six months later is not a convincing approach to evaluate transferability. Overall, I find the experiment with three lines of HeLa cells poorly suited to demonstrate the performance of the tool. There are thousands of available data sets online, why not use these to get better power and more diversity of experimental setups for bulk/tissue analysis?
2- When comparing CNNs with their approach, the AUROC numbers can be deceiving as they look impressive but discrimination power displayed on the ROC curves is really poor and the gain over a custom CNN is not evident. In Fig 1D, the threshold is conveniently placed at 10% so that the 50% sensitivity - which is really bad - is higher than the other approaches. Moving the threshold a little bit on either side tells a very different story. Overall the performance of the classifiers seem very volatile - which is not all too surprising with three cell lines - and questions again the suitability of these data to evaluate the algorithm.
3- The comparison with CNN-based classifiers seems a bit unfair, in the sense that these tools use (all) raw data, where the authors heavily pre-process the data with DIA-NN/MaxQuant. It is not clear whether the gain is really from the transformer/attention architecture or from focusing on high-quality peptide-derived features. The comparison with a random forest approach on the covid data helps resolving this. Please compare the performance of ProFormer with random forest, xgboost, or equivalent for the other data sets (bulk and single cell) as well, and extend Table 1 accordingly. In the case of Fig 2E, if there is such a clear separation in PC1, simple methods should be able to classify the samples?
4- It is not always clear how the peptide features are obtained when reading the text. Please make sure that it is clear to the reader how the features provided to the classifier were obtained and what thresholds were used. The wording of the text is not always clear that the features are not derived from MS data alone, but from the results of a database search and quantification.
5- The authors claim that their approach is accurate and fast, with promising applications for clinical studies. It would be good to compare the accuracy of their results with values expected in a clinical setting. When discussing the speed and ease of execution, the authors seem to forget that their approach requires heavy pre-processing of the data. They need to account for the time spent running MaxQuant when comparing to e.g. CNNs on raw data. On the spectrum of proteomics-based patient stratification methods, the approach of the authors requiring sample preparation + LC-MS + MaxQuant + Transformer-based analysis seems rather complex and time consuming compared to other approaches like Olink + LightGBM (doi.org/10.1038/s41591-024-03164-7) and it is not clear whether the analytical effort is worth the gain. It would be good to have some considerations on this to put the claims of the authors in context.

Minor comments
6- When discussing proteins on supp fig 1E, please annotate the protein(s). "specific up-regulation of peptides associated to IFN-γ-responsive proteins". It is not clear what sample is displayed or whether the data are aggregated across samples.
7- Table 1: please extend with sensitivity at 5% error.
8- Single cell data: The motivation to build a classifier is unclear. Is t-SNE/UMAP, as used to train and evaluate the prediction, not already doing the job?
9- Single cell data: When going through the results it is not clear to the reader how missing values are handled and whether this can influence the prediction.

(Remarks on code availability)
The code was shared by the authors as snapshot of their repository. It is well organized and clear. I did not try to run it due to limited time. I look forward to seeing it online.

Version 1:

Reviewer comments:

Reviewer #1

(Remarks to the Author)
The authors have fully addressed all of my concerns raised in the first round of review. I appreciate their efforts to revise and improve the manuscript. I have no further comments and recommend the manuscript for publication.

(Remarks on code availability)

Reviewer #2

(Remarks to the Author)
I am very pleased with the authors' replies and with the clear improvements they have made to the manuscript, figures, and code. They have adequately addressed the majority of my and the other reviewers' comments, and the manuscript has been significantly improved.
I have three remaining concerns

1. Use of MS signals: It is now clear that the method operates exclusively on MS2-identified signals and their corresponding MS1 level. However, if the long-term goal is to extend this to MS1, I remain concerned about the gap between the training data and real-world MS1 noise. The claim in line 101 that “weighting of patches is learnable…increasing robustness to sources of data volatility” is not sufficiently tested. A demonstration on a use case without prior selection of high-quality MS1/MS2 signals would be necessary to support this claim.
2. Generalization and test set design: The current independent test set is still derived from the same biological samples as the training set. While technically independent, this does not test true biological generalization. Including data from different biological sources would strengthen the evidence.
3. Dimensionality reduction: The manuscript mentions the use of reduction methods in the machine learning pipeline. It is not entirely clear whether this reduction was applied to the patched data or to the raw MS1 data. For a fair assessment of the Proformer architecture, dimensionality reduction should be performed on the patched data, allowing one to separate the benefits of patching from those of the architecture itself.

Addressing these remaining points through additional experiments or, at a minimum, a clearer discussion of the limitations and assumptions would be essential to support the manuscript’s claims.

(Remarks on code availability)

Reviewer #3

(Remarks to the Author)
I thank the authors for taking the time to answer my comments and greatly clarifying the text. After better understanding of how the data were processed, I still have two major concerns on this work.

My first main concern in the initial review was definition what the authors meant with 'samples'. In their answer, the authors clarify that the different analyses represent multiple measurements of the same peptide mixture. The authors make a point that technical replicates capture a proportion of the variance. But we all agree that the technical variance of modern proteomic data sets only represents a minor fraction of the overall variance. I therefore do not think that using hundreds of measurements of the same six cell lines is sufficient to assess the performance of a classifier. It is like when trying to classify people by height, measuring 6 people with a ruler hundred times, and then six month later ask another person to measure the same six people. Even with the addition of noise in silico, this is not sufficient to evaluate the performance of a classifier, and certainly does not prove robustness towards overfitting. In comparison, the work by Cadow et al. that the authors compare against, published in bioinformatics, uses 554 tissue biopsies sampled from 277 patients. I acknowledge that the authors have amended the text to clarify the scope of the experiment, but I still find the experimental design below the expectations of the field, and I find that some claims - e.g. the subtitle "Accurate and robust prediction of a mild sample treatment by the ProFormer" or claims regarding resistance to overfitting - are misleading in the context of multiple injections of the same peptide mixture. The choice of this data set is also surprising in the sense that the authors introduce a mild modification to the proteome, and then all classification models perform poorly, almost indistinguishably. Stronger differences with replicates, e.g. different conditions or dose-response, would have allowed better evaluating and comparing the performance of the models. The authors correctly point that they have analyzed other data sets with other types of application (single cell and patient data), but the analysis of the six HeLa samples represent half of the results section and the fundamental analytical evaluation of the model. These data are not suited for this and another data set needs to be used at the risk of deceiving or misleading the reader.

My third comment concerned the approach itself, and was echoed by all other reviewers: how the data were processed was unclear from the initial submission. I thank the authors for clarifying the text in this version. The authors put their work in the context of sample classification from MS data, both in the introduction and in their answer to reviewers. Tackling this 'search-free' classification problem was also the goal of Cadow et al.. Now the authors conducted searches in order to get the MS1 features, and provided these features to all models. From what I understand it means that the authors used the work of Cadow et al. outside of their application area, providing a limited subset of the data, whereas unless I am mistaking, these tools were made to use entire MS maps. Worse, because the authors relied on searches to extract their features, they did not answer the problem of search-free classification that they set themselves to solve. As pointed by the other reviewers, the prior search defeats the point of the approach - and if it is done anyway what is the point of masking peptide/protein identity? As written in my initial review, I find it deceiving to compare a pure MS classification model with one that relies on prior search, and unless the authors can extract their MS features without a prior search, I do not think that they can claim to have solved the problem of MS1-based classification that has been addressed by Cadow et al..

Unless I am misunderstanding, there are fundamental concerns on the approach and the data used to evaluate it. While I really appreciate the quality of the work, especially with the patching and application to cells and covid data, I cannot support its publication until it is (1) search free and (2) evaluated against a data set that is better suited for the purpose.

(Remarks on code availability)
I have navigated the code, it is clear and well documented. I have not tried to install or run it.

Version 2:

Reviewer comments:

Reviewer #2

(Remarks to the Author)
The authors have addressed all my concerns diligently and thoroughly. I am glad to approve this manuscript now for publication.

(Remarks on code availability)

Reviewer #4

(Remarks to the Author)
As requested, I focused on Reviewer #3's major concerns and the authors' responses, rather than conducting a full re-review of the manuscript.

In my view, the authors have substantially addressed Reviewer #3's concern about the experimental design by adding biologically independent IFN-γ validation and dose-response experiments. These additions strengthen the evidence that the model captures a reproducible biological signal rather than only technical-replicate or batch-specific variation.

However, I do not think the concern about the search-free nature of the approach has been fully resolved. The authors clarify that MS2 information was used for feature detection rather than as direct classifier input, but the workflow still depends on MS2-informed feature extraction. Therefore, the method should not be presented as solving fully search-free raw MS1 classification. For example, the Abstract statement that "we introduce the ProFormer, a tabular transformer pipeline that classifies samples solely by MS1-level features" is misleading, and the discussion of the two major limitations in the Introduction (lines 44–47) is also potentially misleading, as readers may infer that the proposed method addresses these limitations when they appear to remain unresolved.

If the authors narrow the scope to MS2-identification-informed MS1 feature extraction, then an additional issue arises: conventional peptide- or protein-abundance classifiers derived from the same MS2 searches become necessary baselines. Without these comparisons, it is unclear whether the proposed peptide/protein-agnostic MS1 feature representation provides an advantage over standard proteomics feature-based classification using the same search-informed preprocessing.

Overall, I would consider the response to Reviewer #3 only partially satisfactory. The manuscript may be publishable if the authors clearly narrow the claims and include appropriate peptide/protein-based classifier comparisons to demonstrate the value of the proposed approach. If the manuscript continues to frame the method as solving search-free MS1 classification, then Reviewer #3's major concern remains unresolved.

(Remarks on code availability)

Version 3:

Reviewer comments:

Reviewer #4

(Remarks to the Author)
I appreciate the authors' clarification and revisions to avoid implying that ProFormer is fully search-free or MS2-independent. However, one comparative-evaluation concern remains. If the current workflow relies on MS2-informed feature extraction, it is not clear why conventional peptide- or protein-abundance classifiers derived from the same MS2 searches would not be appropriate baselines. Comparing different classifiers using the same ProFormer input representation does not directly test whether the proposed feature representation provides an advantage over standard proteomics feature matrices.

Therefore, I would ask the authors to either include peptide/protein-level classifier comparisons using the same samples, splits, and metrics, or provide a clear rationale for why such comparisons are not appropriate for the goals of this study. In my view, such comparisons would be informative regardless of outcome: better performance would support the added value of the peptide/protein-agnostic MS1 feature representation; comparable performance would help define ProFormer as an alternative representation with potential practical or future methodological advantages; and weaker performance would clarify the limitations of the current implementation. Without either the comparisons or a clear rationale, readers will not be able to determine how the proposed representation relates to standard MS2-search-derived proteomics features, limiting interpretation of the method's current contribution.

(Remarks on code availability)

Open Access This Peer Review File is licensed under a Creative Commons Attribution 4.0 International License, which permits use, sharing, adaptation, distribution and reproduction in any medium or format, as long as you give appropriate credit to the original author(s) and the source, provide a link to the Creative Commons license, and indicate if changes were made.
In cases where reviewers are anonymous, credit should be given to 'Anonymous Referee' and the source.
The images or other third party material in this Peer Review File are included in the article's Creative Commons license, unless indicated otherwise in a credit line to the material. If material is not included in the article's Creative Commons license and your intended use is not permitted by statutory regulation or exceeds the permitted use, you will need to obtain permission directly from the copyright holder.
To view a copy of this license, visit https://creativecommons.org/licenses/by/4.0/

Deep learning-based proteomics enables accurate classification of plasma and single-cell samples

Rebuttal

Reviewer #1

This study proposes, ProFormer, a transformer-based deep learning model for the classification of proteomic samples using their tabular peptide profiles, including retention time, mass-to-charge, intensity and ion mobility. I have the following major concerns:

1. Methods

1.1. If the goal is to classify proteomic samples according to their cell type or disease status, and proteins are a good representation of the cell's functions and phenotype, why don't we just use protein profiles instead of peptide profiles? Proteins function as a whole sequence and peptides generally do not have any function. The reason that we have peptides is just because we want to identify and quantify a protein, but it is too big to sequence, so we have to digest it into smaller peptides (e.g. using trypsin) for identification using LC-MS and then reconstruct the original protein. This whole process happens outside of the cell (except for HLA peptides which are endogenous) and hence the peptides do not provide any extra information regarding the cell identity beyond what provided by the proteins. I think we are making the problem unnecessarily more complicated by using the profiles of dozens of peptides instead of one single profile of their parent protein, and that may add a lot of noise rather than real signals.

Working in proteomics for many years, we fully see the reviewer´s point and understand their reasoning. However, we probably were not sufficiently clear in indicating that the ProFormer approach introduces a new concept that is **complementary** to the traditional workflow, and that it **serves a different purpose**: while a conventional approach as described by the reviewer aims to identify a large number of proteins in a sample to determine which are differentially expressed and to understand underlying biology, the primary task of the ProFormer is to **classify samples** (e.g. health/disease, cell type, etc). Since this classification can occur at the MS1 level while being agnostic of protein identity, the second important implication of the workflow becomes apparent: since proteins do not need to be identified, MS2 spectra can be ignored or do not even need to be recorded, conceptually bringing considerable time savings by shortening LC gradients. In other words, we position the ProFormer approach for high-throughput sample classification. In addition, this approach considers all MS1 signals from an MS experiment, potentially fetching discriminative power from signals overlooked by conventional proteome analysis. Benefitting from the explainability of the ProFormer model, this is exemplified in our data by the discovery of three peptides of the complement protein C3 that show an inversed regulation in severe Covid-19 disease (see Figure 5J and Supplementary Figure 5B), where a specific proteolytic cleavage of C3 might be favored during activation of complement system, which would not have been visible at protein level. In this regard, our presented work proves the feasibility of effective proteome classification based on high-dimensional peptide data using the output of classical proteome analysis where we masked MS2 information. With this, we laid the groundwork for future studies to implement workflows that extract data directly from raw MS1 scans, achieving fast and accurate classification while omitting protein identification.

We have added sections to the introduction and discussion of the manuscript, clarifying the scope of our work, how and why it is different from a conventional proteomic approach, and in particular why we choose to classify samples based on peptide ion data (line 43-46, 456-459).

1.2. The input is tabular, high-dimensional data with <100 samples VS (thousands of peptides x 4 features). The peptides are grouped to patches using a learnable patch merger that integrate 4 features, and the patches are extracted and reduced to tokens as input to Transformer. Could the authors perform some analysis on the correlation between the peptides and how to reduce the data dimensionality?

We agree that our proposed approach of the patch merger deserves additional explanation. Hence, we described the underlying calculations in more detail, and we performed an analysis on the highest weighted peptide ions in each of the generated patches, which has been included as a **new Figure 3** and **Supplementary Figure 3** in the manuscript. Briefly, we found that patches consistently focus on the same signal clusters in all samples, regardless of their sample class. These groups of peptide ions derive from distinct regions in the feature space. While each patch takes all signals into account, we could show that they weigh peptide ions differently based on their localization in the feature space, dividing specific regions among them. This highlights the dynamic nature of reducing dimensions by the patch merger in contrast to a rigid grid binning approach, as for an image pipeline, which moreover operates in multi-dimensional space. We highlighted the contribution of the patch merger in achieving high classification accuracies in our deep-learning pipeline (moved to **new Table 1** in the main text), while this suggest high degree of data fidelity after dimensional reduction. In this context, we further demonstrated that traditional machine learning algorithms substantially profit from the output of the patch merger, when it was tuned before in the ProFormer pipeline (**new Figure 3E**), lending additional evidence to this point.

1.3. The data is structured tabular data with well-defined features. Thus, we could expect some analysis using simple ML models like logistic regression, or XGBoost, which is well-known to handle structured data. These results could serve a base line before jumping to more sophisticated models like CNNs or Transformer. For instance, the authors showed that ProFormer 3D (0.834) was 3.4% higher than custom CNN (0.799) (Figure 2C). So what is the improvement over simpler models and whether it is worth the efforts?

We agree, and we have therefore analyzed the performance of traditional machine learning on our in-house bulk data set (Figure 2), including a logistic regression, random forest and XGBoost algorithm, and compared the results to our ProFormer pipeline (**new Table 1** and **Figure 2C**). Due to high dimensionality of samples, we tested two different approaches for dimensional reduction (Wavelet transform (coif1) and K-means clustering), before flattening data and tuning the respective model.

These analyses indicate that deep-learning methods can significantly improve classification accuracy compared to traditional machine learning, justifying our decision on deep-learning for discriminating samples according to their peptide-level proteomic data.

1.4. For image-based CNNs, the authors have stated that peptide signals in proteomic data are not related to their surroundings, unlike pixels in real images. For Transformer, we can also see that peptides or their patches do not have any meaningful order to treat them as time-series tokens like in languages. In fact, if we perform any permutations along the peptide dimension, the sample identity still remains the same, but the assumptions of CNNs or Transformer models no longer hold. So whether these models are suitable for this type of data?

We agree, and that is why we have criticized the use of CNNs for the processing and classification of proteomic data. Instead, we proposed the use of a transformer, in which we have removed the positional encoding to allow the network to identify relations irrespective of the order of peptides/tokens. Consequently, our ProFormer pipeline dynamically determines its attention regions (see Patch merger, **new Figure 3**), before setting generated tokens into a global context, not restricted to their immediate surrounding, unlike for pixels in CNNs.

We have added this information to the manuscript (line 106-107).

2. Applications of the classification of proteomic samples

2.1. Single-cell proteomes: All analyses in Table 1 are binary classification tasks, and for each task, the training and testing sets came from the same study. This raises questions about potential generalizations to more complicated classification scenarios and datasets from different studies

We agree and this constitutes a general problem when testing AI models for proteomic data, as studies are usually not designed to contain independent data sets. We therefore recorded our own data that consists of a coherent training set and a semi-independent test set, which was acquired six months later, thus displaying a significant batch effect (see Figure 2B), despite being measured on the same instrument and using the same samples. Still, we are well aware that this cannot replace entirely independent model testing, but it served here to demonstrate the accuracy drop of image-based CNN pipelines between validation and test set, while our ProFormer showed robust performance under these circumstances. Hence, we anticipate that the ProFormer will also exhibit greater robustness for more drastic differences, as they might be expected e.g. when comparing data of sample cohorts from different hospitals.

To lend further evidence to this point, we performed an analysis, where we applied Gaussian noise in different intensities to all features (mass-to-charge, retention time and signal intensity) of the test data of single-cell study #6 (**new Figure 4F**), evaluating whether the ProFormer is robust to the resulting deviations from the initial training data. In fact, the ProFormer demonstrated high classification accuracies even with noise ratios of up to 10%, whereas we observed a substantial performance reduction for an equivalently evaluated CNN architecture (ResNet_v2_50). In addition, this experiment illustrates another interesting point: a study design without independent mode testing would have led here to an overestimation of the image pipeline's performance, which reached 100% classification accuracy when tested on dependent data with minimal noise ratio. Notably, this finding coincides with our observations from bulk analysis, where the CNN showed 100% AUC on the validation set, but dropped by about 20% in the test set (Table 1). In contrast, the ProFormer maintained its high accuracy, as discussed above, and we decided to further increase the difficulty by also adding noise to the test set features of this bulk analysis (**new Supplementary Figure 4B**). In this scenario, the ProFormer verified its high robustness,

withstanding cumulative variations of both time-resolved acquisition and artificially introduced noise. These findings underpin our assertion that the ProFormer pipeline is highly resilient to differences in the data sets, while this is not limited to our bulk data set, but can be reproduced also in other data sets, as exemplified in the context of single-cell proteomics data.

2.2. Plasma proteomes: Could the authors explain the potential applications of the classification of severe Covid-19 versus others? From my perspective, it is pretty simple to do a PCR test to confirm Covid-19 and prescribe further treatments based on the patient symptoms and conditions. I would expect some more interesting applications, e.g. cancer blood tests, where it is challenging to determine the disease status of the samples.

We appreciate the reviewer's point. We selected the COMBAT study on Covid-19 because of its public accessibility, large number of samples and involvement of clinical phenotypes beyond a simple binary discrimination between healthy and Covid-19 patients. If a data set of similar size and quality had been available for a cancer blood test, we would have chosen this as a more interesting test case. Regardless, the cohort composition of different clinical phenotypes provides a useful testbed to assess performance of the ProFormer, as it poses a significant challenge to any classification tool. In this context, our data demonstrates that the ProFormer can not only identify the presence or absence of a disease, but also its ability to determine the disease severity, while distinguishing it from differential diagnosis. In fact, recognizing a Corona virus infection can easily be achieved by PCR, but such a test fails to determine the severity of the infection or clinical outcome. In practice, it might be crucial to assess whether a patient is already on the verge of developing symptoms that will ultimately require clinical care, or to identify the leading infection in a patient co-infected with multiple pathogens needing respiratory support. In addition, the authors of the COMBAT study discuss that recognizing severe Covid-19 might be useful for stratifying patients, which might help finding a responsive treatment or compile clinical trials for targeted therapy (see reference [25] in the manuscript). Therefore, we are convinced that the ProFormer's task to discriminate a severe Covid-19 progression from other clinical pictures (including mild Covid-19) reflects a realistic implementation for clinical purposes. That said, we like to emphasize that the main purpose of its use was to showing proof of concept, and agree with the reviewer that more pressing clinical questions are easily imaginable. We would like to add that despite its encouraging results, our work can only provide the basis for creating an end-to-end clinical solution, which was not the prevalent focus of this study.

Reviewer #2

In this manuscript, the authors present an interesting approach to classifying proteomic samples using the four feature dimensions available in MS1 spectra. They compare this to existing CNN-based image approaches and report improved performance using their ProFormer transformer-based pipeline. While I see the relevance of this approach and specifically appreciate the effort in benchmarking the existing CNN pipelines, I have several concerns, particularly regarding its clinical relevance, novelty, and methodological choices.

One of my main concerns is the way the authors refer to the MS1 feature dimension as "peptide." More correctly, it should be peptide ion(s) since true peptide identification happens at the MS2 level. The way it's currently written is confusing to readers.

We agree. We have tried to use a simpler language in our manuscript to make the topic accessible to a broader audience, which is why we have simplified to the word "peptide". Still, since this might confuse experienced readers, we have adapted our wording throughout the manuscript.

This directly connects to a bigger issue: the masking of peptide identities at the MS2 level. Does this mean that only MS1 data from MS2-identified peptides is used? If so, this defeats the entire purpose of ProFormer. If not, it would be valuable to examine whether MS1-level features that were not identified at MS2 still hold discriminatory power. This could substantially improve the manuscript's currently weak novelty.

The reviewer raised a valid point. Still, we would like to note that the purpose of our presented work was to prove the concept of MS1-based classification based on high-dimensional peptide data, while we envision it to be applied to MS1-only raw data in the future directly. Such an approach, however, requires ways to access these data to select, integrate and align signal peaks, then to decide on suitable quantifications and best-performing filters. Resulting data tables will likely be much larger than usual, including significant amounts of noise, which will require a lot of testing and optimization, but can/will be an aim for the future. That said, having access to MS2 data in the used data allowed us to unmask peptide identity at the back-end to evaluate several performance metrics of the ProFomer shown in the manuscript (e.g. explainability), while not jeopardizing its core concept.

Looking ahead, we agree to the reviewer's thoughts that signals not identified at MS2 might still hold discriminatory power, and it could therefore be explored whether these (unidentified) signals allow additional insights into e.g. disease mechanisms when deemed valuable for AI pipelines. This is a fundamental question that we did not look into specifically, and that likely will require a dedicated study. That said, we touched on this in the manuscript by exemplifying the discovery of three peptides of the complement protein C3 that show an inversed regulation in severe Covid-19 disease (see Figure 5J and Supplementary Figure 5B), where a specific proteolytic cleavage of C3 might be favored during activation of complement system, which would not have been visible at protein level through a conventional proteomic approach.

To clarify the aims of our work, we have made several small changes throughout the manuscript. In particular, we have clarified the use of MS1 data from MS2-identified peptides (line 128-130).

Another issue is that the authors present basic machine learning concepts as groundbreaking insights, which I find frustrating. For example, between lines 262–267, they highlight that reducing noise and increasing dataset size improves model performance, something straightforward and well understood by anyone with basic ML knowledge.

This was not our intension, and we rephrased the respective parts (in particular line 350-353).

I also question the classification choices. The authors classify severe COVID-19 vs. all other conditions, but why stop there? A one-vs-rest classification for mild vs. all, influenza vs. all, or sepsis vs. all would have been a straightforward extension and greatly improved the model's relevance. I believe this should be added, and it would be necessary for any follow-up review.

We acknowledge that discriminating individual conditions from the rest can improve our understanding of the ProFormer's capabilities, but we would like to note that the underlying COMBAT study was specifically designed to match the number of severe CV-19 patients to other diagnostics. Accordingly, conditions are represented by vastly different numbers of individuals, where e.g. the healthy subgroup makes only around 5% of the patients (25/490 samples). In fact we still have performed the analyses according to the reviewer's suggestion, but results were rather mediocre, likely as a result of this class imbalance in a one-vs-rest classification. For instance, while mild CV-19 constitutes the second-largest group (148/490 samples), the respective model still achieved only around 67% AUC on the test set. However, this case not only suffers from class imbalance, but also from the fact that this task requires discriminating the condition in both directions – from healthy participants as well as from those with a severe disease state. Due to the composition of the COMBAT data set and absence of a condition that allows fair comparison to our model that identifies severe CV-19, we have decided to not include the analysis in our manuscript.

To clarify the COMBAT study design, we added respective patient number per condition to Figure 5A.

Lastly, I feel this paper suffers from deep-learning hype. Although I work in bioinformatics, I believe the simplest solution is often the best. The authors compare their method to a Random Forest (RF) model, but the comparison is flawed. First, feature selection was done using ProFormer outputs, preventing the RF from independently selecting its optimal features. RF models can handle all available information, but this capability was artificially restricted here. Second, the RF model was not properly hyperparameter-tuned or cross-validated, limiting its performance and making the comparison unfair. Given these issues, I am not convinced that deep learning is necessary for this classification task, and the manuscript does not make a strong case for its advantages.

We agree that the use of deep-learning in our work should be evaluated against simpler solutions, in particular traditional machine learning. Therefore, we have included a number of additional models in the manuscript using logistic regression, random forest or XGBoost algorithms, and we compared them to the results of our ProFormer pipeline for the in-house bulk data (**new Table 1** and **Figure 2C**) and three single-cell data sets (**new Figure 4A**). Due to the high dimensionality of peptide data, we moreover tested two different approaches for dimensional reduction, namely Wavelet transform (coif1) and K-means

clustering, before flattening data and tuning models. Of note, we also updated our Supplementary data 1, now indicating the hyperparameter space used for ML algorithms.

These analyses demonstrate that DL methods can significantly improve the classification accuracy for high dimensional peptide data, justifying the selection of such algorithms in our work. As an interesting observation, however, performances of classical ML approached those of DL methods with lower dimensionality in case of SC studies (**new Figure 4A**), surpassing the ProFormer for a dimensionality as low as 1,200 for SC study #6. Nevertheless, such low dimensionality remains an exception, where contemporary proteomic data usually entails 10 – 100 thousands peptide ions and the single-cell field is already entering this range thanks to the constant push towards higher sensitivity.

On a different note, we would like to clarify that the Random Forest model in Figure 5H was only used to validate the discriminatory power of peptide ions, which were deemed important for the ProFormer model according to SHAP values. At no point did we intend to compare performances of these two algorithms in the presented way in the context of the plasma data set.

Additional Issues:

Conceptual Contradictions and vagueness:

Line 55 claims peptide signals in proteomic data are not related to their surroundings, which is not true and directly contradicts line 90, where the advantage of ProFormer is said to be its ability to capture contextual information.

Peptides that have a similar mass may have an entirely different amino acid sequence, and in a proteome-wide context chances are infinitely small that adjacent peaks happen to derive from the same parent protein. The same goes for other physical properties of peptides, such as ion mobility, chromatographic retention time, or charge state. Hence, binning of these signals, as occurs in CNNs, will lead to the combination of biologically unrelated information, and will cause information loss, e.g. when a low-intensity signal from a variable protein is combined with a high-intensity signal of a stable protein. In the context of convolutional neural networks (CNNs), generated pixels are compared to their immediate surrounding, again relying on information that is presumed to be encoded in local relations. In contrast, the transformer architecture within the ProFormer allows to globally compare the dependencies of tokens regardless of their localization, which we have described in the manuscript as its ability to capture contextual information, not to be confused with providing the model with a semantic context.

We admit that these might be ambiguous terms, and we have therefore adapted our wording at the respective sites in introduction (line 95) and discussion of the manuscript (line 473).

The patch merger approach (line 93) is unclear. How does this differ from CNN-based binning? The figure does not clarify this.

In contrast to a binning approach, in which proximate signals are binned according to a fixed grid, the patch merger produces a number of patches that combine information of all signals with different weightings. Since weights are learnable, this approach allows to adjust the focus of each patch, optimizing

the output of the patch merger as input for the subsequent transformer. The process of defining a patch's focus is still driven by signal proximity, but selection of attention areas is dynamic, and they are not restricted to certain sizes or forms, while also allowing the combination of multiple regions in one patch.

We have updated Figure 1B to improve clarity of the patch merger approach. In addition, we have investigated and illustrated the mechanism of the patch merger further, and results were summarized in the **new Figure 3** and **Supplementary Figure 3**. The main text of the manuscript was extended accordingly.

Lines 130–131 mention "verifying the technical equality of features between datasets" without explaining what was actually verified.

This statement was meant to introduce the subsequent comparison of feature distributions and PCA analyses for the in-house IFN-gamma dataset.

We have clarified the respective sentence in the manuscript (line 142-145).

Methodological Concerns:

Is it possible that the high discriminatory power between studies (line 204) might simply result from differences in cell size and overall protein content rather than true proteomic differences at the MS1 level?

This is indeed a possible explanation, since different cell types are among others characterized by different cell sizes. However, more fine-grained discriminations need to be based on more than just these global aspects, and it is well established that proteome compositions differ vastly between different cell types. With this in mind, we expect that the ProFormer would focus on the most apparent aspect with the highest discriminatory power. In fact, we also obtained high classification accuracies for distinguishing cell cycle stages or differentiation trajectories from the same cell type, in which different cell sizes are playing only a subordinate role and are present in both classes.

The random seed for the t-SNE visualization is missing, making the image non-reproducible.

We have altered the initialization process from "random" to "PCA" to increase global stability and allow reproducibility of t-SNEs. All t-SNEs plots were updated accordingly.

Good ML/DL practice would be to perform hyperparameter tuning in triplicate to account for random initialization effects.

We agree and admit we have not done it due to time and resource constraints. However, we re-evaluated our result by again tuning our models for the IFN-gamma bulk data set (Figure 2) and the COMBAT data set (Figure 5) in triplicates, where we achieved 83.7% AUC (instead of 84.0% AUC) and 85.1% AUC (instead of 86.3% AUC), respectively. Consequently, we also do not expect a high deviation from the indicated accuracies in the manuscript for other data sets.

The manuscript does not discuss whether the authors handled class imbalances, which is highly relevant in classification tasks. Reporting additional metrics like the F1-score alongside AUROC would clarify model performance.

Most of our data sets did not contain significant class imbalances, therefore we believe that AUC alone can serve to evaluate the resulting models. However, in the COMBAT data set, severe CV-19 class was slightly underrepresented (229/490 samples), and we therefore have used a weighted sampling approach together with added weights to the loss function, as already described in the manuscript (line 654-658). Nevertheless, we agree with the reviewer that in this case reporting F1-score would contribute to the clarity of the model performance. Therefore, we have tuned the F1 threshold on the cross-validation set of the COMBAT classifier and added this metric to the results in Figure 5C and the main text.

Potential Issues in the Code:

No early stopping, increasing the risk of overfitting.

Due to the difficulty of integrating early stopping into a 5-fold cross-validation framework, specifically in the context of limited samples, we opted for a fixed number of 25 training epochs. At this number, we observed a convergence behavior across folds, showing performance stabilization for a series of models. Model overfitting is not expected to arise with such few epochs, and ProFormer models across the manuscript demonstrated test set results in agreement with performances evaluated on the validation data. In contrast, early stopping could have introduced inconsistent training durations across folds and might have been overly sensitive to noise in small validation splits.

The num_heads parameter requires further validation—how is this evenly divided across embedding dimensions?

The num_heads parameter in multi-head attention controls how the embedding dimension (embed_dim) is split across parallel attention mechanisms, or “heads.” Specifically, the embedding is divided evenly such that each head operates on a subspace of size head_dim = embed_dim / num_heads. This division enables each head to learn distinct feature interactions within the input.

Because of this, embed_dim must be divisible by num_heads. If it isn't, the code will raise an error, as the attention mechanism cannot evenly allocate the feature space across heads.

It's important to note that multi-head attention operates across the sequence dimension, i.e. it compares rows in the input, not columns (features). For each input row, the model computes query, key, and value vectors, which are then processed in parallel by the attention heads. While each head focuses on a different portion of the feature space (columns), attention weights are computed between rows, enabling the model to learn dependencies across the sequence.

It is unclear how testing is done post-hyperparameter tuning. The tune.py file does not perform this step, and I see no reference to it in main.py

Data files were split into a training and test set (see Supplementary Data 1). As proteome data sets typically comprise only a limited number of samples, we decided on a 5-fold cross-validation approach for hyperparameter tuning, involving all training set samples, implemented in tune.py within the tune folder. Since there was no dedicated validation set, model performances were assessed during training based on the mean of all folds. After selecting the best hyperparameters, the final model was retrained on the entire training set and evaluated on prior unseen test data, as shown in __main__.py. If desired, alternative validation procedures can be configured within the pipeline settings.

Overall, this paper lacks strong bioinformatics grounding, needs substantial clarification on its core methods, and does not clearly justify its relevance or novelty. A rigorous comparison with classical ML approaches, better discussion of ProFormer's actual advantages, and a clearer justification for using deep learning are needed.

We hope to have addressed the concerns of the reviewer by our additions to the manuscript and our answers provided above.

The code was well structured and clearly written and documented, any remarks I have that are unclear are mentioned in my general comments.

We appreciate the reviewer's statement.

Reviewer #3

In this manuscript, Krull, Kühn et al. present a transformer-based classifier for the classification of MS-based proteomics data. They demonstrate the performance of the classifier on a wide range of data: (1) bulk measurement of HeLa cells in two different conditions, (2) single cell experiments, and (3) plasma samples from patients aiming to identify severe cases of COVID19. Overall, the approach is interesting and could provide new avenues in the field of sample clustering, but I have concerns on the manuscript that need to be addressed before publication.

Main comments

1 – In the Bulk experiment, it is not clear how 'train set' and 'test sets' are designed. The text is inconsistent on this, and sometimes states '273 samples' where it seems that it was 273 acquisitions of six samples. The figures are also deceiving in the sense that they present multiple acquisitions of the same samples as replicates. The authors need to rule out that redundancy in the measurement is biasing the results or their perception by the reader. It is also not clear whether the same cell lines were used for training and testing. If the same cell lines are used, I fear that the model recognizes cells from its training data rather than the condition of treatment of the cells as the authors claim. It is further not clear what happened to the cells during six months (cultivation or frozen?). While the authors have a strong point that transferability of published models is a concern, using the same cells six months later is not a convincing approach to evaluate transferability. Overall, I find the experiment with three lines of HeLa cells poorly suited to demonstrate the performance of the tool. There are thousands of available data sets online, why not use these to get better power and more diversity of experimental setups for bulk/tissue analysis?

The bulk experiment (Figure 2) consists of six cell culture plates (samples) of the same cell line (HeLa), three of which were treated with IFN-gamma. Peptides obtained from each culture plate were injected 24-times (technical replicates), resulting in a total of 142 injections for the training data set after removing two anomalous runs. Prepared peptides were kept frozen, and we repeated their injection on the same instrument six month later, adding to another 72 injections (12 technical replicates) for the test data set.

We agree that in most applications, data sets intended to be used for AI training are ought to be compiled from a diverse pool of biological replicates, e.g. to cover inter-sample variabilities. Still, technical replicates can serve to capture variations between individual measurements, thus constituting a valuable, additional resource to enhance the model's robustness. We therefore disagree with the perception that technical replicates are redundant by definition. In the context of our bulk experiment, replicate injections were used to model technical uncertainty of LC/MS runs, which we expected to vary considerably between individual measurements and over time. With every replicate, models were confronted with a slightly altered representation of the same biology, anticipating them to learn overcoming even harsher technical differences (experimental data augmentation). In fact, our test set data reflected exactly such a case, where repeating sample injections six month later resulted in a significant batch effect (Figure 2B), which led to a substantial reduction in classification accuracies achieved by image-based algorithms compared to their performances on the training set (moved to **new Table 1** in the main text). In contrast, our ProFormer pipeline demonstrated strong robustness against these technical differences, suggesting that it might also be resilient to other secondary effects. Measurement uncertainties might sometimes be considered as minor source of variability, yet they can dramatically impact AI predictions, as shown by our

experiment. We are well aware that this cannot replace entirely independent model testing, but we would like to state that this is often hard to achieve, and we therefore decided on a focused approach, emphasizing one aspect of test set independence.

Contrary to the reviewer's statement, identifying a proteomic data set that is suitable to train and evaluate an AI pipeline is not straight forward as most studies (I) are not designed to provide either size or the biological coverage required as training material, while combining multiple studies is often limited by data annotation, different experimental conditions or acquisition parameters; (II) do not contain independent data sets that could be used to evaluate models appropriately; (III) do not incorporate ion mobility as a fourth separation dimension in an LC/MS experiment, whose integration to our DL pipeline we intended to highlight. Moreover, (IV) patient data collected in clinical settings are often subject to privacy regulations, which makes it not always easy to reuse these data beyond the scope originally approved. Therefore, we decided to create our own data set with a particular focus on challenging the models by technical differences acquired from the same mass spectrometer (see Figure 2). In addition, we have demonstrated the applicability of the ProFormer pipeline to a series of publicly available data sets in subsequent experiments, challenging it with various, highly heterogeneous data sets.

Reflecting on the reviewer's comment, we have adapted several paragraphs throughout the manuscript, particularly clarifying the setup and our intentions for the design of the in-house experiment (line 119-128, 149-154 & 488-490).

2 – When comparing CNNs with their approach, the AUROC numbers can be deceiving as they look impressive but discrimination power displayed on the ROC curves is really poor and the gain over a custom CNN is not evident. In Fig 1D, the threshold is conveniently placed at 10% so that the 50% sensitivity - which is really bad - is higher than the other approaches. Moving the threshold a little bit on either side tells a very different story. Overall the performance of the classifiers seem very volatile - which is not all too surprising with three cell lines - and questions again the suitability of these data to evaluate the algorithm.

We agree that differences displayed in Figure 2D are not extensive, but improvements of the ProFormer over the ResNet_v2_50 are still evident between the curves, as reflected by an increased AUC of 4%-points. Here, the indicated threshold marks the region of the greatest offsets between the curves, justifying its placement. While the implication of a 10%-error might be subject for discussion, importance of sensitivity and specificity strongly depend on the requirements to the classification, where having higher error or only medium sensitivity can still be acceptable (e.g. when ruling out a disease, see Figure 5C). We would like to further clarify that the scope of our work was to present a robust alternative to existing CNN image pipelines, for which we recognized several weaknesses in the application to proteomic data. In this context, our experiment established that the ProFormer achieved significantly higher accuracies on the test set than 16 other DL pipelines (moved to **new Table 1** in the main text), while we compared it in Figure 2D only to the second-best pipeline.

Beyond bulk data analysis in Figure 2, we previously speculated that the ProFormer also profits from its high resilience in other proteomic data sets. To lend further evidence to this point, we added an analysis, simulating independent model testing for single-cell study #6 by applying Gaussian noise to each feature of its test set (**new Figure 4F**). In this analysis, we evaluated whether the ProFormer is robust to the resulting deviations from the initial training data, and it indeed tolerated noise ratios of up to 10%,

whereas we observed a substantial performance reduction for an equivalently evaluated CNN architecture (ResNet_v2_50). In addition, this experiment illustrates another interesting point: a study design without independent model testing would have led here to an overestimation of the image pipeline's performance, which reached 100% classification accuracy when tested on dependent data with a minimal noise ratio. Notably, this finding coincides with our observations from previous bulk analysis, where the CNN showed 100% AUC on the validation set, but dropped by about 20%-points in the test set (Table 1). In contrast, the ProFormer maintained its high accuracy, as discussed above, and we decided to further increase the difficulty by also adding noise to the test set features of this bulk analysis (**new Supplementary Figure 4B**). In this scenario, the ProFormer verified its high robustness, withstanding cumulative variations of both time-resolved acquisition and artificially introduced noise. These findings underpin our assertion that the ProFormer pipeline is highly resilient to differences in the data sets, while this is not limited to our bulk data set, but can be reproduced also in other data sets, as exemplified in the context of single-cell proteomics data.

3 – The comparison with CNN-based classifiers seems a bit unfair, in the sense that these tools use (all) raw data, where the authors heavily pre-process the data with DIA-NN/MaxQuant. It is not clear whether the gain is really from the transformer/attention architecture or from focusing on high-quality peptide-derived features. The comparison with a random forest approach on the covid data helps resolving this. Please compare the performance of ProFormer with random forest, xgboost, or equivalent for the other data sets (bulk and single cell) as well, and extend Table 1 accordingly. In the case of Fig 2E, if there is such a clear separation in PC1, simple methods should be able to classify the samples?

We excuse the confusion, but both pipelines, CNN and the ProFormer, have used the same data of peptide ion signals identified by data processing with DIA-NN/MaxQuant. Hence, differences observed for these models are indeed related to their differences in data handling.

Nevertheless, we agree to the benefit of comparing the performance of the ProFormer also to traditional machine learning (ML). We have therefore included a number of additional models in the manuscript using logistic regression, random forest or XGBoost algorithms, and we compared them to the results of our ProFormer pipeline for the in-house bulk data (**new Table 1** and **Figure 2C**) and three single-cell data sets (**new Figure 4A**). Due to the high dimensionality of peptide data, we moreover tested two different approaches for dimensional reduction, namely Wavelet transform (coif1) and K-means clustering, before flattening data and tuning models. Of note, we also updated our Supplementary data 1, now indicating the hyperparameter space used for ML algorithms.

These analyses demonstrate that DL methods significantly improved the classification accuracy for high dimensional peptide data, justifying the selection of such methods in our work. As an interesting observation, however, performances of classical ML algorithms approach those of DL methods with lower dimensionality (see SC studies in **new Figure 4A**), surpassing the ProFormer for a dimensionality as low as 1,200 in case of SC study #6. Nevertheless, such low dimensionality remains an exception, where contemporary proteomic data usually entails 10 – 100 thousands of peptide ions and the single-cell field is already entering this range due to the constant push towards higher sensitivity.

On a different note, we would like to clarify that Figure 2E in the manuscript only shows the PCA of the test data. Despite apparent differences in its main component, data of both classes differ by the effect of PC1 in Figure 2B to the training set. We therefore believe that such a case is not easy to solve by simple

ML tools, as it is reflected by our analyses. Moreover, we like to clarify that the Random Forest model in Figure 5H was only used to validate the discriminatory power of peptide ions, which were deemed important for the ProFormer model according to SHAP values. At no point did we intend to compare performances of the ProFormer to a Random Forest in this presented way.

4 – It is not always clear how the peptide features are obtained when reading the text. Please make sure that it is clear to the reader how the features provided to the classifier were obtained and what thresholds were used. The wording of the text is not always clear that the features are not derived from MS data alone, but from the results of a database search and quantification.

We agree that the manuscript's wording was not accurate, and we therefore have emphasized that peptide ion features where obtained from the report tables, not extracted directly from raw data.

The manuscript has been adapted accordingly (line 128-130, 139-142, 260-265 & 376-377). Thresholds for cells in the respective data sets were already given in the methods section (line 628-630).

5 – The authors claim that their approach is accurate and fast, with promising applications for clinical studies. It would be good to compare the accuracy of their results with values expected in a clinical setting. When discussing the speed and ease of execution, the authors seem to forget that their approach requires heavy pre-processing of the data. They need to account for the time spent running MaxQuant when comparing to e.g. CNNs on raw data. On the spectrum of proteomics-based patient stratification methods, the approach of the authors requiring sample preparation + LC-MS + MaxQuant + Transformer-based analysis seems rather complex and time consuming compared to other approaches like Olink + LightGBM (doi.org/10.1038/s41591-024-03164-7) and it is not clear whether the analytical effort is worth the gain. It would be good to have some considerations on this to put the claims of the authors in context.

The scope of our work was to demonstrate that MS1-based classification of proteomic data can be realized with high accuracy and robustness. To this end, we have used conventionally acquired data in our manuscript, where we have relied on peptide ions that were identified via DIA-NN/MaxQuant using their MS2 information for both ProFormer and CNN approaches. However, after showing that only their MS1 data suffices to sample discrimination, MS2 acquisition might no longer be required in such a pipeline, which accounts for most of the measurement and subsequent analysis time in proteomic experiments. Consequently, this work lays the foundation for rapid LC/MS methods (i.e. shortening or even omitting LC separation), focused on MS1 data acquisition, with future plans to implement the ProFormer pipeline with direct raw data analysis. The anticipated increase in analysis speed could surpass current proteomic techniques, including those not based on mass spectrometry. This would enable high-throughput clinical diagnostics with large sample cohorts.

We recognize the value of comparing accuracies and analysis speed expected in clinical settings, but we would like to emphasize that our manuscript constitutes a methodological work aimed to show proof of principle of a conceptually new approach, not claiming to have generated a ready-to-use classifier. Hence, we have adapted the manuscript to clarify the scope of our current work and indicate potential future applications.

# Minor comments

6 – When discussing proteins on supp fig 1E, please annotate the protein(s). "specific up-regulation of peptides associated to IFN-γ-responsive proteins". It is not clear what sample is displayed or whether the data are aggregated across samples.

The label of another protein covered the discussed protein, but we corrected the mistake.

The corresponding plot shows the aggregated results across all training and test samples. We calculated fold changes by dividing the mean peptide ion intensity in both sample classes (control and IFN-γ). We added this information to the figure caption.

In addition, we have added a new Supplementary Figure 1F, emphasizing that peptide ions show similar regulation in both data sets, and displaying them together with their gene names to allow readers to reassure about the up-regulation of IFN-γ-responsive proteins.

7 – Table 1: please extend with sensitivity at 5% error.

We thank the reviewer for their advice and we added the metric to single-cell data (now Table 2).

8 – Single cell data: The motivation to build a classifier is unclear. Is t-SNE/UMAP, as used to train and evaluate the prediction, not already doing the job?

In scenarios where non-linear dimensionality reduction methods such as t-SNE or UMAP can produce distinct clusters, samples might already be classified by distance-based rules. However, this is rarely achievable in complex biological data, since even advanced reduction methods often struggle to resolve subtle biological signatures against a background of substantial biological heterogeneity and technical uncertainty. In our case, this is further complicated by the high-dimensionality of peptide-level proteomic data, and it led to a significant overlap between cells/samples in Figure 4B and 5B. In related cases, overlapping regions might even be expected, but dimensional reduction is also not well suited to handle gradual transitions between cell states, such as those observed in highly plastic cell populations. Therefore, we only applied these approaches to illustrate data structure, serving to estimate the difficulty of a classification, while the non-parametric character of t-SNE and UMAP does not necessarily provide clear decision boundaries to classify previously unseen data. Instead, we used alternative strategies for dimensional reduction as preprocessing steps for subsequent machine-learning classifiers, aiming to preserve information fidelity in the high-dimensional space, with a particular focus on the patch merger.

9 – Single cell data: When going through the results it is not clear to the reader how missing values are handled and whether this can influence the prediction.

Benefitting from the patch merger’s ability to accept inputs of variable length, no imputation or padding is required to specifically handle missing values. Still, presence or absence of missing values can affect the prediction if they systemically appear in the context of either class, e.g. when regulation of a protein happens around the detection limit. We have not specifically investigated this phenomenon for our classification pipeline, but it will be a potential angle for upcoming research.

Handling missing values by the patch merger was already described in the method section (line 637-639).

The code was shared by the authors as snapshot of their repository. It is well organized and clear. I did not try to run it due to limited time. I look forward to seeing it online.

We appreciate the reviewer’s statement.

Deep learning-based proteomics enables accurate classification of plasma and single-cell samples

Rebuttal 2nd Revision

Reviewer #1

The authors have fully addressed all of my concerns raised in the first round of review. I appreciate their efforts to revise and improve the manuscript. I have no further comments and recommend the manuscript for publication.

We appreciate the review and thank the reviewer for their effort.

Reviewer #2

I am very pleased with the authors' replies and with the clear improvements they have made to the manuscript, figures, and code. They have adequately addressed the majority of my and the other reviewers' comments, and the manuscript has been significantly improved. I have three remaining concerns

1. Use of MS signals: It is now clear that the method operates exclusively on MS2-identified signals and their corresponding MS1 level. However, if the long-term goal is to extend this to MS1, I remain concerned about the gap between the training data and real-world MS1 noise. The claim in line 101 that "weighting of patches is learnable…increasing robustness to sources of data volatility" is not sufficiently tested. A demonstration on a use case without prior selection of high-quality MS1/MS2 signals would be necessary to support this claim.

The reviewer's observation is correct that we have used MS1 data of peptides identified through MS2. We did this as a pragmatic approach to focus on our main aim, i.e. interpretation of proteomic MS1 signals, instead of on the processing of raw MS data. The latter would require substantial additional effort to re-purpose existing software or to develop a data processing method that extracts, models and quantifies signals from the MS1 landscape independent of MS2. We clearly see this outside the scope of this manuscript. Yet, having reached our main aim of demonstrating, as a proof of principle, that proteomic samples can be classified by MS1 data, we envision several directions to improve our approach, one of which is the use of MS1-only data. Pursuing this will require significant methodological development that definitely will be the topic of future work.

To still comment on the reviewer´s concern, we expect that the combination and weighting of patched data in the patch merger will aid to overcome measurement noise (random fluctuation). In addition, we anticipate that additional MS1 signals, gained by removing the MS2 identification criterion, will not have a strong impact on the classification: in contrast to CNNs, the ProFormer still relies on discrete signal inputs, which can be filtered e.g. by their intensity to reduce data noise, similar to what is done in every proteomic analysis software. Even if confronted with a significantly higher number of signals per sample, we do not expect a strong effect on the ProFormer's performance, since our data has already demonstrated high compatibility with data of very high dimensionality (in contrast to traditional ML). Therefore, we conclude that use of MS1-only data would likely not have changed the results of our evaluation, and we anticipate similar performance of the ProFormer once an upstream algorithm is in place for detecting MS1 signals.

We agree that the claim in line 101 is not sufficiently addressed by our results, and we have therefore changed the wording to "*Since weighting of patches is learnable, this approach has the potential to represent the data in a more dynamic and information-preserving fashion,* ***and it might thus increase*** *its robustness to sources of data volatility*".

2. Generalization and test set design: The current independent test set is still derived from the same biological samples as the training set. While technically independent, this does not test true biological generalization. Including data from different biological sources would strengthen the evidence.

We agree with the reviewer's comment, and therefore we have extended our manuscript by (1) an independent test set derived from 64 individually cultured HeLa cell samples, half of which were treated with IFN-γ, and (2) a concentration-series experiment comprising 315 individually cultured HeLa cell samples treated with seven different IFN-γ doses. Importantly, this design ensured that samples of both experiments were technically and biologically independent to the cultures used for training. The Proformer achieved high classification accuracies on both new test sets (**new Figure 2F-I and new Supplementary Figure 2)**, thus demonstrating that it learned a generalized representation of the IFN-γ response, dealing with unseen dimensions and different effect sizes (shown in new **Supplementary Data 2**). Notably, other architectures failed to achieve comparable performance on the same task. Remarkably, despite being trained only on control and 100 ng/mL IFN-γ samples, the ProFormer even learned to accurately detect IFN-γ doses down to around 10 ng/mL, producing a biologically adequate sigmoidal dose-response curve between the two sample classes. We included these results as **new Figure 2F-I** and **Supplementary Figure 2** to the revised manuscript.

3. Dimensionality reduction: The manuscript mentions the use of reduction methods in the machine learning pipeline. It is not entirely clear whether this reduction was applied to the patched data or to the raw MS1 data. For a fair assessment of the Proformer architecture, dimensionality reduction should be performed on the patched data, allowing one to separate the benefits of patching from those of the architecture itself.

We appreciate the reviewer's comment, and we would like to clarify our approach: the ProFormer and all other pipelines have received the same list of discrete signals as input, enabling a fair comparison across methods. Because different pipelines process these signals in different ways, we tested Wavelet transform and K-means clustering as dimensional reduction for traditional ML pipelines (which receive tabular input), and we converted signal lists to images as input for CNNs and the Vision-Transformer. In contrast, the ProFormer builds on a patch merger, which combines discrete (patched) signals by a learnable weight function that is tuned during the training process of the subsequent transformer network. Importantly, the patch merger cannot be extracted as neutral, independent transformation and simply be combined with traditional ML algorithms. However, once tuned, the patch merger's output can be used as input for these models. Accordingly, in Figure 3E we demonstrate that, after prior tuning with a transformer network, the output of the patch merger increases the performance of traditional ML compared with deterministic dimensional reductions, e.g. increasing Random Forest performance from 53.4% to 80.3% AUC. Therefore, these results enable a direct comparison of different dimensional reduction strategies and indicate that the tuned patch merger represents proteomic data in more information-preserving manner. In addition, combining the patch merger with a transformer network (ProFormer), further improved the classification accuracy to 84.0% AUC. This highlights that the transformer network outperforms traditional ML algorithms even when based on the same data input.

To avoid confusion in the manuscript, we clarified our approach on the ML pipelines in the result section.

Addressing these remaining points through additional experiments or, at a minimum, a clearer discussion of the limitations and assumptions would be essential to support the manuscript's claims.

We hope that our additional experiments and explanations/discussions have fulfilled the reviewer's expectations.

Reviewer #3

I thank the authors for taking the time to answer my comments and greatly clarifying the text. After better understanding of how the data were processed, I still have two major concerns on this work.

My first main concern in the initial review was definition what the authors meant with 'samples'. In their answer, the authors clarify that the different analyses represent multiple measurements of the same peptide mixture. The authors make a point that technical replicates capture a proportion of the variance. But we all agree that the technical variance of modern proteomic data sets only represents a minor fraction of the overall variance. I therefore do not think that using hundreds of measurements of the same six cell lines is sufficient to assess the performance of a classifier. It is like when trying to classify people by height, measuring 6 people with a ruler hundred times, and then six month later ask another person to measure the same six people. Even with the addition of noise in silico, this is not sufficient to evaluate the performance of a classifier, and certainly does not prove robustness towards overfitting. In comparison, the work by Cadow et al. that the authors compare against, published in bioinformatics, uses 554 tissue biopsies sampled from 277 patients. I acknowledge that the authors have amended the text to clarify the scope of the experiment, but I still find the experimental design below the expectations of the field, and I find that some claims - e.g. the subtitle "Accurate and robust prediction of a mild sample treatment by the ProFormer" or claims regarding resistance to overfitting - are misleading in the context of multiple injections of the same peptide mixture. The choice of this data set is also surprising in the sense that the authors introduce a mild modification to the proteome, and then all classification models perform poorly, almost indistinguishably. Stronger differences with replicates, e.g. different conditions or dose-response, would have allowed better evaluating and comparing the performance of the models. The authors correctly point that they have analyzed other data sets with other types of application (single cell and patient data), but the analysis of the six HeLa samples represent half of the results section and the fundamental analytical evaluation of the model. These data are not suited for this and another data set needs to be used at the risk of deceiving or misleading the reader.

We thank the reviewer for this detailed and constructive comment. We agree that technical replicates of a limited number of cell cultures are not equivalent to biological replicates from independent donors, and we acknowledge this as a limitation. However, we don’t believe that our experiment is below the expectations, given that it uncovered a substantial problem in the classification of proteomic data – batch effects (technical domain shifts) can obscure results by introducing systematic biases to the data, e.g. from preparative conditions, instrument calibration, method parameters, laboratories, and software. In this context, we provided a controlled demonstration of temporal batch-effect robustness, where a 6-month acquisition gap showed a consistent val/test performance of the ProFormer (discrepancy: 0.016–0.047 AUC) compared to the dramatic drop observed in all 16 CNN or Vision-transformers networks (0.201–0.569 AUC). This finding highlights a key challenge in developing proteome classifiers for real-world applications. By comparison, other data sets, no matter how exceptional and rich they are, cannot provide the same insights, when their samples were collected at the same facility, prepared in the same lab and measured in one cohesive batch.

Nevertheless, we agree with the reviewer that a comparison of architectures based on biologically distinct training and test sets would further strengthen the evidence in our work. Therefore, we extended our manuscript by (1) an independent test set derived from 64 individually cultured HeLa cell samples, half of which were treated with IFN-γ, and (2) a concentration-series experiment comprising 315 individually

cultured HeLa cell samples treated with seven different IFN-γ doses. Importantly, this design ensured that samples of both experiments were technically and biologically independent to the cultures used for training. The Proformer achieved high classification accuracies on both new test sets (**new Figure 2F-I and Supplementary Figure 2)**, thus demonstrating that it learned a generalized representation of the IFN-γ response, dealing with unseen dimensions and different effect sizes (shown in new **Supplementary Data 2**). Notably, other architectures failed to achieve comparable performance on the same task. Remarkably, despite being trained only on control and 100 ng/mL IFN-γ samples, the ProFormer even learned to accurately detect IFN-γ doses down to around 10 ng/mL, producing a biologically adequate sigmoidal dose-response curve between the two sample classes. We included these results as **new Figure 2F-I** and **Supplementary Figure 2** to the revised manuscript.

My third comment concerned the approach itself, and was echoed by all other reviewers: how the data were processed was unclear from the initial submission. I thank the authors for clarifying the text in this version. The authors put their work in the context of sample classification from MS data, both in the introduction and in their answer to reviewers. Tackling this 'search-free' classification problem was also the goal of Cadow et al.. Now the authors conducted searches in order to get the MS1 features, and provided these features to all models. From what I understand it means that the authors used the work of Cadow et al. outside of their application area, providing a limited subset of the data, whereas unless I am mistaking, these tools were made to use entire MS maps. Worse, because the authors relied on searches to extract their features, they did not answer the problem of search-free classification that they set themselves to solve. As pointed by the other reviewers, the prior search defeats the point of the approach - and if it is done anyway what is the point of masking peptide/protein identity? As written in my initial review, I find it deceiving to compare a pure MS classification model with one that relies on prior search, and unless the authors can extract their MS features without a prior search, I do not think that they can claim to have solved the problem of MS1-based classification that has been addressed by Cadow et al..

We thank the reviewer for the comments. Indeed the aim of our work was to classify proteomic samples based on their MS1 data profile, which we derived from MS2-containing data sets. This choice was not motivated by a dependence on, or specific interest in, MS2 information. Rather, it reflected a pragmatic decision to leverage existing datasets and established software tools to reliably process the data and extract MS1 signals. In other words, we performed the search to detect features, not to identify peptides. This approach allowed us to focus on our primary objective: the interpretation of MS1 signals for proteomic sample classification, instead of on the processing of raw MS data. The latter would require substantial additional effort to re-purpose existing software or to develop a data processing method that extracts, models and quantifies signals from the MS1 landscape, and aligns this across samples. We clearly see this outside the scope of this manuscript. Yet, having achieved our main aim of demonstrating, as a proof of principle, that proteomic samples can be classified by MS1 data, we envision several directions to improve our approach, one of which is the use of MS1-only (i.e. search-free) data, possibly from data that does not even contain MS2 data. Pursuing this will require significant methodological development that definitely will be the topic of future work.

While the MS2 data were not used in any steps of the Proformer strategy itself, importantly it allowed us to unmask peptide identity and validate that the Proformer actually retrieved biologically relevant

information. We showed this in Figure 5I and J, where the features contributing most to the distinction between Covid-19 and other disease turned out to be peptides that were also identified in the conventional proteomics approach. We want to emphasize that we do not envision this masking/unmasking strategy as part of future applications of the Proformer, however having it at hand serves an important purpose in this manuscript to demonstrate that the ProFormer bases its decisions on information-containing features, and to illustrate its potential in the context of explainable AI.

With same aim but using a different approach, Cadow *et al.* adapted several existing CNN architectures that use images as input, which they produced by converting entire MS1 landscapes (i.e. m/z intensities over time/scan numbers) into heatmaps. Although this approach avoids MS peak detection, it comes with significant shortcomings: To pass images to CNNs, they need to fit a certain format, e.g. 224 x 224 pixels, which requires downsizing (binning) of the initial signal map. This is performed by applying a grid on top of the MS1 map that averages signals that fall within bins (see Supplementary Figure 3). This causes information loss in two ways: by the act of averaging of signals, and by doing so for signals that originate from different proteins but that happen to have similar mass and retention time. Cadow *et al*. recognized this issue as they discussed in their publication.

To overcome this problem, the ProFormer provides an alternative approach that maintains data integrity, i.e. keeping MS1 signals as discrete features without binning or averaging. However this requires prior signal detection, which we achieved using existing software that quantifies MS1 features via peptide identification from MS2 spectra, as explained above. The resulting signal tables subsequently served as input for all evaluated ML pipelines, enabling their fair comparison based on the same data. We agree with the reviewer that this approach does not provide unidentified signals with potential discriminatory power, but it equally hides this information from all classifiers.

Feature detection based on MS1-data only will likely produce noisier data; however, we expect that the combination and weighting of signals in the patch merger will aid to overcome measurement noise, as we have already demonstrated by the addition of *in silico* noise in Figure 4F and Suppl. Figure 5B. Moreover, we do not expect additional MS1 signals to have significant impact on the predictions, either gained by removing the MS2 identification criterion or from oversensitive detection. As we have explained above, CNNs typically value higher abundances more than low-intensity signals (and if they don't, they suffer from quantitative inconsistency), limiting the effect of background noise. In contrast, the ProFormer relies on discrete signals, which can be filtered e.g. by their intensity to reduce noise, similar to what is done in every proteomic analysis software. Even if confronted with a significantly higher number of signals per sample, we do not expect a strong effect on the ProFormer's performance, since our data has already demonstrated high compatibility with data of very high dimensionality (in contrast to traditional ML). Therefore, we conclude that use of MS1-only data would likely not have changed the results of our evaluation, and we anticipate similar performance of the ProFormer once an upstream algorithm is in place for detecting MS1 signals.

To clarify these points and address the reviewer's concern, we have added the following paragraph in the discussion:

In earlier efforts, the classification of proteomic samples has been approached by converting their MS1 spectra into an image-like heatmap followed by classification through deep CNNs [4-8], allowing search-free raw data analysis. However, as J. Cadow et al. already stated [4], this can lead to a loss of information when aggregating biologically unrelated signals within spatial proximity (i.e. representing peptides that

*originate from different proteins). Rigid bin borders can further split signals into neighboring bins, confounding the resulting image and making it prone to data fluctuation. Hence, it remains unclear whether a downstream classifier would learn biologically relevant information or artefacts of the aforementioned issues. In this context, CNNs have proven to learn features of natural images, such as edges and corners [32], which are not present in synthetic images derived from MS data. It is therefore questionable whether the inductive bias of CNNs is at all suitable for MS data. Under these circumstances, we suspect that deep CNN architectures bear a high risk of overfitting, i.e. fail to generalize on the classification task. Contrasting CNNs, our approach seeks to preserve the information content of individual signals, leveraging MS1 peaks as input for the classification, instead of training on synthetic images. Specifically, we adopted a patch merger that dynamically weighs and aggregates discrete MS1 signals in a learnable fashion, which we anticipate will more faithfully represent high-dimensional proteomic data. In fact, we demonstrated that the patch merger produces dimensions that focus on self-determined regions in multi-dimensional feature space that are not limited to shapes or sizes, while allowing combination of more than one region per output dimension (**Figure 3**). We interpret this as evidence that the patch merger processes MS data while avoiding confounding signal inputs. As subsequent DL classifier, we implemented a light transformer network that is based on a self-attention mechanism (**Figure 1**). This architecture is suitable for learning data relationships beyond regional peak patterns, allowing to capture global dependencies without a high number of inner network layers [10]. Given the high dimensionality of proteomics data, this might be particularly helpful, as it saves computational costs and makes the model less prone to overfitting. The resulting ProFormer pipeline utilizes individual signals from MS peak detection, and we leveraged established software tools in this work to reliably process and extract MS1 signals from LC-MS/MS raw data. During this process, peptides were identified from associated MS2 spectra, but this was intended solely to detect MS1 features, not to identify peptides. Importantly, MS2 data was not used at any stage of the ProFormer pipeline, which operates entirely in a peptide and protein-agnostic manner. This strategy was sufficient to establish the ProFormer concept in this work, but we envision further improvements through fully search-free extraction of MS1 features. This will require substantial algorithm development; however, it has the potential to provide richer MS1 data as an input for the ProFormer.*

Unless I am misunderstanding, there are fundamental concerns on the approach and the data used to evaluate it. While I really appreciate the quality of the work, especially with the patching and application to cells and covid data, I cannot support its publication until it is (1) search free and (2) evaluated against a data set that is better suited for the purpose.

We hope to have adequately addressed the reviewer's concerns by our answers given above.

Reviewer #3 (Remarks on code availability):

I have navigated the code, it is clear and well documented. I have not tried to install or run it.

We appreciate the comment.

Deep learning-based proteomics enables accurate classification of plasma and single-cell samples

Rebuttal 3rd Revision

Reviewer #2

The authors have addressed all my concerns diligently and thoroughly. I am glad to approve this manuscript now for publication.

We appreciate the review and thank the reviewer for their effort.

Reviewer #4

As requested, I focused on Reviewer #3's major concerns and the authors' responses, rather than conducting a full re-review of the manuscript.

In my view, the authors have substantially addressed Reviewer #3's concern about the experimental design by adding biologically independent IFN-γ validation and dose-response experiments. These additions strengthen the evidence that the model captures a reproducible biological signal rather than only technical-replicate or batch-specific variation.

However, I do not think the concern about the search-free nature of the approach has been fully resolved. The authors clarify that MS2 information was used for feature detection rather than as direct classifier input, but the workflow still depends on MS2-informed feature extraction. Therefore, the method should not be presented as solving fully search-free raw MS1 classification. For example, the Abstract statement that "we introduce the ProFormer, a tabular transformer pipeline that classifies samples solely by MS1-level features" is misleading, and the discussion of the two major limitations in the Introduction (lines 44–47) is also potentially misleading, as readers may infer that the proposed method addresses these limitations when they appear to remain unresolved.

If the authors narrow the scope to MS2-identification-informed MS1 feature extraction, then an additional issue arises: conventional peptide- or protein-abundance classifiers derived from the same MS2 searches become necessary baselines. Without these comparisons, it is unclear whether the proposed peptide/protein-agnostic MS1 feature representation provides an advantage over standard proteomics feature-based classification using the same search-informed preprocessing.

Overall, I would consider the response to Reviewer #3 only partially satisfactory. The manuscript may be publishable if the authors clearly narrow the claims and include appropriate peptide/protein-based classifier comparisons to demonstrate the value of the proposed approach. If the manuscript continues to frame the method as solving search-free MS1 classification, then Reviewer #3's major concern remains unresolved.

We appreciate the reviewer's comments and are grateful for their assessment that our additional data have strengthened the manuscript, particularly by demonstrating that ProFormer captures meaningful biology, thereby addressing a major concern.

The remaining point relates to the perception that our work is "presented as solving fully search-free raw MS1 classification." We would like to clarify that we do not use the term "search-free," or similar wording, anywhere in the manuscript, nor do we claim to have "fully solved" the classification problem. Indeed, it has never been our intention, at this stage of evaluating the ProFormer, to suggest that the approach is entirely independent of MS2 data. However, after revisiting the manuscript with fresh eyes, as the reviewer has done, we recognize that such an impression could arise from our wording.

To ensure full transparency in our data handling and to avoid potential misinterpretation, we have revised the manuscript accordingly. Specifically, we have removed phrasing that could be understood as implying independence from MS2 data (for example, removing "solely" in the sentence highlighted by the reviewer, as well as similar wording elsewhere). Conversely, we have added explicit statements in several sections clarifying that our analyses are MS2-informed. We have further emphasized this point in the discussion,

where we now clearly state that the current implementation uses MS2-informed MS1 data. We also outline future directions that should be aimed at developing preprocessing strategies that extract MS1-only information for use with ProFormer.

Importantly, all comparisons between ProFormer and other classifiers were performed using the same input data, which, in our view, ensures a fair evaluation. Nonetheless, we agree with the reviewer that this comparison should be revisited once a truly MS1-only preprocessing strategy has been implemented.

Through these revisions, we aim to eliminate any unintended impression that we are claiming MS2-independent analysis at this stage, and instead highlight the design, implementation, and initial applications of ProFormer across multiple use cases. We view the encouraging results presented here as strong motivation to further develop the method toward a workflow that is genuinely independent of MS2 data, which obviously is our long-term goal.

ProFormer: Generalizable classification of single-cell and plasma proteomes using deep learning

Rebuttal 4th Revision

Reviewer #4

I appreciate the authors' clarification and revisions to avoid implying that ProFormer is fully search-free or MS2-independent. However, one comparative-evaluation concern remains. If the current workflow relies on MS2-informed feature extraction, it is not clear why conventional peptide- or protein-abundance classifiers derived from the same MS2 searches would not be appropriate baselines. Comparing different classifiers using the same ProFormer input representation does not directly test whether the proposed feature representation provides an advantage over standard proteomics feature matrices.

Therefore, I would ask the authors to either include peptide/protein-level classifier comparisons using the same samples, splits, and metrics, or provide a clear rationale for why such comparisons are not appropriate for the goals of this study. In my view, such comparisons would be informative regardless of outcome: better performance would support the added value of the peptide/protein-agnostic MS1 feature representation; comparable performance would help define ProFormer as an alternative representation with potential practical or future methodological advantages; and weaker performance would clarify the limitations of the current implementation. Without either the comparisons or a clear rationale, readers will not be able to determine how the proposed representation relates to standard MS2-search-derived proteomics features, limiting interpretation of the method's current contribution.

We agree that comparing the ProFormer to conventional peptide-/protein-abundance classifiers would in principal be interesting; however, we believe that such a comparison does not make an appropriate baseline for our approach, and it therefore exceeds the scope of our work.

Peptide-/protein-abundance classifiers tackle a related yet different challenge in the analysis pipeline. They **seek to eliminate the need for manual data interpretation**; however, this is not strictly used to create generalizing AI classification models, but is mostly applied on isolated experiments. For instance, many studies apply them to validate curated sets of peptides/proteins obtained from statistical analysis, e.g. preparing panels for targeted validation. Others use peptide-/protein-abundance classifiers to explain the model decisions, enriching their statistics by an AI importance metric. Instead, application of peptide-/protein-abundance classifiers beyond individual experiments is limited, since they hardly generalize being subject to strong processing biases per experiment or test sample. Moreover, such processing requires the acquisition of MS2 spectra to identify peptide sequences, hampering measurement throughput and the resulting size of training data.

In contrast, the **ProFormer** addresses a much earlier point of the analysis pipeline, as it **seeks to eliminate the need for data processing and interpretation at once**, while its ability to classify samples only on MS1 features makes it **independent of MS2 acquisition**. Our work thus establishes a proof-of-principle for the direct MS1 raw data analysis, even if it has yet only been demonstrated on MS2-informed MS1 features. This will allow the generation of large training data in the future using truncated or even no MS2 acquisition. We anticipate that such a framework will be far better suited to developing AI classifiers that generalize across diverse settings, enabling broad applicability.

In other words, we believe that comparing our approach to Peptide-/protein-abundance classifiers based on performance metrics would be misleading as it does not consider the methodological advantages of the ProFormer, which come with its own challenges that cannot be appropriately accounted for.
